# Genome-Wide DNA Methylation Analysis during Osteogenic Differentiation of Human Bone Marrow Mesenchymal Stem Cells

**DOI:** 10.1155/2018/8238496

**Published:** 2018-09-10

**Authors:** Yangyang Cao, Haoqing Yang, Luyuan Jin, Juan Du, Zhipeng Fan

**Affiliations:** ^1^Laboratory of Molecular Signaling and Stem Cells Therapy, Beijing Key Laboratory of Tooth Regeneration and Function Reconstruction, Capital Medical University School of Stomatology, No. 4 Tiantanxili, Dongcheng District, Beijing 100050, China; ^2^Department of General Dentistry, Capital Medical University School of Stomatology, Beijing 100050, China; ^3^Molecular Laboratory for Gene Therapy and Tooth Regeneration, Beijing Key Laboratory of Tooth Regeneration and Function Reconstruction, Capital Medical University School of Stomatology, Beijing 100050, China

## Abstract

Bone marrow mesenchymal stem cells (BMSCs) nowadays are regarded as promising candidates in cell-based therapy for the regeneration of damaged bone tissues that are either incurable or intractable due to the insufficiency of current therapies. Recent studies suggest that BMSCs differentiate into osteoblasts, and that this differentiation is regulated by some specific patterns of epigenetic modifications, such as DNA methylation. However, the potential role of DNA methylation modification in BMSC osteogenic differentiation is unclear. In this study, we performed a genome-wide study of DNA methylation between the noninduced and induced osteogenic differentiation of BMSCs at day 7. We found that the majority of cytosines in a CpG context were methylated in induced BMSCs. Our results also revealed that, along with the induced osteogenic differentiation in BMSCs, the average genomic methylation levels and CpG methylation in transcriptional factor regions (TFs) were increased, the CpG methylation level of various genomic elements was mainly in the medium-high methylation section, and CpG methylation levels in the repeat element had highly methylated levels. The GO analysis of differentially methylated region- (DMR-) associated genes (DMGs) showed that GO terms, including cytoskeletal protein binding (included in Molecular Function GO terms), skeletal development (included in Biological Process GO terms), mesenchymal cell differentiation (included in Biological Process GO terms), and stem cell differentiation (included in Biological Process), were enriched in the hypermethylated DMGs. Then, the KEGG analysis results showed that the WNT pathway, inositol phosphate metabolism pathway, and cocaine addiction pathway were more correlative with the DMRs during the induced osteogenic differentiation in BMSCs. In conclusion, this study revealed the difference of methylated levels during the noninduced and induced osteogenic differentiation of BMSCs and provided useful information for future works to characterize the important function of epigenetic mechanisms on BMSCs' differentiation.

## 1. Introduction

Bone is a tissue most commonly damaged by aging or disease, and it has a limited ability for self-repair. On account of the impaired osteoblast function, conventional surgical treatments, including internal fixation and bone grafting, are not so effective to solve such a problem [1–4]. Thus, cell-based therapy is considered as a prospective candidate for the regeneration of damaged bone tissues [5]. In light of their relative ease of isolation, low immune rejection, self-renewing ability, and high multidifferentiation potential, mesenchymal stem cells (MSCs) are deemed as hopeful candidates for cell-based therapy [6–8]. Recently, studies suggest that MSC homeostasis between self-renewal and differentiation is regulated by some specific patterns of epigenetic modifications, including DNA methylation [9, 10].

Studies have illustrated that epigenetic mechanisms control the transcription of key genes during osteogenic differentiation, and histone methyltransferase- (HMT-)/histone demethylase- (HDM-) induced alterations in 3tH3K methylation determine the fate of MSC osteogenic differentiation [11–13]. DNA methylation is one of the characterized epigenetic modifications and plays a pivotal role in MSC osteogenic differentiation [14, 15]. It has been reported that overexpression of ESET (SETDB1, a HMT methylating H3K9 site) deregulates Runx2 and Indian hedgehog (Ihh) in MSC osteogenic differentiation during postnatal bone development [16]. The polycomb complexes (PRCs) regulate chromatin structure, and EZH2-mediated regulation in H3K27me3 during osteogenic differentiation affects the activation of lineage-specific genes [15]. Other studies report that cyclin-dependent kinase- (CDK-) 1 promotes osteogenic differentiation through disruption of the PRC2 complexes, which subsequently activate Runx2 [12]. However, the methylation patterns of whole genomic DNA during the osteoblast differentiation of MSCs are unclear. Thus, understanding the epigenetic mechanisms of MSC differentiation is important in determining MSCs' capacity to differentiate into functional osteoblasts for therapeutic applications.

A previous study suggested that day 7 of the induction was the transit point of osteogenic differentiation of human bone marrow mesenchymal stem cells (BMSCs) [17, 18]. In this study, we evaluated the methylation pattern and the difference of the genome-wide DNA methylation profiles of the noninduced and induced osteogenic differentiation BMSCs at day 7.

## 2. Materials and Methods

### 2.1. Cell Culture and Differentiation

Our human stem cell research abides by the ISSCR “Guidelines for the Conduct of Human Embryonic Stem Cell Research.” Human BMSCs were obtained from ScienCell Research Laboratories Inc. (Carlsbad, CA, USA) and cultured in a humidified environment containing 5% CO_2_ at 37°C. The culture medium was Alpha-modified Eagle's medium (*α*-MEM) (Invitrogen, Carlsbad, CA, USA) which was supplemented with 15% foetal bovine serum (FBS; Invitrogen), 100 U/ml penicillin (Invitrogen), and 100 *μ*g/ml streptomycin (Invitrogen). The culture medium was changed every 3 days. The BMSCs were used in following experiments after 3–5 passages.

Human BMSCs were divided into two groups: the noninduced group (*n* = 3) and the day 7 induced osteogenic differentiation group (*n* = 3). The noninduced BMSCs were cultured as previously described. For osteogenic differentiation, BMSCs were cultured in mineralization-inducing medium by using the StemPro Osteogenesis Differentiation Kit (Invitrogen, Carlsbad, CA, USA) for 7 days.

### 2.2. Reverse Transcriptase Polymerase Chain Reaction and Real-Time Reverse Transcriptase-Polymerase Chain Reaction

TRIzol Reagent (Invitrogen) was used to extract total RNA from BMSCs. In light of the manufacturer's protocol (Invitrogen), cDNA of a 2 *μ*g RNA sample was synthesized through oligo (dT) and reverse transcriptase. Real-time RT-PCR reactions were performed with the QuantiTect SYBR Green PCR kit (Qiagen, Hilden, Germany) and an iCycler iQ Multicolor Real-Time PCR Detection System (Bio-Rad). The bone morphogenetic protein 1 (BMP1) primers were as follows: forward, 5′-GGGTCATCCCCTTTGTCATTG-3′and reverse, 5′-GCAAGGTCGATAGGTGAACACA-3′. The Runt-related transcription factor 3 (RUNX3) primers were as follows: forward, 5′-AGGCAATGACGAGAACTACTCC-3′ and reverse, 5′-CGAAGGTCGTTGAACCTGG-3′. The paired box protein 1 (PAX1) primers were as follows: forward, 5′-TCGCTATGGAGCAGACGTATG-3′ and reverse, 5′-GCTGCCGACTGATGTCACA-3′. Glyceraldehyde-3-phosphate dehydrogenase (GAPDH) was detected as the housekeeping gene and primers were as follows: forward, 5′-CGGACCAATACGACCAAATCCG-3′ and reverse, 5′-AGCCACATCGCTCAGACACC-3′.

### 2.3. Genomic DNA Isolation, Bisulphite Treatment, and Methylation Profiling

Genomic DNA was purified from BMSCs using the Wizard Genomic DNA purification Kit (Promega, Madison, USA) according to the manufacturer's instructions, and 500 ng of genomic DNA was then bisulphite converted using the EZ-96 DNA Methylation Kit (Zymo Research, Irvine, USA) and hybridized to Infinium Human Methylation 450 BeadChIP arrays (Illumina Inc., San Diego, USA) by following the manufacturer's protocol.

### 2.4. Library Preparation and Sequencing

For each sample of DNA sequencing libraries, total DNA was initially quantitated to a concentration of 1 ng/*μ*l and used as starting material by the Qubit 2.0 Fluorometer (Invitrogen, USA). Total DNA was quality assessed on an Agilent Technologies 2100 Bioanalyzer and processed according to the manufacturer's instructions. The samples were processed as recommended except for size selection of the adapter-ligated DNA which was done after instead of before amplification. The resulting libraries were sequenced on the Genome Analyzer IIx using TruSeq SBS Kit v5-GA (FC-104-5001, Illumina). Real-time analysis and base calling were done by Illumina's software packages SCS2.9/RTA1.9 and Off-Line Basecaller v1.9.

### 2.5. Methylation Data Processing

Data extraction and quality control were done in GenomeStudio v2011.1 and the Methylation Module v1.9 (both provided by Illumina). To guarantee the quality of sequencing data (raw data), we filtered Reads with sequenced adapters, Reads which have a number of *N* (uncertain base) >5, and reads that filter length ≤ 50. GenomeStudio provides the methylation data as *β* values: *β* = *M*/(*M* + *U*) (*M* represents the fluorescent signal of the methylation probe; *U* represents the signal of the unmethylated probe). *β* values range from 0 (no methylation) to 1 (100% methylation). The raw methylation data was processed using R (version 3.0.1) and the Watermelon package (version 2.12) as has been previously described [19, 20]. Differential methylation was defined as Benjamini-Hochberg corrected *P* value < 0.01 or <0.05 (differentially methylated loci (DML) and gene/CpG island/promoter, respectively) and a mean methylation difference (Δ*β* score) of 0.15 (15%), as previously reported [15, 21]. For each sample, normalized average beta values for every probe were calculated using BeadArray internal controls with no background subtraction. Visualization was performed in GenomeStudio.

### 2.6. Methylation Pattern and Distribution Analysis

The gene exon region (exon), intergenic region (intergenic), intron region (intron), promoter region (promoter), and UTR region (UTR) were coordinated from UCSC Genome Browsers and coanalyzed with the average methylation level of every CpG cytosine within the range. The CpG methylation levels of various genomic elements were profiled as the high methylation section (>80% CpG methylation), medium-high methylation section (50–80% CpG methylation), medium-low methylation section (20–50% CpG methylation), and low methylation section (<20% CpG methylation).

The distribution of the methylation level in the repeating elements was analyzed by using RepeatMasker's latest repeat library RepBase. Repetitive sequence components mainly include DNA transposons (DNA), long scattered repetitive sequence (LINE), long terminal repeat sequence (LTR), Satellite (Satellite), and short scattered repetitive sequence (SINE). The numbers of repetitive sequences taken into detection were as follows: DNA = 30,313, LINE = 189,395, LTR = 126,331, Satellite = 4016, and SINE = 683,782.

### 2.7. Differential Methylation Site (DMS) and Differential Methylation Region (DMR) Analysis

Using the calculate DiffMeth function, DMR analysis was done in MethPipe software. The main steps are as follows: getting the HMR (hypomethylated regions and hypermethylated regions) region; calculating the differential methylation score of each locus; and obtaining the DMRs. Then, we used a *q*-value cutoff point of 0.01 and the CpG methylation site numbers ≥ 5 with significant differences in the methylation area as reference parameters to filter the DMR data. Moreover, MethDiff of MethPipe software was used to detect the DMSs. The CpG methylation site numbers ≥ 5 in the differentially methylation regions were used as reference parameters of significant differences to filter the totally DMS data. RADMeth is also an order of differential methylation analysis in the MethPipe software. Subsequently, the RADMeth order of the MethPipe software was used to detect the whole genome's DMRs and DMSs. This method uses *β*-binomial regression to analyze DMRs and DMSs. The CpG methylation site numbers ≥ 5 in the differentially methylated regions were used as reference parameters of significant differences to filter the totally DMS data.

### 2.8. Bioinformatic Analysis of Methylation Data

The Gene Ontology (GO) is a database of the International Standard Classification of Gene functions, established by the Gene Ontology Consortium. After DMR screening according to specific thresholds and the structure annotation of the DMR-associated genes (DMGs), the ID of the GO can be found from the database through the associated gene' name or ID, and the ID of the GO may correspond to Term, that is, the function category or the cell location. The software we used in GO enrichment analysis is clusterProfiler, and GO enrichment analysis is based on the hypergeometric distribution. The Directed Acyclic Graph (DAG) is the graphical display of the result of GO enrichment analysis in DMRs. Generally, the first 10 positions of the GO enrichment analysis results are selected as the main node of DAG, the associated GO Term is displayed together by the inclusion relation, and the color depth represents the enrichment degree.

In this analysis, clusterProfiler software was used to analyze the genes related to the differentially methylated regions (DMGs) by using the KEGG database. GeneRatio represents the differentially methylated region correlation in pathway enrichment. The number of DMRs contained in each pathway was counted and the *P* value of a hypothetical test was calculated by a hypergeometric test to indicate that the enrichment degree was higher with the decrease of the *P* value and the *P* value of the hypothetical test. A scatter plot is the graphical display of the results of KEGG enrichment analysis.

## 3. Results

### 3.1. The Methylation Pattern and Distribution Analysis during Osteogenic Differentiation in BMSCs

**Figure 1:**
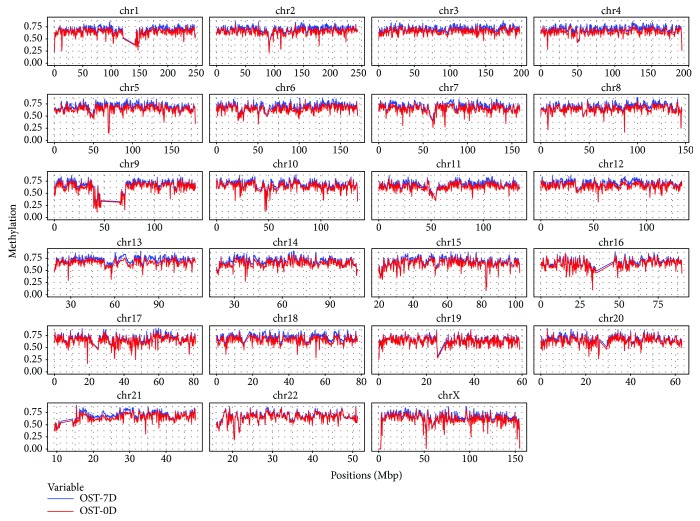
The mapping of the genome-wide DNA methylation level between the osteogenic differentiated group (OST-7D) and the undifferentiated group (OST-0D).

Firstly, the principal component analysis (PCA) was taken to identify the variation of the methylation dataset in the undifferentiated BMSCs and the osteogenic differentiated BMSCs. Then, we present a comparison of genome-wide CpG methylation levels between these two groups. The sample correlation analysis and cluster analysis showed the variation rule of methylation level after osteogenic differentiation induction. To find out the total genome-wide DNA methylation level, the average genomic methylation level of the sample was calculated with 500 K as a fixed window, and the corresponding mapping showed a totally high methylation level in the osteogenic differentiated group (OST-7D) compared with the undifferentiated group (OST-0D) (Figure 1).

**Figure 2:**
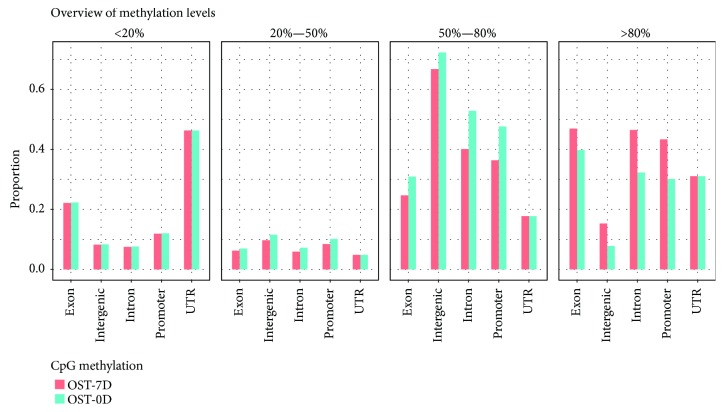
The average CpG methylation level histogram of the various genomic elements.

To investigate the methylation patterns of BMSCs around the gene structure, we analyzed the distribution of methylation level on different gene elements. The histogram showed that the CpG methylation level of various genomic elements was mainly contained in the medium-high methylation section in the OST-0D group and OST-7D group (Figure 2). The CpG methylation level of UTR was similarly in these four methylation sections between the OST-0D group and the OST-7D group, while the CpG methylation levels of exon, intergenic, intron, and promoter had no significant differences in the low methylation section, but showed high levels in the medium-high and medium-low methylation sections and a low level in the high methylation section in the OST-7D group compared with those in the OST-0D group (Figure 2).

**Figure 3:**
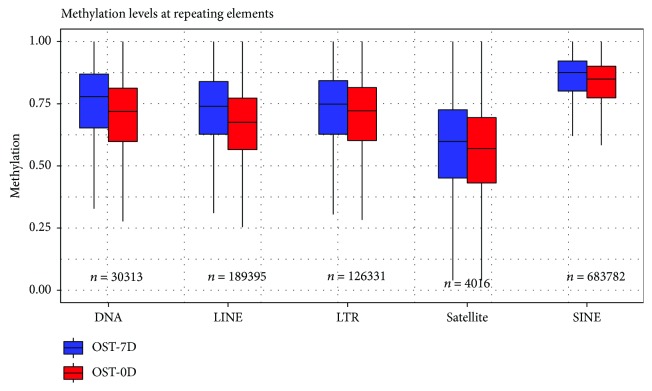
Repetitive sequence component box plot of methylation profiles in various gene structure repeat elements in BMSC genome DNA methylation sequence datasets.

Then, methylation profiles of various repeat elements were analyzed in BMSCs. The results showed that the CpG methylation levels of all repetitive sequences were higher in the OST-7D group compared with those in the OST-0D group (Figure 3). The difference of methylation levels was the biggest in the LINE region and the least in the LTR region between the OST-7D group and the OST-0D group (Figure 3). The distribution of methylation sites on transcription factors (TFs) is shown in Supplementary Figure 1. It shows that TFs such as BCL11A, BCL3, EBF, EBF1, ERa, ERRA, FOXA1/2, HNF4A, HNF4G, IRF4, MAfF, NANOG, POU5F1, PU1, SETDB1, STAT2/3, and ZNF274 were significantly hypermethylated in the OST-7D group compared with those in the OST-0D group, and TFs including AP-2*α*, BAF170, c-Fos, c-JUN, CEBPB, GATA2, GATA3, GR, MAfF, NF*κ*B, P300, SIRT6, TAL1, and TCF4 were significantly hypomethylated in the OST-7D group compared with those in the OST-0D group (Supplementary Figure 1).

### 3.2. DMS and DMR Analysis during Osteogenic Differentiation in BMSCs

We further investigated the methylation difference between the OST-0D group and the OST-7D group at methylation sites and regions. DMS and DMR analyses were taken to investigate the osteogenic differentiation induction caused by differentially methylated sites and regions. Firstly, we used the MethDiff method of the MethPipe software to detect the DMSs and the results showed that there were 1,048,576 significant DMSs with CpG methylation levels that were lower in the OST-0D group compared with those in the OST-7D group, and 512,284 significant DMSs with CpG methylation levels that were lower in the OST-7D group compared with those in the OST-0D group. Moreover, we used the RADMeth method of MethPipe software to detect the DMSs and the results showed that a total number of 256,214 DMSs had been detected in the OST-0D group and OST-7D group, including 14,152 DMSs which were specific to the OST-0D group, 31,968 DMSs which were specific to the OST-7D group, 23,938 DMSs with CpG methylation levels that were higher in the OST-0D group compared with those in the OST-7D group, and 186,156 DMSs with CpG methylation levels that were lower in the OST-0D group compared with those in the OST-7D group.

Moreover, the total numbers of DMRs were 61,352, which was composed of 24,225 DMRs with methylation levels that were lower in the OST-0D group compared with those in the OST-7D group, and 37,127 DMRs with methylation levels that were lower in the OST-7D group compared with those in the OST-0D group using MethDiff methods. After filtering, the total numbers of significantly filtered DMRs were 757, which was composed of 375 DMRs with methylation levels that were lower in the OST-0D group compared with those in the OST-7D group, and 382 DMRs with methylation levels that were lower in the OST-7D group compared with those in the OST-0D group. Then, the RADMeth analysis results showed that a total of 1575 DMRs were identified, containing 1548 DMRs with methylation levels that were lower in the OST-0D group compared with those in the OST-7D group and 27 DMRs with methylation levels that were lower in the OST-7D group compared with those in the OST-0D group. After filtering, the total number of significantly filtered DMRs was 55, which was composed of 50 DMRs with methylation levels that were lower in the OST-0D group compared with those in the OST-7D group and 5 DMRs with methylation levels that were lower in the OST-7D group compared with those in the OST-0D group.

**Figure 4:**
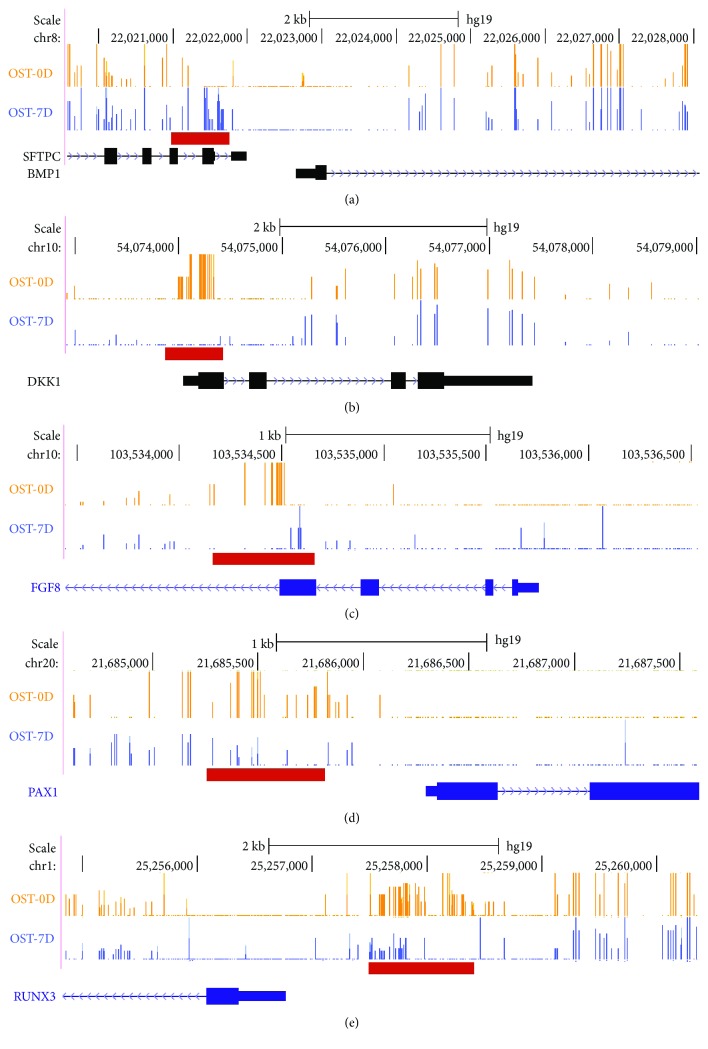
The differential presence of HMR methylation features and DMRs in the gene structure between the OST-0D group and the OST-7D group. (a–e) Differential presence of HMR methylation features and DMRs in the gene structure of BMP1, DKK1, FGF8, PAX1, and RUNX3 in MethBase through the UCSC Genome Browser track hub. Red bars: DMRs between the OST-0D group and the OST-7D group.

**Figure 5:**
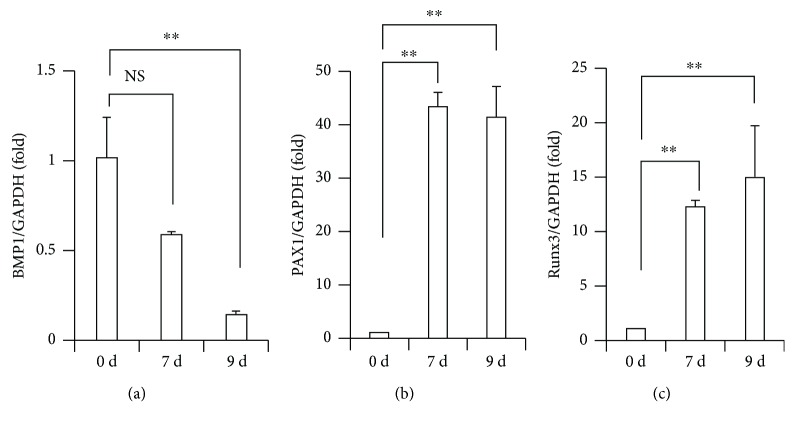
The gene expressions of BMP1, PAX1, and RUNX3 along with the osteogenic differentiation in BMSCs. (a) RT-q-PCR results showed that the expression of BMP1 was decreased after osteogenic induction. (b, c) RT-q-PCR results showed that the expression of PAX1 and RUNX3 was increased after osteogenic induction. GAPDH was used as an internal control. One-way ANOVA test was performed to determine statistical significance. Error bars represent SD (*n* = 3). ^∗^ *p* < 0.05; ^∗∗^ *p* < 0.01.

After obtaining the regions which had differentially methylated levels between the OST-0D group and the OST-7D group, the DMR-related genes were annotated through physical position in the genome and the annotation information of the species. Firstly, a total of 472 genes were identified from the DMR datasets calculated by the MethDiff method between the OST-0D group and the OST-7D group, including 106 genes with DMRs located in the gene promoter (Supplementary Tables 1 and 2). The hypermethylated DMRs which showed high methylation levels in the OST-7D group were involved in 205 genes, including 20 genes with DMRs located in the gene promoter (Supplementary Tables 3 and 4). The hypomethylated DMRs which showed low methylation levels in the OST-7D group were involved in 281 genes, including 86 genes with DMRs located in the gene promoter (Supplementary Tables 5 and 6). Then, the RADMeth analysis results showed that a total of 915 genes were related to DMRs between the OST-0D group and the OST-7D group, including 22 genes with DMRs located in the gene promoter (Supplementary Tables 7 and 8). We have selected five genes including BMP1, DKK1, FGF8, PAX1, and RUNX3. The differential presence of HMR methylation features and DMRs in the gene structure of BMP1, DKK1, FGF8, PAX1, and RUNX3 between the OST-0D group and the OST-7D group was confirmed (Figure 4). We selected 3 candidate genes including BMP1, PAX1, and RUNX3 to analyze their expression with real-time RT-PCR. The real-time RT-PCR results showed that PAX1 and RUNX3 expressions were significantly upregulated at 7 and 9 days after osteogenic differentiation in BMSCs compared with uninduced BMSCs, while BMP1 expression was downregulated at 7 and 9 days after osteogenic differentiation in BMSCs compared with uninduced BMSCs (Figure 5).

### 3.3. Bioinformatic Analysis of Methylation Data during Osteogenic Differentiation in BMSCs

**Figure 6:**
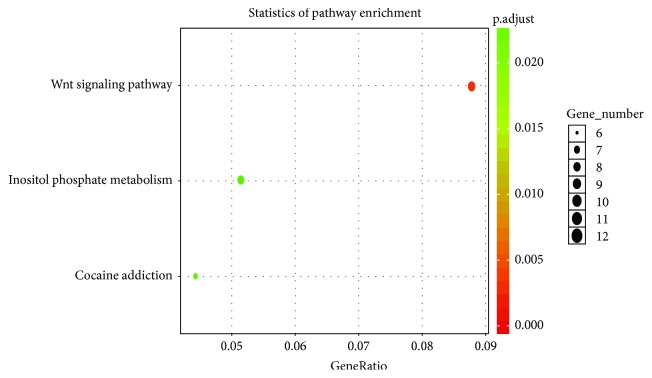
Scatter plot of KEGG enrichment analysis.

According to the selected genes related to DMRs, we calculated the hypergeometric distribution of these genes with some specific branches in GO classification. The enriched GO term histogram of DMGs displays the number and functional classifications of DMGs on GO terms with the major enriched classifications of biological processes, cellular component, and molecular function (Supplementary Figure 2). Furthermore, we analyzed the enriched GO terms of the hypermethylated DMGs and the hypomethylated DMGs (Supplementary Figures 3 and 4). Our results showed that the hypermethylated DMGs were mainly enriched in the biological processes, such as mesenchyme development, mesenchymal cell differentiation, stem cell differentiation, and skeletal system development (Supplementary Figure 3). The hypomethylated DMGs were mainly enriched in three biological processes, including cell fate commitment (Supplementary Figure 4). Then, the DAG graphical display showed the result of GO enrichment analysis in DMGs (Supplementary Figure 5–7). Moreover, the KEGG analysis results showed that the WNT pathway, the inositol phosphate metabolism pathway, and the cocaine addiction pathway were more correlative with the DMRs during the osteogenic differentiation in BMSCs (Figure 6, Supplementary Figure 8–10).

### 3.4. Discussion

Precise regulation of MSC-directed differentiation is also the key for MSC treatment. It is reported that specific and reproducible epigenetic changes were acquired by MSCs during ex vivo culture, and DNA methylation patterns had highly significant differences only at specific CpG islands associated with promoter regions, such as in homeobox genes and genes involved in cell differentiation [22, 23]. Thus, it requires a deep understanding of the DNA methylation regulatory mechanisms driving the differentiation process in MSCs [24].

In this study, we performed a genome-wide study of DNA methylation changes in the noninduced and induced osteogenic differentiation of BMSCs and revealed the dynamics of CpG methylation in the osteogenic differentiation process. It was shown that the average genomic methylation levels and CpG methylation in transcriptional factor regions were increased along with the osteogenic differentiation process in BMSCs. Our results also discovered that the CpG methylation levels in the repeat elements including DNA transposons, LINEs, SINEs, and LTRs had highly methylated levels during osteogenic differentiated induction. These results suggested that the average DNA methylation levels were higher along with osteogenic differentiation in BMSCs, and this epigenetic modification might play an indispensable role in controlling directed differentiation of BMSCs.

We then investigated the DMSs and DMRs between the OST-0D group and the OST-7D group by using the MethDiff method and the RADMeth method of the MethPipe software. There existed some distinction between the two methods which could provide more information for analysis. Usually, the methylation status in the gene promoter will affect the gene expression at the mRNA level; for example, the hypermethylated status in the gene promoter will inhibit the gene expression. Based on these two methods of analysis, we discovered a total of 126 genes with DMRs located in the gene promoter, such as those showing hypermethylated DMRs located in the bone morphogenetic protein (BMP) 1 gene promoter and those showing hypomethylated DMRs located in the paired box protein (PAX) 1 or Runt-related transcription factor (RUNX) 3 gene promoter along with osteogenic differentiation in BMSCs. We also tested the expressions of BMP1, PAX1, and RUNX3 in the noninduced and induced osteogenic differentiation of BMSCs. The results showed that PAX1 expression and RUNX3 expression were significantly upregulated, while BMP1 expression was downregulated at days 7 and 9 in BMSCs after osteogenic differentiation compared with the noninduced BMSCs. BMP1 plays essential roles in osteogenesis and ECM formation and its mutation causes a change in osteoblast morphology and a reduction of osteoblast adhesion to the compromised bone matrix which lead to the delay of ossification and the malformation of bone structures [25]. RUNX3 acts as an essential downstream modulator of the BMP9-induced MSC osteogenic differentiation and matrix mineralization and regulates the inhibitor of differentiation (Id) 3, distal-less homeobox (DLX) 5, RUNX2, and phosphorylation of Smad1/5/8 [26]. PAX1 is a sclerotomal marker in MSC differentiation which is induced by Sonic hedgehog (Shh) signaling, and Noggin encoded bone morphogenetic protein could block its inducted activation in the same way as BMP2/4 [27]. Together, these results confirmed that the methylation status within the gene structure affected the gene expression in different stages along with osteogenic differentiation, and the gene plays a different role for regulating the function of MSCs in different stages. The osteogenic differentiation process of MSCs is regulated by different genes, signaling pathways, and their crosstalk.

Osteogenic differentiation involves a variety of signaling pathways and factors [21]. Using bioinformatics analysis, we then screened for the important pathways involved in the DNA methylation mechanism of BMSCs' osteogenic differentiation. By GO term analysis, our results showed that the hypermethylated DMGs were enriched in the mesenchyme development, mesenchymal cell differentiation, stem cell differentiation, skeletal system development, and so on, while the hypomethylated DMGs were enriched in cell fate commitment, and so on. Research has reported that cytoskeletal protein binding was enriched in the differentially expressed genes (DEGs) in the process of MSC osteogenic differentiation and adipocytic differentiation [28]. Other studies analyzed the dataset of hMSCs differentiated into osteoblasts and these studies report that skeletal development is a specific example of a heterogeneous GO class [29]. In addition, genes involved in mesenchyme development, mesenchymal cell differentiation, and stem cell differentiation might be associated with the differentiation function of MSCs. These suggest that genes in different enriched GO terms are associated with the osteogenic differentiation of BMSCs and may act as specific markers to determine MSC osteogenic differentiation.

Moreover, the KEGG analysis results showed that the methylation-related genes were mainly involved in the WNT pathway, inositol phosphate metabolism pathway, and cocaine addiction pathway during the osteogenic differentiation in BMSCs. Recently, studies have revealed that the Wnt/*β*-catenin pathway qualifies in regulating miscellaneous differentiation processes of MSCs such as the odontoblast-like differentiation of dental pulp stem cells (DPSCs) or osteogenic differentiation of adipose-derived stem cells (ADSCs) [30]. The Wnt pathway regulates the osteogenesis of BMSCs and osteoprogenitor cells in mice [31–35]. Interestingly, in our study, the genes including Wnt, Dkk, LRP-5/6, Nkd, Apc, *β*-Trcp, TCF/LEF, CtBP, and PPAR-*γ* in the canonical Wnt/*β*-catenin pathway showed dynamic methylation modification along with the osteogenic differentiation process. In addition, the genes including prickle in the Wnt/planar cell polarity (PCP) pathway and Wnt5, NFAT in the Wnt/Ca^2+^ pathway showed dynamic methylation modification along with the osteogenic differentiation process. Taken together, the methylation modification might influence the key nodes of the Wnt signaling pathway and its function in the MSC osteogenic differentiation process.

As the most important stereoisomer of inositol, myoinositol has pivotal roles in cell metabolism and is the precursor of all inositol compounds, including phosphoinositides (PI), inositol phosphates (InsPs), inositol sphingolipids, and glycosylphosphatidylinositols. Inositol compounds are essential for gene expression, trafficking, signal transduction, and membrane biogenesis [36]. In the present analysis, inositol-trisphosphate 3-kinase A (ITPK-A, EC 2.7.1.127), inositol-3-phosphate synthase 1 (Isyna1, EC 5.5.14), inositol-1-phosphate synthase (Ino1, EC 5.5.1.4), phosphatidylinositol-4-phosphate 5-kinase type 1 beta/gamma (PIP5K1-B/C, EC 2.7.168), phospholipase C eta 2 (PLCH2, EC 3.1.4.11), and synaptojanin 2 (SYNJ2, EC 3.1.3.36) showed a change in the dynamic methylation modification along with the osteogenic differentiation process. Through the study of soluble InsPs production in undifferentiated and spontaneously differentiated hESCs, a considerable decrease of phosphorylated InsPs and an increase of total PI were shown which might affect the Akt signal ligand and regulate several kinds of second messenger properties. Moreover, the expression of ITPK-A/B was activated upon differentiation [37]. However, the correlation between InsPs metabolism and MSC function is still less known. Here, our results advised a possibility of methylation modification upon the mechanisms undergoing InsPs metabolism and BMSC osteogenic differentiation. Further work for elucidating the mechanisms underlying the control of InsPs is expected to have important implications for MSCs' function.

In conclusion, in the present study, our discovery revealed that the average genomic methylation levels and CpG methylation in transcriptional factor regions were increased, the CpG methylation level of various genomic elements was mainly in the medium-high methylation section, and CpG methylation levels in the repeat element had highly methylated levels along with the osteogenic differentiation in BMSCs. By further analysis, we also revealed that candidate genes and signaling pathways might be regulated by methylation status within the gene structure along with osteogenic differentiation, and then play the important role for regulating the function of MSCs. This work will provide useful information for future works to characterize the important function of epigenetic mechanisms on BMSCs' differentiation.

## Data Availability

The data used to support the findings of this study are included within the article.

## Abbreviations

MSCs: Mesenchymal stem cells
BMSCs: Bone marrow mesenchymal stem cells
HMT: Histone methyltransferase
HDM: Histone demethylase
HMRs: Hypomethylated regions and hypermethylated regions
DMRs: Differentially methylated regions
DMSs: Differential methylation site
DMGs: DMR-associated genes
LINE: Long-scattered repetitive sequence
LTR: Long-terminal repeat sequence
SINE: Short-scattered repetitive sequence
PCA: Principal component analysis
GO: Gene ontology
KEGG: Kyoto Encyclopedia of Genes and Genomes.

## Supplementary Materials

Supplementary Materials Supplementary figure 1: the CpG methylation level histogram of transcription factors (TFs) in BMSC genome DNA methylation sequence datasets. Supplementary figure 2: the total enriched GO term histogram of DMGs in BMSC genome DNA methylation sequence datasets. Supplementary figure 3: the enriched GO terms of the hypermethylated DMGs in BMSC genome DNA methylation sequence datasets. Supplementary figure 4: the enriched GO terms of the hypomethylated DMGs in BMSC genome DNA methylation sequence datasets. Supplementary figure 5: the DAG graphical display of the biological process of GO branches' enrichment analysis in DMGs. Supplementary figure 6: the DAG graphical display of the cellular component of GO branches' enrichment analysis in DMGs. Supplementary figure 7: the DAG graphical display of the molecular function of GO branches' enrichment analysis in DMGs. Supplementary figure 8: the KEGG analysis of the Wnt pathway correlated with the DMRs during the osteogenic differentiation in BMSCs. Supplementary figure 9: the KEGG analysis of the inositol phosphate metabolism pathway correlated with the DMRs during the osteogenic differentiation in BMSCs. Supplementary figure 10: the KEGG analysis of the cocaine addiction pathway correlated with the DMRs during the osteogenic differentiation in BMSCs. Supplementary figure 11: the schematic of the work flow for identification of DMRs and DMSs using MethDiff and RADMeth methods. Supplementary Table 1: the genes related to DMRs between the OST-0D group and the OST-7D group as calculated by DiffMeth method analysis. Supplementary Table 2: the genes with DMRs which were found to be located in the gene promoter by DiffMeth method analysis. Supplementary Table 3: the genes related to hypermethylated DMRs in the OST-7D group compared to those in the OST-0D group. Supplementary Table 4: the genes with hypermethylated DMRs in the OST-7D group which were found to be located in the gene promoter. Supplementary Table 5: the genes related to hypomethylated DMRs in the OST-7D group compared to those in the OST-0D group. Supplementary Table 6: the genes with hypomethylated DMRs in the OST-7D group which were found to be located in the gene promoter. Supplementary Table 7: the genes related to DMRs between the OST-0D group and the OST-7D group as calculated by RADMeth method analysis. Supplementary Table 8: the genes with DMRs which were found to be located in the gene promoter by RADMeth method analysis.
