# Supplementary material for: Genome-Wide DNA Methylation Analysis during Osteogenic Differentiation of Human Bone Marrow Mesenchymal Stem Cells
Source: Stem Cells Int. 2018 Sep 10;2018:8238496. doi: 10.1155/2018/8238496 (PMC6151374; doi:10.1155/2018/8238496)
Supplement: Supplementary Materials — Supplementary figure 1: the CpG methylation level histogram of transcription factors (TFs) in BMSC genome DNA methylation sequence datasets. Supplementary figure 2: the total enriched GO term histogram of DMGs in BMSC genome DNA methylation sequence datasets. Supplementary figure 3: the enriched GO terms of the hypermethylated DMGs in BMSC genome DNA methylation sequence datasets. Supplementary figure 4: the enriched GO terms of the hypomethylated DMGs in BMSC genome DNA methylation sequence datasets. Supplementary figure 5: the DAG graphical display of the biological process of GO branches' enrichment analysis in DMGs. Supplementary figure 6: the DAG graphical display of the cellular component of GO branches' enrichment analysis in DMGs. Supplementary figure 7: the DAG graphical display of the molecular function of GO branches' enrichment analysis in DMGs. Supplementary figure 8: the KEGG analysis of the Wnt pathway correlated with the DMRs during the osteogenic differentiation in BMSCs. Supplementary figure 9: the KEGG analysis of the inositol phosphate metabolism pathway correlated with the DMRs during the osteogenic differentiation in BMSCs. Supplementary figure 10: the KEGG analysis of the cocaine addiction pathway correlated with the DMRs during the osteogenic differentiation in BMSCs. Supplementary figure 11: the schematic of the work flow for identification of DMRs and DMSs using MethDiff and RADMeth methods. Supplementary Table 1: the genes related to DMRs between the OST-0D group and the OST-7D group as calculated by DiffMeth method analysis. Supplementary Table 2: the genes with DMRs which were found to be located in the gene promoter by DiffMeth method analysis. Supplementary Table 3: the genes related to hypermethylated DMRs in the OST-7D group compared to those in the OST-0D group. Supplementary Table 4: the genes with hypermethylated DMRs in the OST-7D group which were found to be located in the gene promoter. Supplementary Table 5: the genes related [file 8238496.f1.pdf]

Methylation Levels at TFBS

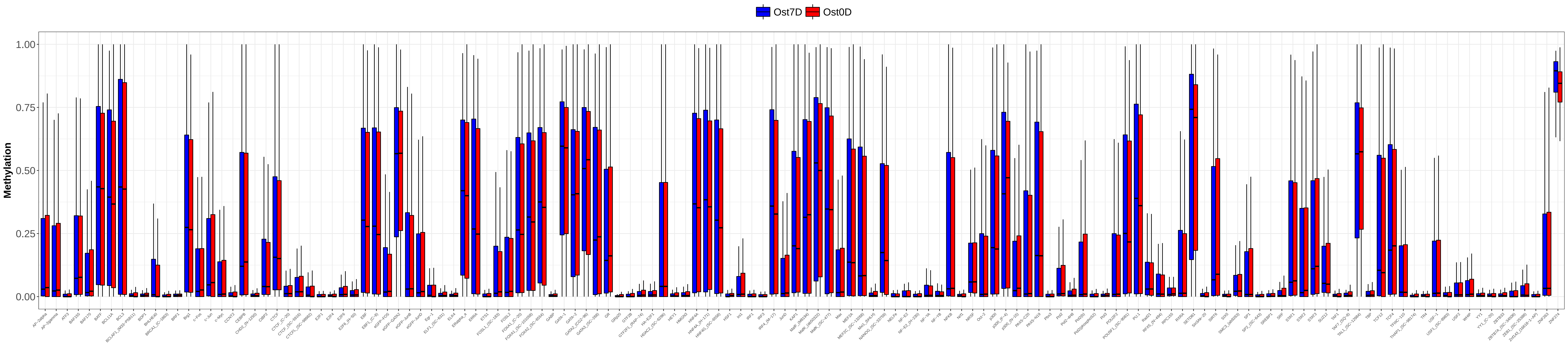

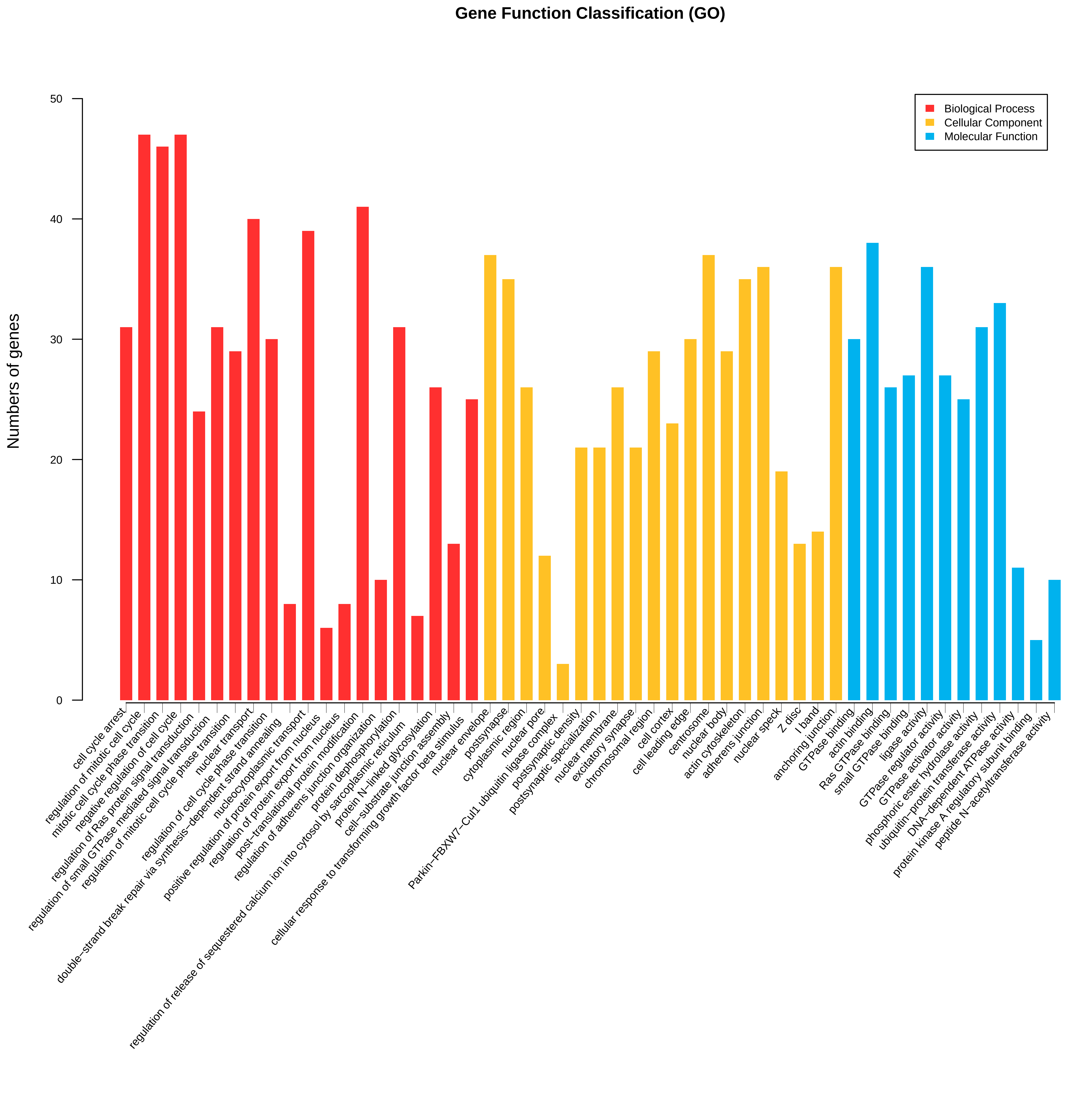
Gene Function Classification (GO)
Numbers of genes
0
10
20
30
40
50
Biological Process
Cellular Component
Molecular Function
cell cycle arrest
regulation of mitotic cell cycle
mitotic cell cycle phase transition
negative regulation of cell cycle
regulation of Ras protein signal transduction
regulation of small GTPase mediated signal transduction
regulation of mitotic cell cycle phase transition
nuclear transport
regulation of cell cycle phase transition
double−strand break repair via synthesis−dependent strand annealing
nucleocytoplasmic transport
positive regulation of protein export from nucleus
regulation of protein export from nucleus
post−translational protein modification
regulation of adherens junction organization
protein dephosphorylation
regulation of release of sequestered calcium ion into cytosol by sarcoplasmic reticulum
protein N−linked glycosylation
cell−substrate junction assembly
cellular response to transforming growth factor beta stimulus
nuclear envelope
postsynapse
cytoplasmic region
nuclear pore
Parkin−FBXW7−Cul1 ubiquitin ligase complex
postsynaptic density
postsynaptic specialization
nuclear membrane
excitatory synapse
chromosomal region
cell cortex
cell leading edge
centrosome
nuclear body
actin cytoskeleton
adherens junction
nuclear speck
Z disc
I band
anchoring junction
GTPase binding
actin binding
Ras GTPase binding
small GTPase binding
ligase activity
GTPase regulator activity
GTPase activator activity
phosphoric ester hydrolase activity
ubiquitin−protein transferase activity
DNA−dependent ATPase activity
protein kinase A regulatory subunit binding
peptide N−acetyltransferase activity

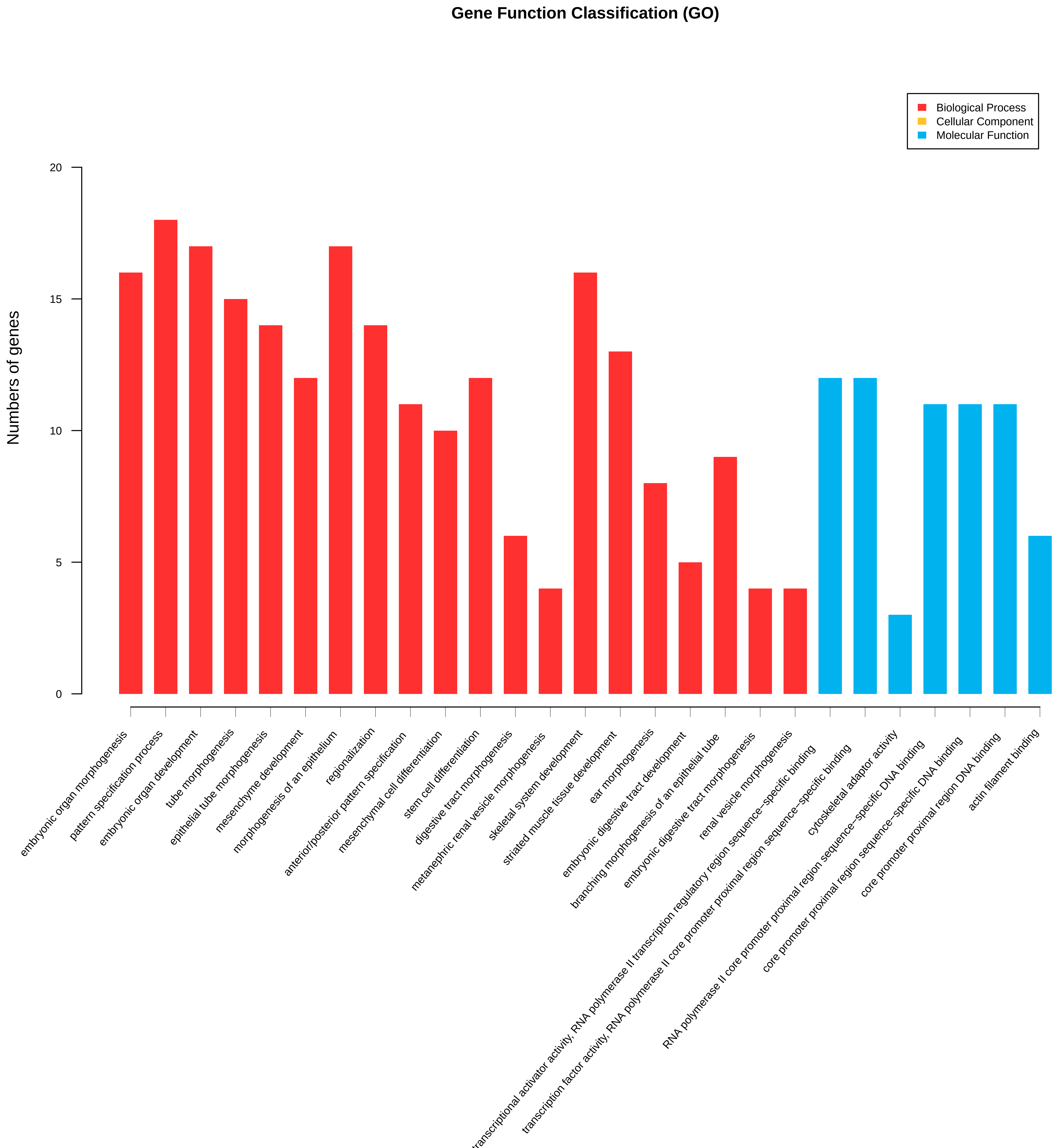

Gene Function Classification (GO)
Biological Process
Cellular Component
Molecular Function
Numbers of genes
0
5
10
15
20
embryonic organ morphogenesis
pattern specification process
embryonic organ development
tube morphogenesis
epithelial tube morphogenesis
mesenchyme development
morphogenesis of an epithelium
regionalization
anterior/posterior pattern specification
mesenchymal cell differentiation
stem cell differentiation
digestive tract morphogenesis
metanephric renal vesicle morphogenesis
skeletal system development
striated muscle tissue development
ear morphogenesis
embryonic digestive tract development
branching morphogenesis of an epithelial tube
embryonic digestive tract morphogenesis
renal vesicle morphogenesis
ranscriptional activator activity, RNA polymerase II transcription regulatory region sequence−specific binding
transcription factor activity, RNA polymerase II core promoter proximal region sequence−specific binding
cytoskeletal adaptor activity
RNA polymerase II core promoter proximal region sequence−specific DNA binding
core promoter proximal region sequence−specific DNA binding
core promoter proximal region DNA binding
actin filament binding

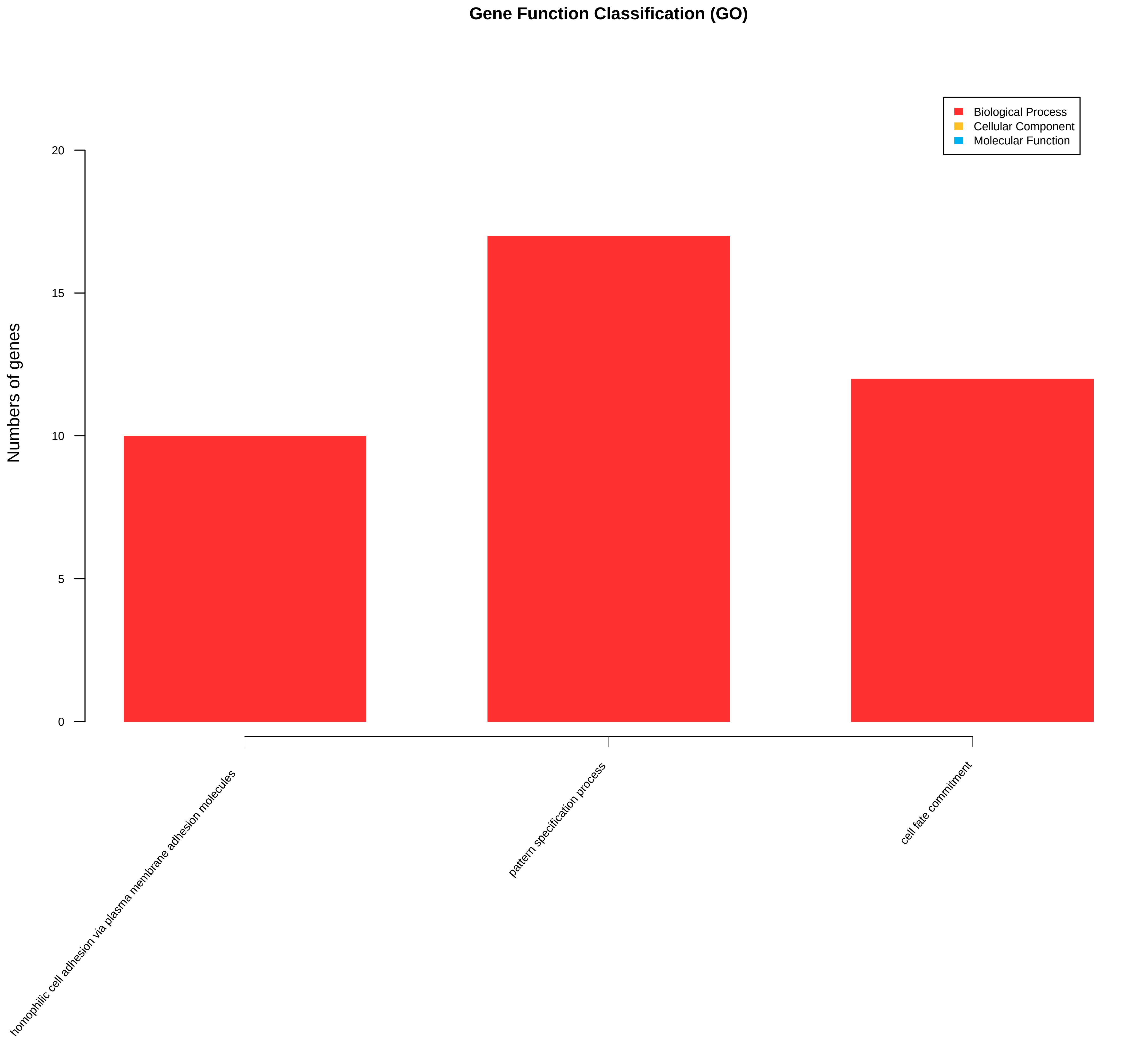
Gene Function Classification (GO)
Biological Process
Cellular Component
Molecular Function
Numbers of genes
20
15
10
5
0
homophilic cell adhesion via plasma membrane adhesion molecules
pattern specification process
cell fate commitment

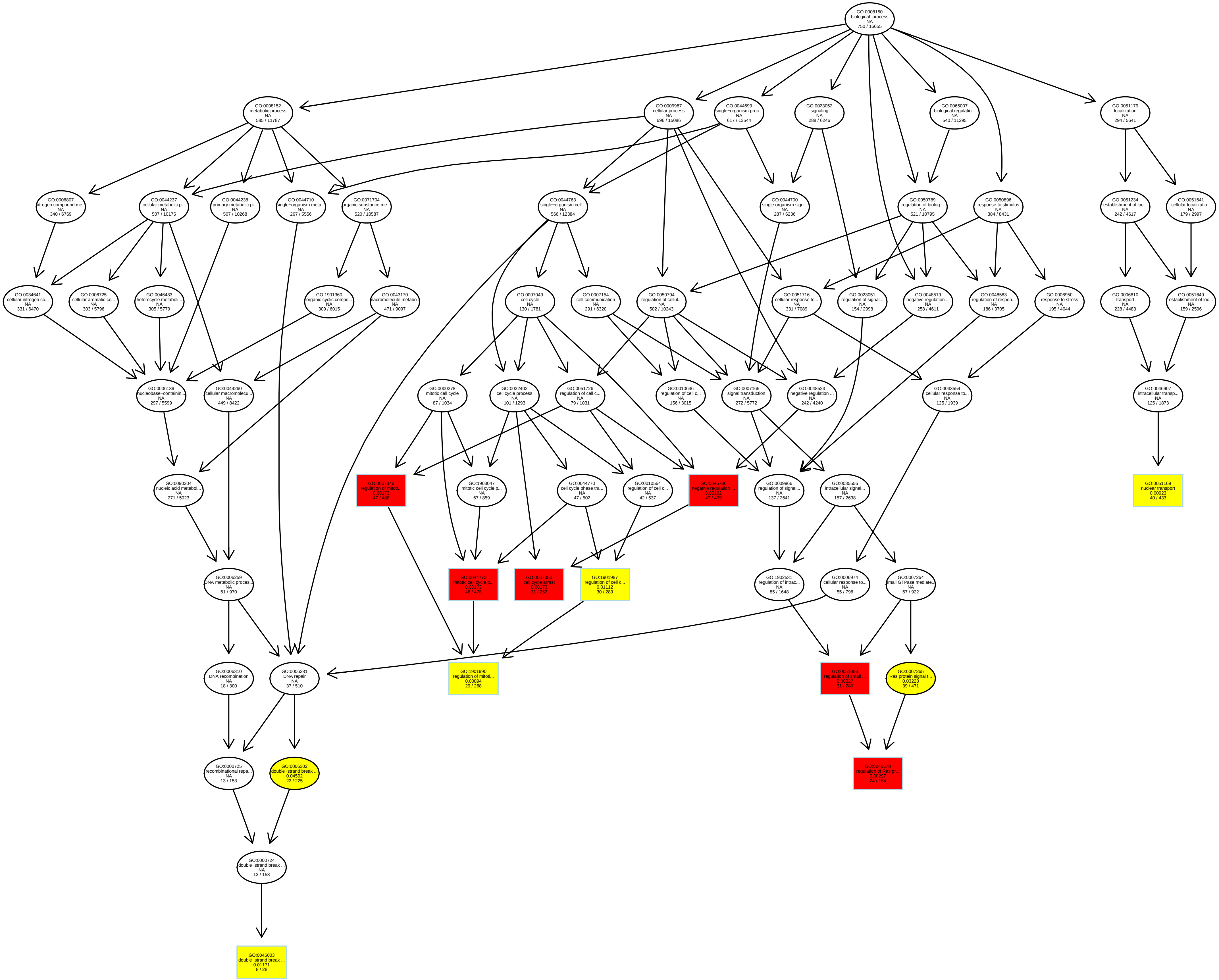

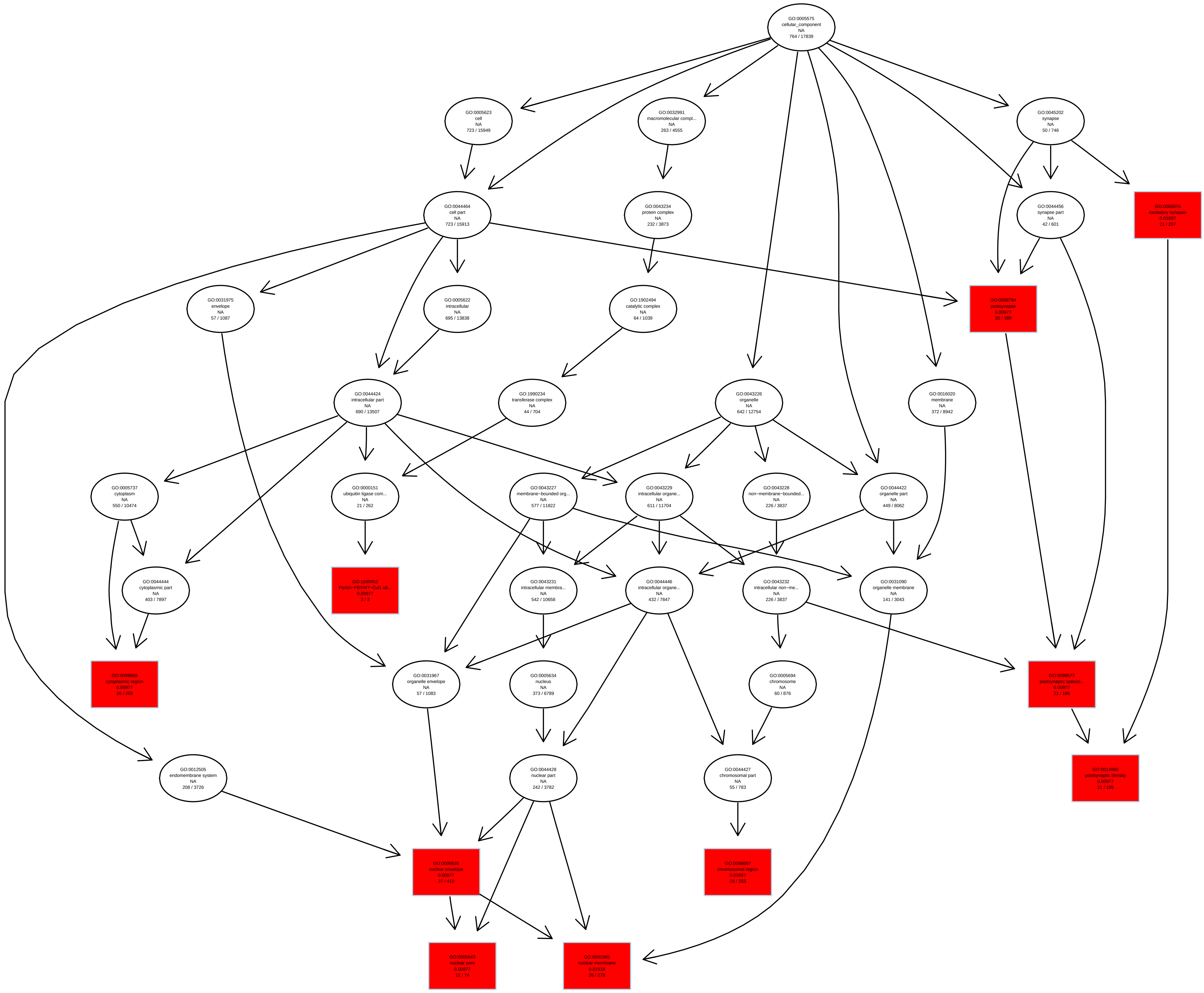
GO:0005575 cellular_component NA 764 / 17839
GO:0005623 cell NA 723 / 15949
GO:0032991 macromolecular compl... NA 263 / 4555
GO:0045202 synapse NA 50 / 746
GO:0044464 cell part NA 723 / 15913
GO:0043234 protein complex NA 232 / 3873
GO:0044456 synapse part NA 42 / 601
GO:0060076 excitatory synapse 0.01697 21 / 207
GO:0031975 envelope NA 57 / 1087
GO:0005622 intracellular NA 695 / 13838
GO:1902494 catalytic complex NA 64 / 1039
GO:0098794 postsynapse 0.00977 35 / 389
GO:0044424 intracellular part NA 690 / 13507
GO:1990234 transferase complex NA 44 / 704
GO:0043226 organelle NA 642 / 12754
GO:0016020 membrane NA 372 / 8942
GO:0005737 cytoplasm NA 550 / 10474
GO:0000151 ubiquitin ligase com... NA 21 / 262
GO:0043227 membrane−bounded org... NA 577 / 11822
GO:0043229 intracellular organe... NA 611 / 11704
GO:0043228 non−membrane−bounded... NA 226 / 3837
GO:0044422 organelle part NA 449 / 8062
GO:0044444 cytoplasmic part NA 403 / 7897
GO:1990452 Parkin−FBXW7−Cul1 ub... 0.00977 3 / 3
GO:0043231 intracellular membra... NA 542 / 10658
GO:0044446 intracellular organe... NA 432 / 7847
GO:0043232 intracellular non−me... NA 226 / 3837
GO:0031090 organelle membrane NA 141 / 3043
GO:0099568 cytoplasmic region 0.00977 26 / 259
GO:0031967 organelle envelope NA 57 / 1083
GO:0005634 nucleus NA 373 / 6789
GO:0005694 chromosome NA 60 / 876
GO:0099572 postsynaptic special... 0.00977 21 / 195
GO:0012505 endomembrane system NA 208 / 3726
GO:0044428 nuclear part NA 242 / 3782
GO:0044427 chromosomal part NA 55 / 783
GO:0014069 postsynaptic density 0.00977 21 / 195
GO:0005635 nuclear envelope 0.00977 37 / 410
GO:0098687 chromosomal region 0.01697 29 / 333
GO:0005643 nuclear pore 0.00977 12 / 74
GO:0031965 nuclear membrane 0.01518 26 / 279

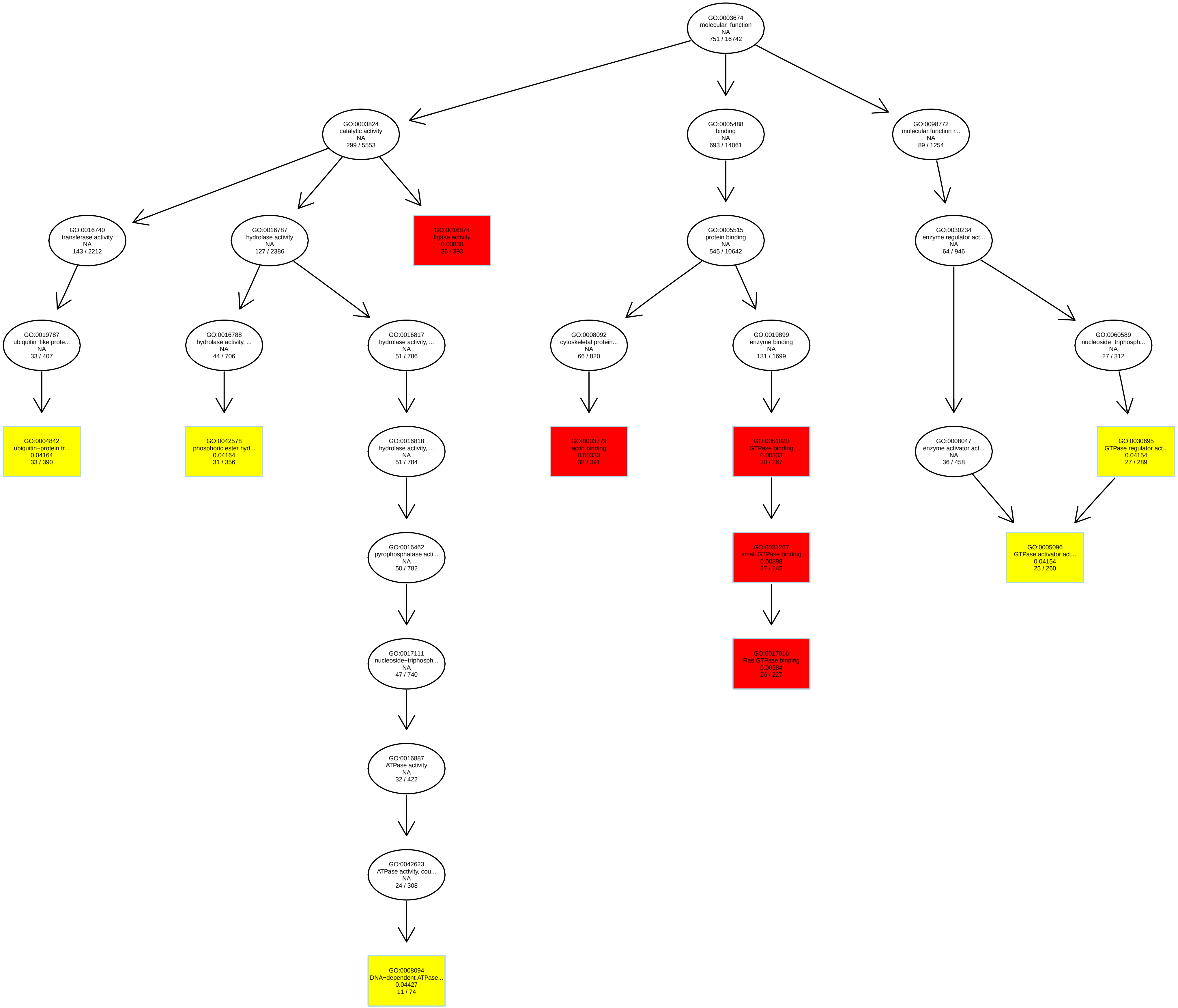
GO:0003674
molecular_function
NA
751 / 16742
GO:0003824
catalytic activity
NA
299 / 5553
GO:0005488
binding
NA
693 / 14061
GO:0098772
molecular function r...
NA
89 / 1254
GO:0016740
transferase activity
NA
143 / 2212
GO:0016787
hydrolase activity
NA
127 / 2386
GO:0016874
ligase activity
0.00830
36 / 393
GO:0005515
protein binding
NA
545 / 10642
GO:0030234
enzyme regulator act...
NA
64 / 946
GO:0019787
ubiquitin−like prote...
NA
33 / 407
GO:0016788
hydrolase activity, ...
NA
44 / 706
GO:0016817
hydrolase activity, ...
NA
51 / 786
GO:0008092
cytoskeletal protein...
NA
66 / 820
GO:0019899
enzyme binding
NA
131 / 1699
GO:0060589
nucleoside−triphosph...
NA
27 / 312
GO:0004842
ubiquitin−protein tr...
0.04164
33 / 390
GO:0042578
phosphoric ester hyd...
0.04164
31 / 356
GO:0016818
hydrolase activity, ...
NA
51 / 784
GO:0003779
actin binding
0.00333
38 / 391
GO:0051020
GTPase binding
0.00333
30 / 267
GO:0008047
enzyme activator act...
NA
36 / 458
GO:0030695
GTPase regulator act...
0.04154
27 / 289
GO:0016462
pyrophosphatase acti...
NA
50 / 782
GO:0031267
small GTPase binding
0.00398
27 / 245
GO:0005096
GTPase activator act...
0.04154
25 / 260
GO:0017111
nucleoside−triphosph...
NA
47 / 740
GO:0017016
Ras GTPase binding
0.00384
26 / 227
GO:0016887
ATPase activity
NA
32 / 422
GO:0042623
ATPase activity, cou...
NA
24 / 308
GO:0008094
DNA−dependent ATPase...
0.04427
11 / 74

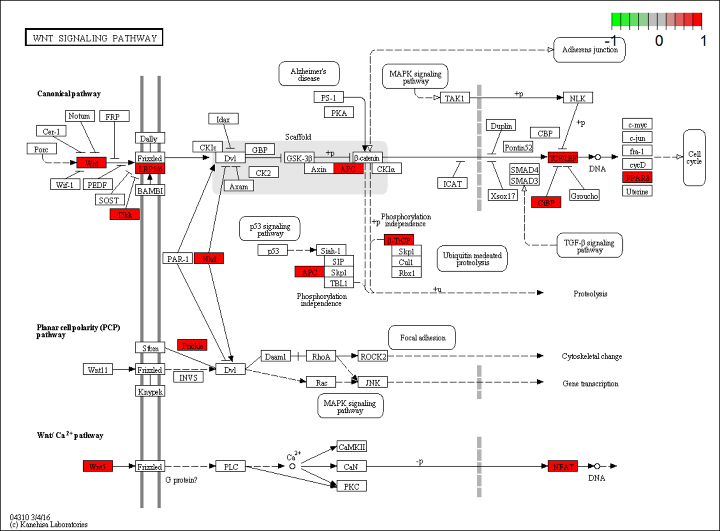
WNT SIGNALING PATHWAY
-1
0
1
Canonical pathway
Notum
FRP
Cer-1
Porc
Wnt
Dally
Frizzled
LRP5/6
Wif-1
PEDF
SOST
BAMBI
Dkk
Idax
CKIε
Dvl
GBP
CK2
Axam
Scaffold
GSK-3β
+p
Axin
APC
β-catenin
CKIα
Alzheimer's disease
PS-1
PKA
MAPK signaling pathway
TAK1
+p
NLK
Adherens junction
Duplin
CBP
Pontin52
+p
TCF/LEF
DNA
SMAD4
SMAD3
ICAT
Xsox17
CtBP
Groucho
c-myc
c-jun
fra-1
cycD
PPARδ
Uterine
Cell cycle
p53 signaling pathway
p53
Siah-1
SIP
APC
Skp1
TBL1
Phosphorylation independence
+p
Phosphorylation independence
β-TrCP
Skp1
Cul1
Rbx1
Ubiquitin mediated proteolysis
+u
Proteolysis
TGF-β signaling pathway
PAR-1
Nkd
Planar cell polarity (PCP) pathway
Stbm
Prickle
Wnt11
Frizzled
INVS
Knypek
Dvl
Daam1
RhoA
ROCK2
Rac
JNK
Focal adhesion
MAPK signaling pathway
Cytoskeletal change
Gene transcription
Wnt/ Ca2+ pathway
Wnt5
Frizzled
G protein?
PLC
Ca2+
CaMKII
CaN
PKC
-p
NFAT
DNA
04310 3/4/16
(c) Kanehisa Laboratories

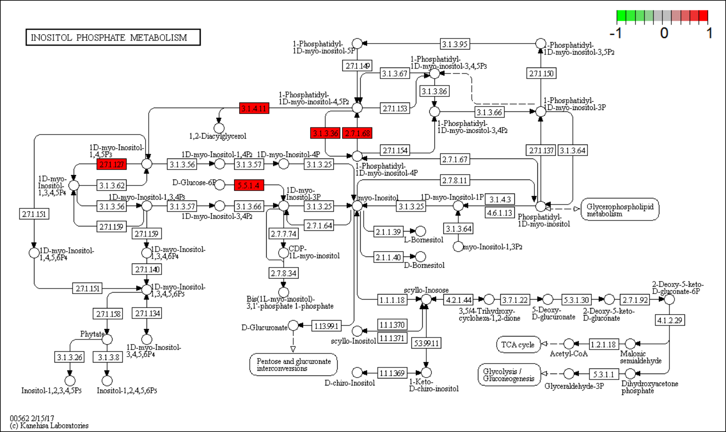

INOSITOL PHOSPHATE METABOLISM
-1
0
1
1-Phosphatidyl-1D-myo-inositol-5P
3.1.3.95
1-Phosphatidyl-1D-myo-inositol-3,5P2
2.7.1.149
3.1.3.67
1-Phosphatidyl-1D-myo-inositol-3,4,5P3
2.7.1.150
3.1.3.86
1-Phosphatidyl-1D-myo-inositol-4,5P2
3.1.4.11
2.7.1.153
3.1.3.66
1-Phosphatidyl-1D-myo-inositol-3P
1,2-Diacylglycerol
1-Phosphatidyl-1D-myo-inositol-3,4P2
3.1.3.36
2.7.1.68
1D-myo-Inositol-1,4,5P3
2.7.1.154
2.7.1.137
3.1.3.64
1D-myo-Inositol-1,4P2
1D-myo-Inositol-4P
2.7.1.67
2.7.1.127
3.1.3.56
3.1.3.57
3.1.3.25
1-Phosphatidyl-1D-myo-inositol-4P
1D-myo-Inositol-1,3,4,5P4
3.1.3.62
D-Glucose-6P
5.5.1.4
2.7.8.11
1D-myo-Inositol-3P
1D-myo-Inositol-1,3,4P3
myo-Inositol
1D-myo-Inositol-1P
3.1.4.3
3.1.3.56
3.1.3.57
3.1.3.66
3.1.3.25
3.1.3.25
4.6.1.13
Phosphatidyl-1D-myo-inositol
Glycerophospholipid metabolism
2.7.1.151
1D-myo-Inositol-3,4P2
2.7.1.159
2.7.1.64
2.7.1.159
2.7.7.74
2.1.1.39
L-Bornesitol
3.1.3.64
CDP-1L-myo-inositol
myo-Inositol-1,3P2
1D-myo-Inositol-1,4,5,6P4
1D-myo-Inositol-1,3,4,6P4
2.1.1.40
D-Bornesitol
2.7.1.140
2.7.8.34
2.7.1.151
1D-myo-Inositol-1,3,4,5,6P5
scyllo-Inosose
2-Deoxy-5-keto-D-gluconate-6P
1.1.1.18
4.2.1.44
3.7.1.22
5.3.1.30
2.7.1.92
Bis(1L-myo-inositol)-3,1'-phosphate 1-phosphate
3,5/4-Trihydroxy-cyclohexa-1,2-dione
5-Deoxy-D-glucuronate
2-Deoxy-5-keto-D-gluconate
2.7.1.158
2.7.1.134
4.1.2.29
1.1.1.370
D-Glucuronate
1.13.99.1
Phytate
scyllo-Inositol
1.1.1.371
TCA cycle
Acetyl-CoA
1.2.1.18
Malonic semialdehyde
53.99.11
1D-myo-Inositol-3,4,5,6P4
3.1.3.26
3.1.3.8
Pentose and glucuronate interconversions
1.1.1.369
Glycolysis / Gluconeogenesis
5.3.1.1
D-chiro-Inositol
1-Keto-D-chiro-inositol
Glyceraldehyde-3P
Dihydroxyacetone phosphate
Inositol-1,2,3,4,5P5
Inositol-1,2,4,5,6P5
00562 2/15/17
(c) Kanehisa Laboratories

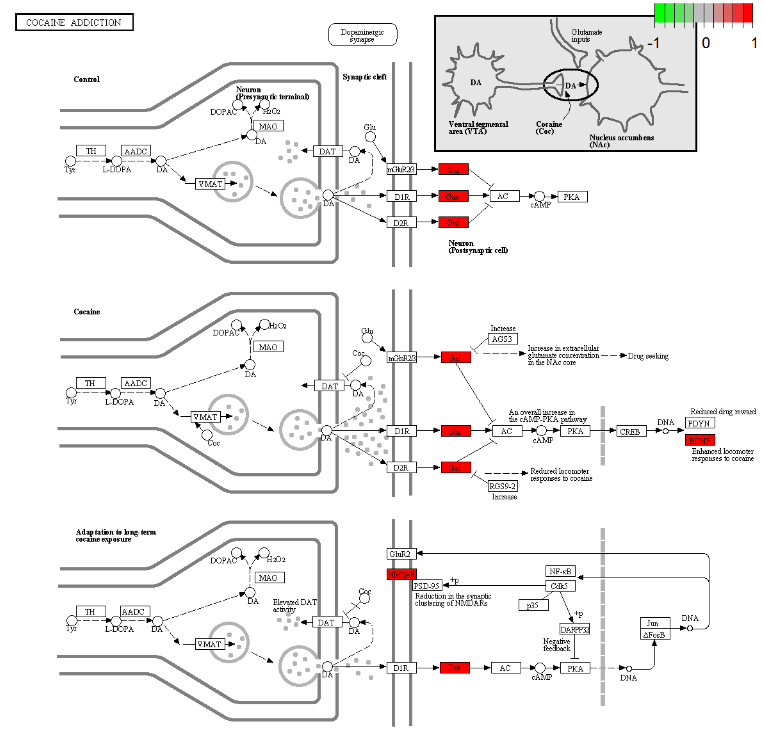

05030 4/26/12

(c) Kanehisa Laboratories

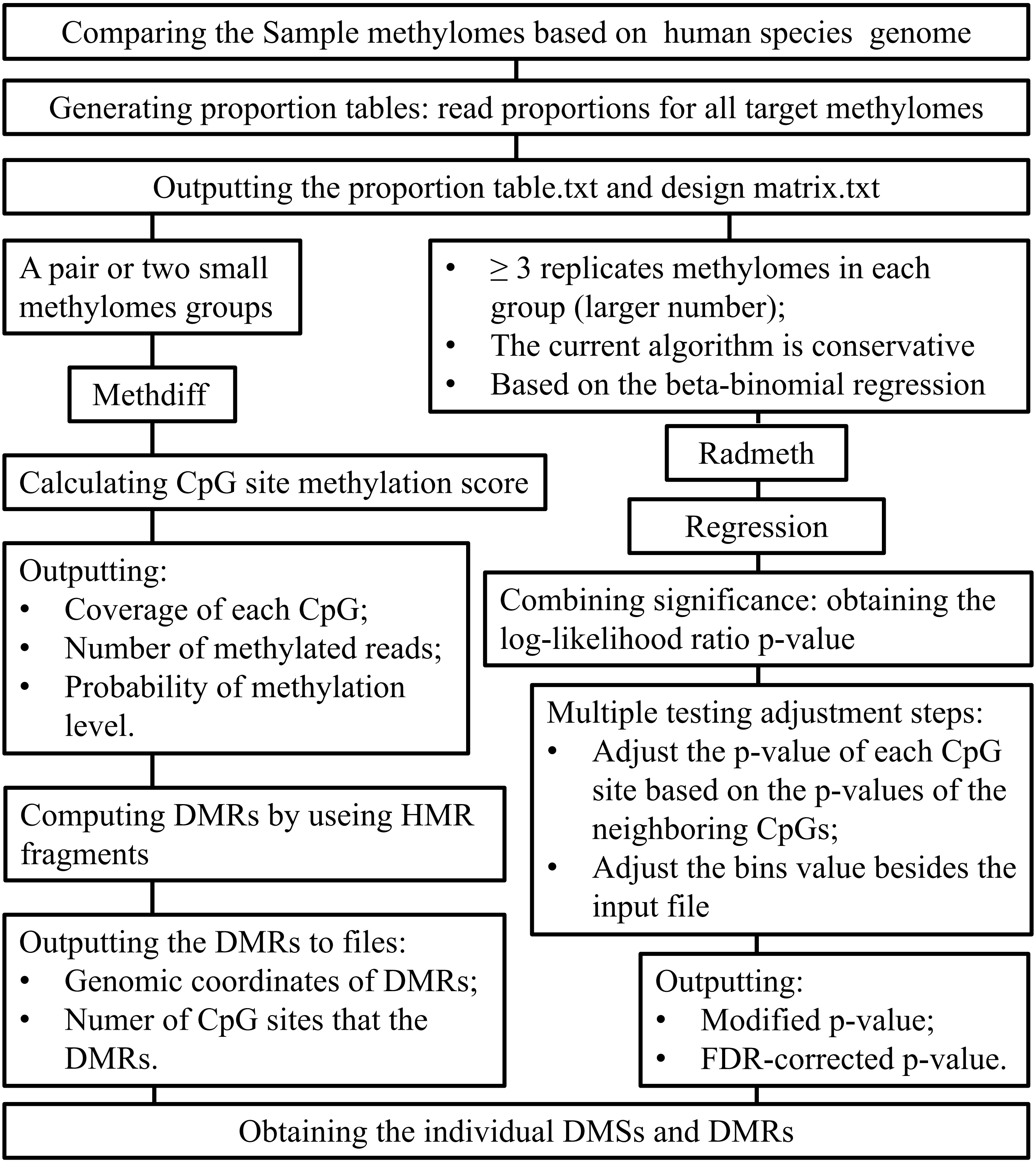
Comparing the Sample methylomes based on human species genome
Generating proportion tables: read proportions for all target methylomes
Outputting the proportion table.txt and design matrix.txt
A pair or two small methylomes groups
Methdiff
Calculating CpG site methylation score
Outputting:
• Coverage of each CpG;
• Number of methylated reads;
• Probability of methylation level.
Computing DMRs by useing HMR fragments
Outputting the DMRs to files:
• Genomic coordinates of DMRs;
• Numer of CpG sites that the DMRs.
• ≥ 3 replicates methylomes in each group (larger number);
• The current algorithm is conservative
• Based on the beta-binomial regression
Radmeth
Regression
Combining significance: obtaining the log-likelihood ratio p-value
Multiple testing adjustment steps:
• Adjust the p-value of each CpG site based on the p-values of the neighboring CpGs;
• Adjust the bins value besides the input file
Outputting:
• Modified p-value;
• FDR-corrected p-value.
Obtaining the individual DMSs and DMRs

Supplementary Table 1： The genes related DMRs between the OST-0D group and the OST-7D group by DiffMeth method analysis

Chr	Start	End	CpG sites	Num-sites	the value of Meth-diff	Gene associated regions
chr1	2344140	2344889	X:76	9	+	PEX10:promoter
chr1	2424829	2425255	X:22	8	+	PLCH2:intron
chr1	3061790	3062910	X:50	7	+	PRDM16:intron
chr1	3164511	3164930	X:18	12	+	PRDM16:intron
chr1	3568495	3568855	X:39	12	+	TP73:promoter
chr1	6305277	6305464	X:20	9	+	HES3:CDS:3
chr1	9713439	9713749	X:11	5	+	C1orf200:ncexon:2
chr1	23066258	23066696	X:17	10	+	EPHB2:intron
chr1	25257599	25257930	X:25	8	+	RUNX3:promoter
chr1	26026292	26027038	X:10	7	+	MAN1C1:intron
chr1	32135441	32135801	X:11	5	+	COL16A1:intron
chr1	39871261	39873833	X:26	5	+	MACF1:intron
chr1	47799412	47799486	X:14	8	+	CMPK1:promoter
chr1	47799412	47799486	X:14	8	+	CMPK1:promoter,CMPK1:5UTR
chr1	52455818	52456010	X:13	5	+	RAB3B:intron
chr1	89457203	89457641	X:18	13	+	RBMXL1:intron,CCBL2:intron
chr1	113051385	113051519	X:21	7	+	WNT2B:promoter
chr1	113051385	113051519	X:21	7	+	WNT2B:promoter,WNT2B:5UTR
chr1	119521629	119522501	X:43	6	+	TBX15:intron
chr1	146895262	146895660	X:12	5	+	LOC100289211:intron,FLJ39739:intron,LOC728855:intron,PPIAL4B:intron,NBPF11:intron,PPIAL4A:intron,GPR89C:intron,NBPF24:intron,PDZK1P1:intron

chr1	149604553	149606699	X:70	5	+	LOC728855:intron,FAM91A2:intron,HIST2H2BF:intron
chr1	156815427	156815904	X:39	10	+	INSRR:CDS:10,INSRR:CDS:9
chr1	165321402	165322172	X:46	11	+	LMX1A:intron
chr1	165323191	165323732	X:36	13	+	LMX1A:intron
chr1	166890456	166890693	X:26	7	+	ILDR2:CDS:9
chr1	170640024	170640754	X:29	5	+	PRRX1:intron
chr1	203829693	203830300	X:23	10	+	SNRPE:promoter
chr1	214476555	214477131	X:15	7	+	SMYD2:intron
chr1	224370938	224371082	X:16	8	+	DEGS1:promoter
chr1	224370938	224371082	X:16	8	+	DEGS1:promoter,DEGS1:5UTR
chr1	226411357	226411539	X:27	5	+	MIXL1:CDS:1
chr1	227922721	227922985	X:32	6	+	SNAP47:promoter
chr1	227922721	227922985	X:32	6	+	SNAP47:promoter,SNAP47:5UTR
chr1	228401244	228401518	X:33	6	+	OBSCN:CDS:2
chr1	235667482	235667755	X:36	7	+	B3GALNT2:promoter
chr1	235667482	235667755	X:36	7	+	B3GALNT2:promoter,B3GALNT2:5UTR
chr1	236686011	236686929	X:23	10	+	LGALS8:promoter
chr1	236686011	236686929	X:23	10	+	LGALS8:promoter,LGALS8:5UTR
chr1	247579508	247580310	X:17	8	+	NLRP3:promoter
chr10	6213830	6214153	X:14	8	+	PFKFB3:intron
chr10	35736721	35737318	X:14	6	+	CCNY:intron
chr10	49879397	49880157	X:37	12	+	FAM21B:intron
chr10	54074079	54074336	X:18	12	+	DKK1:promoter
chr10	54074079	54074336	X:18	12	+	DKK1:promoter,DKK1:5UTR

chr10	74094093	74094869	X:26	5	+	DNAJB12:3UTR
chr10	78011602	78012721	X:15	5	+	C10orf11:intron
chr10	102586628	102586778	X:16	9	+	PAX2:CDS:10
chr10	103534171	103534715	X:19	6	+	FGF8:promoter
chr10	103534171	103534715	X:19	6	+	FGF8:promoter,FGF8:5UTR
chr10	121578172	121578402	X:28	11	+	INPP5F:promoter
chr10	125447737	125450617	X:19	6	+	GPR26:3UTR
chr10	126840910	126841148	X:16	6	+	CTBP2:intron
chr10	134980773	134980953	X:17	5	+	KNDC1:CDS:2
chr11	1948028	1949013	X:29	7	+	TNNT3:intron
chr11	2159301	2159451	X:21	6	+	IGF2:intron,INS-IGF2:intron
chr11	2720829	2721027	X:29	7	+	KCNQ1OT1:ncexon:1
chr11	13032404	13032746	X:20	8	+	RASSF10:CDS:1
chr11	13689903	13690088	X:19	15	+	FAR1:promoter
chr11	27722013	27722332	X:38	6	+	BDNF:promoter
chr11	27722013	27722332	X:38	6	+	BDNF:promoter,BDNF:5UTR,BDNF:5UTR
chr11	64120582	64120895	X:17	6	+	CCDC88B:CDS:21,CCDC88B:CDS:22
chr11	66193851	66194008	X:13	6	+	NPAS4:3UTR
chr11	69518419	69518639	X:26	6	+	FGF19:CDS:1
chr12	3345678	3346257	X:14	5	+	TSPAN9:intron
chr12	42983100	42983268	X:15	11	+	PRICKLE1:5UTR
chr12	49208212	49208388	X:15	8	+	CACNB3:promoter,CACNB3:promoter
chr12	49208212	49208388	X:15	8	+	CACNB3:promoter,CACNB3:promoter,CACNB3:5UTR
chr12	58239441	58239680	X:23	5	+	CTDSP2:intron

chr12	94580367	94580738	X:19	5	+	PLXNC1:intron
chr12	115109568	115109701	X:12	6	+	TBX3:CDS:7
chr12	115112443	115112608	X:18	6	+	TBX3:CDS:6
chr12	120105873	120106085	X:19	7	+	PRKAB1:5UTR
chr12	124242063	124242821	X:10	7	+	ATP6V0A2:CDS:20
chr12	124873802	124874003	X:17	10	+	NCOR2:intron
chr12	132628607	132628711	X:19	5	+	NOC4L:promoter
chr13	78272441	78272829	X:44	6	+	SLAIN1:promoter
chr13	109806752	109808000	X:20	8	+	MYO16:intron
chr13	110953975	110954437	X:13	6	+	COL4A1:intron
chr13	114565770	114565961	X:17	7	+	GAS6:intron
chr14	24780256	24780463	X:24	13	+	LTB4R:promoter
chr14	24780256	24780463	X:24	13	+	LTB4R:promoter,CIDEB:5UTR
chr14	55139594	55140493	X:11	5	+	SAMD4A:intron
chr14	74222353	74223554	X:18	5	+	C14orf43:intron
chr14	97263704	97264018	X:51	15	+	VRK1:promoter
chr14	97263704	97264018	X:51	15	+	VRK1:promoter,VRK1:5UTR
chr14	102430943	102431130	X:24	7	+	DYNC1H1:promoter
chr14	102430943	102431130	X:24	7	+	DYNC1H1:promoter,DYNC1H1:5UTR
chr15	20710460	20711337	X:42	5	+	HERC2P3:ncexon:2
chr15	25200003	25200775	X:54	13	+	SNRPN:promoter,SNURF:promoter
chr15	25200003	25200775	X:54	13	+	SNRPN:promoter,SNURF:promoter,SNRPN:5UTR,SNURF:5UTR
chr15	40399722	40400614	X:18	5	+	BMF:promoter
chr15	40399722	40400614	X:18	5	+	BMF:promoter,BMF:5UTR

chr15	41061478	41062200	X:22	5	+	C15orf62:promoter
chr15	41061478	41062200	X:22	5	+	C15orf62:promoter,C15orf62:5UTR
chr15	41787761	41788038	X:25	5	+	ITPKA:intron
chr15	45315202	45315418	X:16	9	+	SORD:promoter
chr15	45315202	45315418	X:16	9	+	SORD:promoter,SORD:5UTR
chr15	67053256	67054632	X:28	6	+	SMAD6:intron
chr15	74427108	74427865	X:40	9	+	ISLR2:CDS:1
chr15	76634317	76634559	X:14	12	+	ISL2:3UTR
chr15	86314770	86315413	X:12	5	+	KLHL25:intron
chr15	100273533	100273946	X:44	9	+	LYSMD4:promoter
chr15	100273533	100273946	X:44	9	+	LYSMD4:promoter,LYSMD4:5UTR
chr16	216315	216625	X:32	17	+	HBM:CDS:2,HBM:CDS:3
chr16	433504	434082	X:25	5	+	LOC100134368:intron
chr16	672297	672576	X:25	10	+	RAB40C:intron
chr16	1204446	1204878	X:21	6	+	CACNA1H:intron
chr16	2097589	2097729	X:13	11	+	TSC2:promoter
chr16	3304362	3304625	X:24	6	+	MEFV:CDS:2
chr16	24863072	24863842	X:14	6	+	SLC5A11:intron
chr16	27829315	27829980	X:19	6	+	GSG1L:intron
chr16	28936431	28936492	X:11	9	+	RABEP2:promoter
chr16	28936431	28936492	X:11	9	+	RABEP2:promoter,RABEP2:5UTR
chr16	55364261	55365019	X:30	11	+	IRX6:3UTR
chr16	66613134	66613355	X:32	7	+	CMTM2:promoter
chr16	66613134	66613355	X:32	7	+	CMTM2:promoter,CMTM2:5UTR

chr16	78537144	78538574	X:11	5	+	WWOX:intron
chr16	85648027	85648263	X:16	5	+	KIAA0182:intron
chr16	86612451	86612589	X:13	5	+	FOXL1:CDS:1
chr16	88540162	88540349	X:14	5	+	ZFPM1:intron
chr17	1969792	1970573	X:18	5	+	SMG6:intron
chr17	6347681	6347790	X:11	8	+	FAM64A:promoter
chr17	6347681	6347790	X:11	8	+	FAM64A:promoter,FAM64A:5UTR
chr17	7196058	7197162	X:18	6	+	YBX2:CDS:2
chr17	11900526	11900821	X:37	10	+	ZNF18:promoter
chr17	11900526	11900821	X:37	10	+	ZNF18:promoter,ZNF18:5UTR
chr17	18585445	18585650	X:17	10	+	ZNF286B:promoter
chr17	18585445	18585650	X:17	10	+	ZNF286B:promoter,ZNF286B:5UTR
chr17	37380676	37381143	X:37	21	+	STAC2:intron
chr17	59490308	59490809	X:26	6	+	C17orf82:ncexon:1
chr17	77395836	77396488	X:24	7	+	RBFOX3:intron
chr17	79093142	79093348	X:26	9	+	AATK:CDS:11
chr17	79670077	79670280	X:30	12	+	MRPL12:promoter
chr17	80290051	80290226	X:13	6	+	SECTM1:intron
chr17	80605959	80606237	X:31	5	+	WDR45L:5UTR
chr18	20715369	20715469	X:14	5	+	CABLES1:intron
chr18	21083110	21083275	X:15	12	+	C18orf8:promoter
chr19	1009580	1009797	X:14	5	+	GRIN3B:CDS:9
chr19	1465410	1465639	X:26	5	+	APC2:CDS:14
chr19	2241107	2241325	X:15	5	+	SF3A2:intron

chr19	3668289	3668603	X:14	6	+	PIP5K1C:intron
chr19	4368257	4369372	X:32	6	+	SH3GL1:intron
chr19	4976449	4976931	X:21	9	+	KDM4B:intron
chr19	5340953	5341042	X:14	9	+	PTPRS:promoter
chr19	6750259	6750505	X:15	6	+	TRIP10:CDS:12
chr19	12983791	12984483	X:45	10	+	MAST1:CDS:25,MAST1:CDS:26
chr19	14260390	14260774	X:19	6	+	LPHN1:3UTR
chr19	18548703	18548888	X:21	6	+	ISYNA1:promoter
chr19	18548703	18548888	X:21	6	+	ISYNA1:promoter,ISYNA1:5UTR
chr19	30866169	30866489	X:38	11	+	ZNF536:intron
chr19	34113283	34113373	X:14	5	+	CHST8:intron
chr19	40366340	40366575	X:26	6	+	FCGBP:CDS:30
chr19	40421120	40421435	X:32	8	+	FCGBP:CDS:5
chr19	46196061	46196194	X:11	8	+	SNRPD2:promoter
chr19	48946126	48946668	X:93	7	+	GRIN2D:CDS:12
chr19	49561304	49561963	X:26	7	+	SNAR-A10:intron,SNAR-A7:intron,SNAR-A3:intron,SNAR-A4:intron,SNAR-A5:intron,SNAR-A14 :intron,SNAR-A8:intron,SNAR-A6:intron,SNAR-A11:intron,SNAR-A9:intron
chr19	49935752	49935999	X:23	6	+	SLC17A7:CDS:9
chr19	51486749	51487309	X:16	7	+	KLK7:promoter
chr19	51486749	51487309	X:16	7	+	KLK7:promoter,KLK7:5UTR,KLK7:5UTR
chr19	55763066	55763326	X:11	6	+	PPP6R1:intron
chr19	58545285	58545420	X:17	8	+	ZSCAN1:promoter
chr19	58740222	58740358	X:16	9	+	ZNF544:promoter
chr19	58740222	58740358	X:16	9	+	ZNF544:promoter,ZNF544:5UTR

chr19	58867468	58867766	X:36	6	+	ZNF497:CDS:1
chr19	58879211	58879349	X:18	7	+	ZNF837:CDS:1
chr2	66672616	66673226	X:42	12	+	MEIS1:intron
chr2	74729802	74729915	X:10	5	+	LBX2:CDS:1
chr2	74781352	74781451	X:12	5	+	DOK1:promoter,LOXL3:promoter
chr2	88124677	88125044	X:42	13	+	RGPD2:intron,RGPD1:intron
chr2	91844804	91845379	X:21	5	+	LOC654342:intron
chr2	95787634	95787789	X:20	13	+	MRPS5:promoter
chr2	95787634	95787789	X:20	13	+	MRPS5:promoter,MRPS5:5UTR
chr2	120189353	120189544	X:27	5	+	TMEM37:promoter
chr2	120189353	120189544	X:27	5	+	TMEM37:promoter,TMEM37:5UTR
chr2	130691761	130691931	X:17	12	+	LOC389033:ncexon:1
chr2	133014647	133014966	X:26	6	+	MIR663B:ncexon:1
chr2	171570943	171571199	X:26	6	+	SP5:promoter
chr2	177029537	177029757	X:21	6	+	HOXD3:intron
chr2	177036436	177036608	X:18	5	+	HOXD3:CDS:2
chr2	192542655	192542828	X:18	12	+	OBFC2A:promoter
chr2	192542655	192542828	X:18	12	+	OBFC2A:promoter,OBFC2A:5UTR
chr2	200330906	200332521	X:33	10	+	SATB2:intron
chr2	223917511	223917734	X:13	6	+	KCNE4:promoter
chr2	223917511	223917734	X:13	6	+	KCNE4:promoter,KCNE4:5UTR
chr2	228337096	228337435	X:39	10	+	AGFG1:5UTR
chr20	15897688	15898740	X:10	5	+	MACROD2:intron
chr20	21685400	21685841	X:20	8	+	PAX1:promoter

chr20	30135112	30135362	X:21	6	+	PSIMCT-1:ncexon:1
chr20	37434950	37435127	X:18	8	+	PPP1R16B:intron
chr20	57465119	57465388	X:31	6	+	GNAS:intron
chr20	57766930	57767281	X:39	5	+	ZNF831:CDS:1
chr20	62120430	62120605	X:16	5	+	EEF1A2:CDS:6
chr21	15588309	15588491	X:20	6	+	RBM11:promoter
chr21	15588309	15588491	X:20	6	+	RBM11:promoter,RBM11:5UTR
chr21	34443296	34443508	X:17	7	+	OLIG1:CDS:1
chr21	43812801	43813768	X:23	6	+	TMPRSS3:intron
chr21	45564710	45565141	X:16	5	+	C21orf33:CDS:6
chr21	46826425	46827096	X:27	6	+	COL18A1:intron
chr22	18064009	18064263	X:14	5	+	SLC25A18:CDS:3
chr22	36766602	36767424	X:15	6	+	MYH9:intron
chr22	37261500	37261766	X:13	6	+	NCF4:intron
chr22	42084782	42084933	X:16	16	+	NHP2L1:promoter
chr22	42084782	42084933	X:16	16	+	NHP2L1:promoter,NHP2L1:5UTR
chr22	48948274	48948708	X:16	5	+	FAM19A5:intron
chr3	28390613	28390932	X:24	9	+	AZI2:promoter
chr3	28390613	28390932	X:24	9	+	AZI2:promoter,AZI2:5UTR
chr3	45837675	45837847	X:18	10	+	SLC6A20:CDS:1
chr3	46975614	46977038	X:16	6	+	CCDC12:intron
chr3	47844666	47844836	X:14	8	+	DHX30:5UTR
chr3	50273544	50273744	X:26	9	+	GNAI2:promoter
chr3	50273544	50273744	X:26	9	+	GNAI2:promoter,GNAI2:5UTR

chr3	129305777	129306109	X:13	5	+	PLXND1:intron
chr3	133502608	133502725	X:10	5	+	SRPRB:promoter
chr3	147122696	147123473	X:22	8	+	ZIC4:promoter,ZIC4:promoter
chr3	147122696	147123473	X:22	8	+	ZIC4:promoter,ZIC4:promoter,ZIC4:5UTR
chr4	1188632	1188760	X:15	11	+	SPON2:intron
chr4	13529671	13530416	X:29	12	+	LOC285547:ncexon:3
chr4	13542995	13543676	X:23	6	+	NKX3-2:CDS:2
chr4	57458843	57458974	X:17	6	+	THEGL:intron
chr4	81110244	81110530	X:20	5	+	PRDM8:intron
chr4	166033652	166033878	X:22	6	+	TMEM192:intron
chr4	187071281	187071868	X:28	5	+	FAM149A:intron
chr4	187124575	187124894	X:10	7	+	CYP4V2:intron
chr5	2748664	2748828	X:21	9	+	IRX2:CDS:3
chr5	10649619	10650054	X:51	9	+	ANKRD33B:CDS:4
chr5	31937105	31937829	X:12	5	+	PDZD2:intron
chr5	37249426	37249598	X:23	18	+	C5orf42:promoter
chr5	37249426	37249598	X:23	18	+	C5orf42:promoter,C5orf42:5UTR
chr5	77944712	77945044	X:43	13	+	LHFPL2:promoter
chr5	92923024	92923551	X:16	5	+	NR2F1:intron
chr5	92924343	92926913	X:57	11	+	NR2F1:intron
chr5	112257909	112258099	X:30	9	+	REEP5:promoter
chr5	112257909	112258099	X:30	9	+	REEP5:promoter,REEP5:5UTR
chr5	122434853	122435523	X:47	7	+	PRDM6:CDS:2
chr5	133513400	133513712	X:14	6	+	SKP1:promoter

chr5	135416032	135416725	X:34	5	+	VTRNA2-1:ncexon:1
chr5	140242203	140242414	X:24	6	+	PCDHA9:intron,PCDHA6:intron,PCDHA8:intron,PCDHA5:intron,PCDHA2:intron,PCDHA4:intron, PCDHA10:intron,PCDHA1:intron,PCDHA7:intron,PCDHA3:intron
chr5	159688055	159688772	X:19	5	+	CCNJL:intron
chr5	171433660	171433781	X:11	5	+	FBXW11:promoter
chr5	171433660	171433781	X:11	5	+	FBXW11:promoter,FBXW11:5UTR
chr5	171614782	171614925	X:16	9	+	STK10:CDS:1
chr5	180632027	180632401	X:42	24	+	TRIM7:promoter
chr5	180632027	180632401	X:42	24	+	TRIM7:promoter,TRIM7:5UTR
chr6	12291677	12292901	X:18	6	+	EDN1:CDS:2
chr6	18122832	18123164	X:28	11	+	NHLRC1:promoter
chr6	18122832	18123164	X:28	11	+	NHLRC1:promoter,NHLRC1:5UTR
chr6	24495219	24495442	X:30	7	+	ALDH5A1:promoter
chr6	24495219	24495442	X:30	7	+	ALDH5A1:promoter,ALDH5A1:5UTR
chr6	27441662	27441892	X:13	9	+	ZNF184:promoter
chr6	34113719	34114207	X:18	5	+	GRM4:promoter
chr6	34113719	34114207	X:18	5	+	GRM4:promoter,GRM4:5UTR
chr6	74008935	74009455	X:41	6	+	KHDC1:intron,C6orf147:intron
chr6	158438166	158438463	X:14	5	+	SYNJ2:promoter
chr6	158438166	158438463	X:14	5	+	SYNJ2:promoter,SYNJ2:5UTR
chr6	159465764	159466690	X:18	6	+	TAGAP:promoter
chr6	159465764	159466690	X:18	6	+	TAGAP:promoter,TAGAP:5UTR
chr6	160427262	160427616	X:23	13	+	IGF2R:intron
chr7	1094657	1094970	X:20	7	+	C7orf50:intron

chr7	1272905	1273301	X:42	14	+	UNCX:CDS:1,UNCX:CDS:2
chr7	4831752	4832660	X:72	5	+	KIAA0415:3UTR
chr7	5569549	5569825	X:43	5	+	ACTB:promoter
chr7	5821071	5821244	X:20	15	+	RNF216:5UTR
chr7	27206427	27206711	X:14	6	+	HOXA10-HOXA9:intron
chr7	40174597	40174734	X:14	6	+	C7orf11:promoter,C7orf10:promoter
chr7	40174597	40174734	X:14	6	+	C7orf11:promoter,C7orf10:promoter,C7orf10:5UTR
chr7	66532130	66532662	X:10	5	+	TYW1:CDS:10
chr7	87257532	87257703	X:21	5	+	RUNDC3B:promoter
chr7	94023618	94023924	X:18	5	+	COL1A2:promoter
chr7	94023618	94023924	X:18	5	+	COL1A2:promoter,COL1A2:5UTR
chr7	96631679	96632140	X:20	9	+	DLX6-AS1:intron
chr7	99036585	99036945	X:38	13	+	CPSF4:promoter,PTCD1:promoter
chr7	99036585	99036945	X:38	13	+	CPSF4:promoter,PTCD1:promoter,CPSF4:5UTR
chr7	100203398	100203599	X:18	6	+	PCOLCE:CDS:5
chr7	100823267	100823455	X:16	6	+	NAT16:5UTR
chr7	101049158	101049511	X:15	6	+	EMID2:intron
chr7	127225542	127225723	X:18	10	+	GCC1:promoter
chr7	127225542	127225723	X:18	10	+	GCC1:promoter,GCC1:5UTR
chr7	130130739	130130995	X:26	11	+	MEST:promoter
chr7	145945389	145946093	X:22	5	+	CNTNAP2:intron
chr7	157406375	157406737	X:30	6	+	PTPRN2:intron
chr7	157576860	157577667	X:29	5	+	PTPRN2:intron
chr7	157668518	157669348	X:21	6	+	PTPRN2:intron

chr8	1496930	1497185	X:35	22	+	DLGAP2:CDS:1
chr8	1950836	1951059	X:32	6	+	KBTBD11:CDS:1
chr8	8750331	8750534	X:22	9	+	MFHAS1:CDS:1
chr8	11272335	11272886	X:10	5	+	C8orf12:intron
chr8	22635193	22635576	X:13	6	+	PEBP4:intron
chr8	25896195	25897234	X:25	5	+	EBF2:intron
chr9	34637478	34637802	X:36	8	+	SIGMAR1:promoter
chr9	34637478	34637802	X:36	8	+	SIGMAR1:promoter,SIGMAR1:5UTR
chr9	90112861	90113035	X:18	5	+	DAPK1:promoter
chr9	90112861	90113035	X:18	5	+	DAPK1:promoter,DAPK1:5UTR
chr9	123476699	123476903	X:29	9	+	MEGF9:promoter
chr9	123476699	123476903	X:29	9	+	MEGF9:promoter,MEGF9:5UTR
chr9	126692428	126692758	X:30	9	+	DENND1A:promoter
chr9	130684061	130684177	X:15	15	+	PIP5KL1:CDS:4
chr9	131012953	131013323	X:36	8	+	DNM1:CDS:20
chr9	131939245	131939446	X:22	5	+	IER5L:CDS:1
chr9	133320154	133320631	X:48	10	+	ASS1:promoter
chr9	133320154	133320631	X:48	10	+	ASS1:promoter,ASS1:5UTR
chr9	138078062	138079193	X:30	5	+	LOC401557:ncexon:3
chrX	16737358	16737799	X:28	12	+	SYAP1:promoter
chrX	16737358	16737799	X:28	12	+	SYAP1:promoter,SYAP1:5UTR
chrX	19362105	19362392	X:29	5	+	PDHA1:promoter
chrX	19362105	19362392	X:29	5	+	PDHA1:promoter,PDHA1:5UTR
chrX	21392042	21392377	X:36	5	+	CNKSR2:promoter

chrX	100603685	100603839	X:19	11	+	TIMM8A:promoter
chrX	100603685	100603839	X:19	11	+	TIMM8A:promoter,TIMM8A:5UTR
chrX	153043745	153044220	X:19	6	+	PLXNB3:CDS:32,PLXNB3:CDS:33,PLXNB3:CDS:34
chr1	3154748	3155412	X:30	7	+	PRDM16:intron
chr1	6531040	6531196	X:16	5	+	PLEKHG5:CDS:12
chr1	17092499	17092748	X:10	7	+	MIR3675:intron
chr1	21864787	21864977	X:14	5	+	ALPL:intron
chr1	23720292	23720842	X:16	6	+	TCEA3:CDS:8
chr1	25894682	25895381	X:22	5	+	LDLRAP1:3UTR
chr1	61914545	61915001	X:11	6	+	NFIA:intron
chr1	92216106	92217483	X:11	6	+	TGFBR3:intron
chr1	160054294	160054373	X:13	5	+	KCNJ9:CDS:1
chr1	174843541	174844010	X:10	6	+	RABGAP1L:promoter,RABGAP1L:promoter
chr1	174843541	174844010	X:10	6	+	RABGAP1L:promoter,RABGAP1L:promoter,RABGAP1L:5UTR
chr1	198901566	198903300	X:12	5	+	LOC100131234:intron
chr1	204653481	204653718	X:11	5	+	LRRN2:intron
chr1	245462133	245462493	X:13	7	+	KIF26B:intron
chr10	21144399	21145514	X:10	5	+	NEBL:intron
chr10	24416323	24417665	X:13	5	+	KIAA1217:intron
chr10	30316299	30316709	X:16	9	+	KIAA1462:CDS:2
chr10	85955263	85956268	X:23	7	+	CDHR1:CDS:2,CDHR1:CDS:3
chr10	114712357	114712663	X:19	6	+	TCF7L2:intron
chr10	124907550	124907762	X:26	7	+	HMX2:promoter
chr10	124907550	124907762	X:26	7	+	HMX2:promoter,HMX2:5UTR

chr10	131749397	131749973	X:20	6	+	EBF3:intron
chr11	17786117	17786797	X:21	6	+	KCNC1:intron
chr11	44325258	44325886	X:33	12	+	ALX4:intron
chr11	46384634	46385376	X:25	8	+	DGKZ:intron
chr11	46401392	46401701	X:17	5	+	MDK:promoter
chr11	61481086	61481638	X:14	5	+	DAGLA:intron
chr11	66062246	66062366	X:20	6	+	TMEM151A:CDS:2
chr11	68147850	68148079	X:17	6	+	LRP5:intron
chr11	101783582	101785113	X:10	5	+	KIAA1377:promoter
chr11	122597619	122598238	X:14	6	+	UBASH3B:intron
chr12	2339324	2339614	X:30	9	+	CACNA1C:intron
chr12	49943271	49943434	X:17	5	+	KCNH3:CDS:9
chr12	94659159	94659830	X:11	5	+	PLXNC1:intron
chr12	115107577	115109504	X:28	7	+	TBX3:3UTR
chr12	116482142	116483522	X:10	5	+	MED13L:intron
chr12	124767826	124768330	X:12	5	+	ZNF664-FAM101A:intron
chr13	30077279	30077552	X:22	8	+	MTUS2:CDS:14
chr13	33896239	33896912	X:13	7	+	STARD13:intron
chr13	36642214	36644493	X:14	5	+	DCLK1:intron
chr13	92050752	92050960	X:30	6	+	GPC5:promoter
chr13	92050752	92050960	X:30	6	+	GPC5:promoter,GPC5:5UTR
chr13	101187793	101188225	X:12	6	+	A2LD1:intron
chr13	111058009	111058695	X:12	5	+	COL4A2:intron
chr13	114076067	114076719	X:19	5	+	ADPRHL1:3UTR

chr14	24109492	24110335	X:10	6	+	DHRS2:intron
chr14	56683528	56684516	X:11	6	+	PELI2:intron
chr14	62214248	62217116	X:11	5	+	HIF1A:3UTR
chr14	72467523	72468515	X:13	8	+	RGS6:intron
chr14	98405047	98408445	X:23	5	+	C14orf64:intron
chr15	23892844	23893075	X:10	6	+	MAGEL2:promoter
chr15	23892844	23893075	X:10	6	+	MAGEL2:promoter,MAGEL2:5UTR
chr15	57510119	57511991	X:14	5	+	TCF12:promoter
chr15	57510119	57511991	X:14	5	+	TCF12:promoter,TCF12:5UTR
chr15	58357369	58357534	X:15	7	+	ALDH1A2:intron
chr15	69222761	69222903	X:16	7	+	SPESP1:promoter,NOX5:promoter
chr15	69222761	69222903	X:16	7	+	SPESP1:promoter,NOX5:promoter,SPESP1:5UTR,NOX5:5UTR
chr15	71999914	72001373	X:17	5	+	THSD4:intron
chr15	84505535	84506987	X:13	7	+	ADAMTSL3:CDS:6
chr15	89935336	89938133	X:16	5	+	LOC254559:intron
chr15	92692240	92692896	X:12	5	+	SLCO3A1:intron
chr15	100674236	100675270	X:14	6	+	ADAMTS17:intron
chr15	101729470	101729708	X:10	7	+	CHSY1:intron
chr16	4290662	4290972	X:11	5	+	SRL:intron
chr16	9914611	9916143	X:13	6	+	GRIN2A:CDS:9
chr16	11654526	11655300	X:21	6	+	LITAF:intron
chr16	50589796	50590368	X:18	8	+	NKD1:intron
chr16	78427350	78428596	X:11	6	+	WWOX:intron
chr16	84336085	84336417	X:13	6	+	WFDC1:intron

chr16	86547739	86548894	X:16	5	+	FOXF1:3UTR
chr16	88747897	88748067	X:13	6	+	SNAI3:CDS:2
chr16	89411466	89412009	X:17	5	+	ANKRD11:intron
chr17	1084457	1084956	X:14	6	+	ABR:intron
chr17	30953875	30954650	X:14	5	+	MYO1D:intron
chr17	31810160	31811046	X:13	5	+	ACCN1:intron
chr17	39728143	39728454	X:15	5	+	KRT9:promoter
chr17	39728143	39728454	X:15	5	+	KRT9:promoter,KRT9:5UTR
chr17	43323892	43324256	X:10	6	+	FMNL1:CDS:26
chr17	46628046	46628360	X:22	5	+	HOXB3:CDS:2
chr17	46667304	46667949	X:20	7	+	HOXB-AS3:ncexon:1
chr17	58164409	58165865	X:13	6	+	LOC645638:ncexon:1
chr17	66596122	66596436	X:24	10	+	FAM20A:5UTR
chr17	75318260	75318786	X:17	5	+	SEPT9:intron
chr17	78560453	78560825	X:20	11	+	RPTOR:intron
chr17	78894313	78894844	X:21	9	+	RPTOR:intron
chr17	79011390	79011661	X:12	6	+	BAIAP2:intron
chr17	80805852	80806603	X:28	7	+	TBCD:intron
chr18	447007	449695	X:21	5	+	COLEC12:intron
chr18	46895113	46895480	X:10	6	+	DYM:intron
chr18	55810522	55811072	X:15	5	+	NEDD4L:intron
chr18	60434363	60435662	X:10	8	+	PHLPP1:intron
chr19	843995	844436	X:44	5	+	PRTN3:CDS:3
chr19	1881371	1881630	X:26	10	+	FAM108A1:5UTR

chr19	10973855	10974368	X:18	5	+	C19orf38:CDS:6
chr19	18959505	18960090	X:10	5	+	UPF1:intron
chr19	48697905	48698056	X:14	5	+	SNAR-A10:intron,SNAR-A7:intron,SNAR-A3:intron,SNAR-A4:intron,SNAR-A5:intron,SNAR-A14:intron,SNAR-A8:intron,SNAR-A6:intron,SNAR-A11:intron,SNAR-A9:intron
chr19	50733860	50734163	X:26	8	+	MYH14:CDS:8
chr19	54040763	54040981	X:16	8	+	ZNF331:promoter
chr2	2192365	2193064	X:13	5	+	MYT1L:intron
chr2	25856265	25856489	X:17	11	+	DTNB:intron
chr2	38292097	38293288	X:12	5	+	FAM82A1:intron
chr2	45233521	45233716	X:12	5	+	SIX2:CDS:2
chr2	113993259	113993459	X:17	6	+	PAX8:intron
chr2	116381431	116383945	X:16	5	+	DPP10:intron
chr2	145784723	145786449	X:14	9	+	DKFZp686O1327:intron
chr2	174082071	174083611	X:17	5	+	ZAK:intron,MLK7-AS1:intron
chr2	212505663	212509521	X:17	6	+	ERBB4:intron
chr2	241974983	241975744	X:53	5	+	SNED1:intron
chr20	14207308	14207631	X:23	6	+	MACROD2:intron
chr20	25465678	25467377	X:18	5	+	NINL:intron
chr20	33762456	33762765	X:21	7	+	PROCR:CDS:2
chr20	43378872	43379123	X:26	18	+	KCNK15:CDS:2
chr20	45565477	45565887	X:10	8	+	EYA2:intron
chr20	50148956	50149553	X:20	6	+	NFATC2:intron
chr20	59945924	59946145	X:14	6	+	CDH4:intron
chr20	61869763	61870145	X:14	5	+	BIRC7:CDS:3,BIRC7:CDS:4

chr21	38078137	38078611	X:13	5	+	SIM2:intron
chr21	39844618	39845040	X:17	5	+	ERG:intron
chr21	42857783	42858681	X:25	6	+	TMPRSS2:intron
chr21	43514227	43514492	X:12	6	+	UMODL1:intron
chr21	45842491	45843110	X:19	5	+	TRPM2:intron
chr21	47581358	47581585	X:26	8	+	C21orf56:CDS:3
chr22	43564539	43564895	X:12	5	+	TTLL12:CDS:13
chr22	43877620	43878421	X:24	7	+	MPPED1:intron
chr22	46786519	46786864	X:17	5	+	CELSR1:intron
chr3	238552	238828	X:34	11	+	CHL1:5UTR
chr3	23642450	23642986	X:10	5	+	MIR548AC:intron
chr3	32861156	32861711	X:33	7	+	TRIM71:intron
chr3	44622494	44622663	X:26	8	+	ZNF167:intron
chr3	49237239	49237433	X:10	5	+	CCDC36:intron
chr3	55516960	55517055	X:11	7	+	WNT5A:intron
chr3	66443810	66444357	X:13	6	+	LRIG1:intron
chr3	98241431	98241597	X:15	8	+	CLDND1:promoter
chr3	98241431	98241597	X:15	8	+	CLDND1:promoter,CLDND1:5UTR
chr3	114598092	114599107	X:12	5	+	ZBTB20:intron
chr3	125094085	125094231	X:10	10	+	ZNF148:promoter
chr3	125094085	125094231	X:10	10	+	ZNF148:promoter,ZNF148:5UTR
chr3	145878837	145879049	X:32	5	+	PLOD2:5UTR
chr3	173220028	173221738	X:11	5	+	NLGN1:intron
chr3	175388547	175390585	X:14	6	+	NAALADL2:intron

chr4	1994391	1994619	X:12	5	+	WHSC2:intron
chr4	2392109	2392954	X:19	5	+	ZFYVE28:intron
chr4	3372206	3372354	X:12	7	+	RGS12:intron
chr4	6458064	6458558	X:14	5	+	PPP2R2C:intron
chr4	38086668	38087531	X:15	5	+	TBC1D1:intron
chr4	56225056	56225878	X:13	6	+	SRD5A3:CDS:2
chr4	87994516	87995136	X:11	5	+	AFF1:intron
chr4	186508683	186509070	X:20	5	+	SORBS2:CDS:20
chr5	37839737	37840021	X:37	5	+	GDNF:promoter
chr5	37839737	37840021	X:37	5	+	GDNF:promoter,GDNF:5UTR
chr5	92907850	92908151	X:26	6	+	FLJ42709:intron
chr5	122434599	122434739	X:13	6	+	PRDM6:intron
chr5	158267292	158267780	X:10	6	+	EBF1:intron
chr5	177901014	177902439	X:16	5	+	COL23A1:intron
chr6	2102705	2103795	X:12	5	+	GMDS:intron
chr6	6003447	6003687	X:16	6	+	NRN1:intron
chr6	10612782	10614118	X:14	5	+	GCNT2:intron
chr6	35344623	35345149	X:13	6	+	PPARD:intron
chr6	41312104	41312735	X:16	5	+	NCR2:intron
chr6	54056383	54058669	X:13	6	+	MLIP:intron
chr6	64722688	64723267	X:12	7	+	EYS:intron
chr6	100897183	100897472	X:23	9	+	SIM1:CDS:5,SIM1:CDS:4
chr6	107366963	107367927	X:13	5	+	C6orf203:intron
chr6	144329192	144329386	X:31	21	+	PLAGL1:5UTR

chr6	149772191	149772625	X:48	8	+	ZC3H12D:CDS:5
chr6	152450797	152451266	X:11	5	+	SYNE1:intron
chr7	2749437	2749703	X:20	9	+	AMZ1:CDS:5
chr7	4910684	4911085	X:15	6	+	RADIL:intron
chr7	87187773	87190961	X:12	5	+	ABCB1:CDS:7
chr7	95546235	95546756	X:13	9	+	DYNC1I1:intron
chr7	97847264	97847722	X:23	6	+	TECPR1:CDS:22
chr7	101928663	101929277	X:17	5	+	SH2B2:intron
chr7	102554356	102554806	X:11	6	+	LRRC17:intron,FBXL13:intron
chr7	149462445	149462594	X:16	10	+	ZNF467:CDS:4
chr7	150498695	150498934	X:13	6	+	TMEM176B:promoter,TMEM176A:promoter
chr7	153868008	153870442	X:20	5	+	DPP6:intron
chr7	154862858	154863007	X:16	5	+	HTR5A:CDS:1
chr8	3276401	3277741	X:14	5	+	CSMD1:intron
chr8	11570972	11571439	X:10	6	+	GATA4:intron
chr8	18597602	18598096	X:10	7	+	PSD3:intron
chr8	22021201	22021677	X:20	10	+	BMP1:promoter
chr8	41593948	41594161	X:11	6	+	ANK1:intron
chr8	72270545	72272749	X:11	5	+	EYA1:intron
chr8	99007456	99009173	X:24	6	+	MATN2:intron
chr8	110424077	110425265	X:12	5	+	PKHD1L1:CDS:20
chr8	144810244	144810564	X:48	13	+	FAM83H:CDS:4
chr9	9182169	9183854	X:14	5	+	PTPRD:promoter
chr9	9182169	9183854	X:14	5	+	PTPRD:promoter,PTPRD:5UTR

chr9	13941463	13942084	X:10	5	+	C9orf146:intron
chr9	45727308	45727640	X:14	5	+	FAM27A:ncexon:1
chr9	45729607	45729811	X:14	5	+	LOC643648:intron,FAM75A7:intron,FAM75A5:intron,LOC286297:intron,ANKRD20A3:intron
chr9	66489407	66489685	X:17	9	+	LOC286297:intron,ANKRD20A3:intron
chr9	69193705	69196566	X:25	8	+	LOC100133920:intron
chr9	71618718	71620233	X:13	7	+	PIP5K1B:intron
chr9	124088439	124088949	X:16	6	+	GSN:CDS:11
chr9	126135610	126135842	X:23	8	+	CRB2:CDS:10
chr9	135361992	135362164	X:13	5	+	C9orf171:intron
chr9	136576801	136577354	X:25	6	+	SARDH:intron
chrX	9879646	9881135	X:33	11	+	SHROOM2:intron
chrX	48957894	48958259	X:32	6	+	WDR45:promoter
chrX	48957894	48958259	X:32	6	+	WDR45:promoter,WDR45:5UTR
chrX	144903843	144904183	X:12	6	+	SLITRK2:promoter
chrX	144903843	144904183	X:12	6	+	SLITRK2:promoter,SLITRK2:5UTR

Supplementary Table 2：The genes with DMRs which located in the gene promoter by DiffMeth method analysis

gene symbol
ACTB
ALDH5A1
ASS1
AZI2
B3GALNT2
BDNF
BMF
BMP1
C15orf62
C18orf8
C5orf42
C7orf10
C7orf11
CACNB3
CLDND1
CMPK1
CMTM2
CNKSR2
COL1A2
CPSF4
DAPK1

DEGS1
DENND1A
DKK1
DOK1
DYNC1H1
FAM64A
FAR1
FBXW11
FGF8
GCC1
GDNF
GNAI2
GPC5
GRM4
HMX2
INPP5F
ISYNA1
KCNE4
KIAA1377
KLK7
KRT9
LGALS8
LHFPL2
LOXL3

LTB4R
LYSMD4
MAGEL2
MDK
MEGF9
MEST
MRPL12
MRPS5
NHLRC1
NHP2L1
NLRP3
NOC4L
NOX5
OBFC2A
PAX1
PDHA1
PEX10
PTCD1
PTPRD
PTPRS
RABEP2
RABGAP1L
RBM11
REEP5

RUNDC3B
RUNX3
SIGMAR1
SKP1
SLAIN1
SLITRK2
SNAP47
SNRPD2
SNRPE
SNRPN
SNURF
SORD
SP5
SPESP1
SRPRB
SYAP1
SYNJ2
TAGAP
TCF12
TIMM8A
TMEM176A
TMEM176B
TMEM37
TP73

TRIM7
TSC2
VRK1
WDR45
WNT2B
ZIC4
ZNF148
ZNF18
ZNF184
ZNF286B
ZNF331
ZNF544
ZSCAN1

Supplementary Table 3： The gene related hyper-methylated DMRs in the OST-7D group compared to OST-0D group

Chr	Start	End	CpG sites	Num-sites	the value of Meth-diff	Gene associated regions
chr1	3154748	3155412	X:30	7	+	PRDM16:intron
chr1	6531040	6531196	X:16	5	+	PLEKHG5:CDS:12
chr1	17092499	17092748	X:10	7	+	MIR3675:intron
chr1	21864787	21864977	X:14	5	+	ALPL:intron
chr1	23720292	23720842	X:16	6	+	TCEA3:CDS:8
chr1	25894682	25895381	X:22	5	+	LDLRAP1:3UTR
chr1	61914545	61915001	X:11	6	+	NFIA:intron
chr1	92216106	92217483	X:11	6	+	TGFBR3:intron
chr1	160054294	160054373	X:13	5	+	KCNJ9:CDS:1
chr1	174843541	174844010	X:10	6	+	RABGAP1L:promoter,RABGAP1L:promoter
chr1	174843541	174844010	X:10	6	+	RABGAP1L:promoter,RABGAP1L:promoter,RABGAP1L:5UTR
chr1	198901566	198903300	X:12	5	+	LOC100131234:intron
chr1	204653481	204653718	X:11	5	+	LRRN2:intron
chr1	245462133	245462493	X:13	7	+	KIF26B:intron
chr10	21144399	21145514	X:10	5	+	NEBL:intron
chr10	24416323	24417665	X:13	5	+	KIAA1217:intron
chr10	30316299	30316709	X:16	9	+	KIAA1462:CDS:2
chr10	85955263	85956268	X:23	7	+	CDHR1:CDS:2,CDHR1:CDS:3
chr10	114712357	114712663	X:19	6	+	TCF7L2:intron
chr10	124907550	124907762	X:26	7	+	HMX2:promoter

chr10	124907550	124907762	X:26	7	+	HMX2:promoter,HMX2:5UTR
chr10	131749397	131749973	X:20	6	+	EBF3:intron
chr11	17786117	17786797	X:21	6	+	KCNC1:intron
chr11	44325258	44325886	X:33	12	+	ALX4:intron
chr11	46384634	46385376	X:25	8	+	DGKZ:intron
chr11	46401392	46401701	X:17	5	+	MDK:promoter
chr11	61481086	61481638	X:14	5	+	DAGLA:intron
chr11	66062246	66062366	X:20	6	+	TMEM151A:CDS:2
chr11	68147850	68148079	X:17	6	+	LRP5:intron
chr11	101783582	101785113	X:10	5	+	KIAA1377:promoter
chr11	122597619	122598238	X:14	6	+	UBASH3B:intron
chr12	2339324	2339614	X:30	9	+	CACNA1C:intron
chr12	49943271	49943434	X:17	5	+	KCNH3:CDS:9
chr12	94659159	94659830	X:11	5	+	PLXNC1:intron
chr12	115107577	115109504	X:28	7	+	TBX3:3UTR
chr12	116482142	116483522	X:10	5	+	MED13L:intron
chr12	124767826	124768330	X:12	5	+	ZNF664-FAM101A:intron
chr13	30077279	30077552	X:22	8	+	MTUS2:CDS:14
chr13	33896239	33896912	X:13	7	+	STARD13:intron
chr13	36642214	36644493	X:14	5	+	DCLK1:intron
chr13	92050752	92050960	X:30	6	+	GPC5:promoter
chr13	92050752	92050960	X:30	6	+	GPC5:promoter,GPC5:5UTR
chr13	101187793	101188225	X:12	6	+	A2LD1:intron
chr13	111058009	111058695	X:12	5	+	COL4A2:intron

chr13	114076067	114076719	X:19	5	+	ADPRHL1:3UTR
chr14	24109492	24110335	X:10	6	+	DHRS2:intron
chr14	56683528	56684516	X:11	6	+	PELI2:intron
chr14	62214248	62217116	X:11	5	+	HIF1A:3UTR
chr14	72467523	72468515	X:13	8	+	RGS6:intron
chr14	98405047	98408445	X:23	5	+	C14orf64:intron
chr15	23892844	23893075	X:10	6	+	MAGEL2:promoter
chr15	23892844	23893075	X:10	6	+	MAGEL2:promoter,MAGEL2:5UTR
chr15	57510119	57511991	X:14	5	+	TCF12:promoter
chr15	57510119	57511991	X:14	5	+	TCF12:promoter,TCF12:5UTR
chr15	58357369	58357534	X:15	7	+	ALDH1A2:intron
chr15	69222761	69222903	X:16	7	+	SPESP1:promoter,NOX5:promoter
chr15	69222761	69222903	X:16	7	+	SPESP1:promoter,NOX5:promoter,SPESP1:5UTR,NOX5:5UTR
chr15	71999914	72001373	X:17	5	+	THSD4:intron
chr15	84505535	84506987	X:13	7	+	ADAMTSL3:CDS:6
chr15	89935336	89938133	X:16	5	+	LOC254559:intron
chr15	92692240	92692896	X:12	5	+	SLCO3A1:intron
chr15	100674236	100675270	X:14	6	+	ADAMTS17:intron
chr15	101729470	101729708	X:10	7	+	CHSY1:intron
chr16	4290662	4290972	X:11	5	+	SRL:intron
chr16	9914611	9916143	X:13	6	+	GRIN2A:CDS:9
chr16	11654526	11655300	X:21	6	+	LITAF:intron
chr16	50589796	50590368	X:18	8	+	NKD1:intron
chr16	78427350	78428596	X:11	6	+	WWOX:intron

chr16	84336085	84336417	X:13	6	+	WFDC1:intron
chr16	86547739	86548894	X:16	5	+	FOXF1:3UTR
chr16	88747897	88748067	X:13	6	+	SNAI3:CDS:2
chr16	89411466	89412009	X:17	5	+	ANKRD11:intron
chr17	1084457	1084956	X:14	6	+	ABR:intron
chr17	30953875	30954650	X:14	5	+	MYO1D:intron
chr17	31810160	31811046	X:13	5	+	ACCN1:intron
chr17	39728143	39728454	X:15	5	+	KRT9:promoter
chr17	39728143	39728454	X:15	5	+	KRT9:promoter,KRT9:5UTR
chr17	43323892	43324256	X:10	6	+	FMNL1:CDS:26
chr17	46628046	46628360	X:22	5	+	HOXB3:CDS:2
chr17	46667304	46667949	X:20	7	+	HOXB-AS3:ncexon:1
chr17	58164409	58165865	X:13	6	+	LOC645638:ncexon:1
chr17	66596122	66596436	X:24	10	+	FAM20A:5UTR
chr17	75318260	75318786	X:17	5	+	SEPT9:intron
chr17	78560453	78560825	X:20	11	+	RPTOR:intron
chr17	78894313	78894844	X:21	9	+	RPTOR:intron
chr17	79011390	79011661	X:12	6	+	BAIAP2:intron
chr17	80805852	80806603	X:28	7	+	TBCD:intron
chr18	447007	449695	X:21	5	+	COLEC12:intron
chr18	46895113	46895480	X:10	6	+	DYM:intron
chr18	55810522	55811072	X:15	5	+	NEDD4L:intron
chr18	60434363	60435662	X:10	8	+	PHLPP1:intron
chr19	843995	844436	X:44	5	+	PRTN3:CDS:3

chr19	1881371	1881630	X:26	10	+	FAM108A1:5UTR
chr19	10973855	10974368	X:18	5	+	C19orf38:CDS:6
chr19	18959505	18960090	X:10	5	+	UPF1:intron
chr19	48697905	48698056	X:14	5	+	SNAR-A10:intron,SNAR-A7:intron,SNAR-A3:intron,SNAR-A4:intron,SNAR-A5:intron,SNAR-A14:intron,SNAR-A8:intron,SNAR-A6:intron,SNAR-A11:intron,SNAR-A9:intron
chr19	50733860	50734163	X:26	8	+	MYH14:CDS:8
chr19	54040763	54040981	X:16	8	+	ZNF331:promoter
chr2	2192365	2193064	X:13	5	+	MYT1L:intron
chr2	25856265	25856489	X:17	11	+	DTNB:intron
chr2	38292097	38293288	X:12	5	+	FAM82A1:intron
chr2	45233521	45233716	X:12	5	+	SIX2:CDS:2
chr2	113993259	113993459	X:17	6	+	PAX8:intron
chr2	116381431	116383945	X:16	5	+	DPP10:intron
chr2	145784723	145786449	X:14	9	+	DKFZp686O1327:intron
chr2	174082071	174083611	X:17	5	+	ZAK:intron,MLK7-AS1:intron
chr2	212505663	212509521	X:17	6	+	ERBB4:intron
chr2	241974983	241975744	X:53	5	+	SNED1:intron
chr20	14207308	14207631	X:23	6	+	MACROD2:intron
chr20	25465678	25467377	X:18	5	+	NINL:intron
chr20	33762456	33762765	X:21	7	+	PROCR:CDS:2
chr20	43378872	43379123	X:26	18	+	KCNK15:CDS:2
chr20	45565477	45565887	X:10	8	+	EYA2:intron
chr20	50148956	50149553	X:20	6	+	NFATC2:intron
chr20	59945924	59946145	X:14	6	+	CDH4:intron

chr20	61869763	61870145	X:14	5	+	BIRC7:CDS:3,BIRC7:CDS:4
chr21	38078137	38078611	X:13	5	+	SIM2:intron
chr21	39844618	39845040	X:17	5	+	ERG:intron
chr21	42857783	42858681	X:25	6	+	TMPRSS2:intron
chr21	43514227	43514492	X:12	6	+	UMODL1:intron
chr21	45842491	45843110	X:19	5	+	TRPM2:intron
chr21	47581358	47581585	X:26	8	+	C21orf56:CDS:3
chr22	43564539	43564895	X:12	5	+	TTLL12:CDS:13
chr22	43877620	43878421	X:24	7	+	MPPED1:intron
chr22	46786519	46786864	X:17	5	+	CELSR1:intron
chr3	238552	238828	X:34	11	+	CHL1:5UTR
chr3	23642450	23642986	X:10	5	+	MIR548AC:intron
chr3	32861156	32861711	X:33	7	+	TRIM71:intron
chr3	44622494	44622663	X:26	8	+	ZNF167:intron
chr3	49237239	49237433	X:10	5	+	CCDC36:intron
chr3	55516960	55517055	X:11	7	+	WNT5A:intron
chr3	66443810	66444357	X:13	6	+	LRIG1:intron
chr3	98241431	98241597	X:15	8	+	CLDND1:promoter
chr3	98241431	98241597	X:15	8	+	CLDND1:promoter,CLDND1:5UTR
chr3	114598092	114599107	X:12	5	+	ZBTB20:intron
chr3	125094085	125094231	X:10	10	+	ZNF148:promoter
chr3	125094085	125094231	X:10	10	+	ZNF148:promoter,ZNF148:5UTR
chr3	145878837	145879049	X:32	5	+	PLOD2:5UTR
chr3	173220028	173221738	X:11	5	+	NLGN1:intron

chr3	175388547	175390585	X:14	6	+	NAALADL2:intron
chr4	1994391	1994619	X:12	5	+	WHSC2:intron
chr4	2392109	2392954	X:19	5	+	ZFYVE28:intron
chr4	3372206	3372354	X:12	7	+	RGS12:intron
chr4	6458064	6458558	X:14	5	+	PPP2R2C:intron
chr4	38086668	38087531	X:15	5	+	TBC1D1:intron
chr4	56225056	56225878	X:13	6	+	SRD5A3:CDS:2
chr4	87994516	87995136	X:11	5	+	AFF1:intron
chr4	186508683	186509070	X:20	5	+	SORBS2:CDS:20
chr5	37839737	37840021	X:37	5	+	GDNF:promoter
chr5	37839737	37840021	X:37	5	+	GDNF:promoter,GDNF:5UTR
chr5	92907850	92908151	X:26	6	+	FLJ42709:intron
chr5	122434599	122434739	X:13	6	+	PRDM6:intron
chr5	158267292	158267780	X:10	6	+	EBF1:intron
chr5	177901014	177902439	X:16	5	+	COL23A1:intron
chr6	2102705	2103795	X:12	5	+	GMDS:intron
chr6	6003447	6003687	X:16	6	+	NRN1:intron
chr6	10612782	10614118	X:14	5	+	GCNT2:intron
chr6	35344623	35345149	X:13	6	+	PPARD:intron
chr6	41312104	41312735	X:16	5	+	NCR2:intron
chr6	54056383	54058669	X:13	6	+	MLIP:intron
chr6	64722688	64723267	X:12	7	+	EYS:intron
chr6	100897183	100897472	X:23	9	+	SIM1:CDS:5,SIM1:CDS:4
chr6	107366963	107367927	X:13	5	+	C6orf203:intron

chr6	144329192	144329386	X:31	21	+	PLAGL1:5UTR
chr6	149772191	149772625	X:48	8	+	ZC3H12D:CDS:5
chr6	152450797	152451266	X:11	5	+	SYNE1:intron
chr7	2749437	2749703	X:20	9	+	AMZ1:CDS:5
chr7	4910684	4911085	X:15	6	+	RADIL:intron
chr7	87187773	87190961	X:12	5	+	ABCB1:CDS:7
chr7	95546235	95546756	X:13	9	+	DYNC1I1:intron
chr7	97847264	97847722	X:23	6	+	TECPR1:CDS:22
chr7	101928663	101929277	X:17	5	+	SH2B2:intron
chr7	102554356	102554806	X:11	6	+	LRRC17:intron,FBXL13:intron
chr7	149462445	149462594	X:16	10	+	ZNF467:CDS:4
chr7	150498695	150498934	X:13	6	+	TMEM176B:promoter,TMEM176A:promoter
chr7	153868008	153870442	X:20	5	+	DPP6:intron
chr7	154862858	154863007	X:16	5	+	HTR5A:CDS:1
chr8	3276401	3277741	X:14	5	+	CSMD1:intron
chr8	11570972	11571439	X:10	6	+	GATA4:intron
chr8	18597602	18598096	X:10	7	+	PSD3:intron
chr8	22021201	22021677	X:20	10	+	BMP1:promoter
chr8	41593948	41594161	X:11	6	+	ANK1:intron
chr8	72270545	72272749	X:11	5	+	EYA1:intron
chr8	99007456	99009173	X:24	6	+	MATN2:intron
chr8	110424077	110425265	X:12	5	+	PKHD1L1:CDS:20
chr8	144810244	144810564	X:48	13	+	FAM83H:CDS:4
chr9	9182169	9183854	X:14	5	+	PTPRD:promoter

chr9	9182169	9183854	X:14	5	+	PTPRD:promoter,PTPRD:5UTR
chr9	13941463	13942084	X:10	5	+	C9orf146:intron
chr9	45727308	45727640	X:14	5	+	FAM27A:ncexon:1
chr9	45729607	45729811	X:14	5	+	LOC643648:intron,FAM75A7:intron,FAM75A5:intron,LOC286297:intron,ANKRD20A3:intron
chr9	66489407	66489685	X:17	9	+	LOC286297:intron,ANKRD20A3:intron
chr9	69193705	69196566	X:25	8	+	LOC100133920:intron
chr9	71618718	71620233	X:13	7	+	PIP5K1B:intron
chr9	124088439	124088949	X:16	6	+	GSN:CDS:11
chr9	126135610	126135842	X:23	8	+	CRB2:CDS:10
chr9	135361992	135362164	X:13	5	+	C9orf171:intron
chr9	136576801	136577354	X:25	6	+	SARDH:intron
chrX	9879646	9881135	X:33	11	+	SHROOM2:intron
chrX	48957894	48958259	X:32	6	+	WDR45:promoter
chrX	48957894	48958259	X:32	6	+	WDR45:promoter,WDR45:5UTR
chrX	144903843	144904183	X:12	6	+	SLITRK2:promoter
chrX	144903843	144904183	X:12	6	+	SLITRK2:promoter,SLITRK2:5UTR

Supplementary Table 4：The genes with hyper-methylated DMRs in the OST-7D group which located in the gene promoter

gene symbol
BMP1
CLDND1
GDNF
GPC5
HMX2
KIAA1377
KRT9
MAGEL2
MDK
NOX5
PTPRD
RABGAP1L
SLITRK2
SPESP1
TCF12
TMEM176A
TMEM176B
WDR45
ZNF148
ZNF331

Supplementary Table 5: The gene related hypor-methylated DMRs in the OST-7D group compared to OST-0D group

Chr	Start	End	CpG sites	Num-sites	the value of Meth-diff	Gene associated regions
chr1	2344140	2344889	X:76	9	+	PEX10:promoter
chr1	2424829	2425255	X:22	8	+	PLCH2:intron
chr1	3061790	3062910	X:50	7	+	PRDM16:intron
chr1	3164511	3164930	X:18	12	+	PRDM16:intron
chr1	3568495	3568855	X:39	12	+	TP73:promoter
chr1	6305277	6305464	X:20	9	+	HES3:CDS:3
chr1	9713439	9713749	X:11	5	+	C1orf200:ncexon:2
chr1	23066258	23066696	X:17	10	+	EPHB2:intron
chr1	25257599	25257930	X:25	8	+	RUNX3:promoter
chr1	26026292	26027038	X:10	7	+	MAN1C1:intron
chr1	32135441	32135801	X:11	5	+	COL16A1:intron
chr1	39871261	39873833	X:26	5	+	MACF1:intron
chr1	47799412	47799486	X:14	8	+	CMPK1:promoter
chr1	47799412	47799486	X:14	8	+	CMPK1:promoter,CMPK1:5UTR
chr1	52455818	52456010	X:13	5	+	RAB3B:intron
chr1	89457203	89457641	X:18	13	+	RBMXL1:intron,CCBL2:intron
chr1	113051385	113051519	X:21	7	+	WNT2B:promoter
chr1	113051385	113051519	X:21	7	+	WNT2B:promoter,WNT2B:5UTR
chr1	119521629	119522501	X:43	6	+	TBX15:intron
chr1	146895262	146895660	X:12	5	+	LOC100289211:intron,FLJ39739:intron,LOC728855:intron,PPIAL4B:intron,NBPF11:intron,PPIAL4A:intron,GPR89C:intron,NBPF24:intron,PDZK1P1:intron

chr1	149604553	149606699	X:70	5	+	LOC728855:intron,FAM91A2:intron,HIST2H2BF:intron
chr1	156815427	156815904	X:39	10	+	INSRR:CDS:10,INSRR:CDS:9
chr1	165321402	165322172	X:46	11	+	LMX1A:intron
chr1	165323191	165323732	X:36	13	+	LMX1A:intron
chr1	166890456	166890693	X:26	7	+	ILDR2:CDS:9
chr1	170640024	170640754	X:29	5	+	PRRX1:intron
chr1	203829693	203830300	X:23	10	+	SNRPE:promoter
chr1	214476555	214477131	X:15	7	+	SMYD2:intron
chr1	224370938	224371082	X:16	8	+	DEGS1:promoter
chr1	224370938	224371082	X:16	8	+	DEGS1:promoter,DEGS1:5UTR
chr1	226411357	226411539	X:27	5	+	MIXL1:CDS:1
chr1	227922721	227922985	X:32	6	+	SNAP47:promoter
chr1	227922721	227922985	X:32	6	+	SNAP47:promoter,SNAP47:5UTR
chr1	228401244	228401518	X:33	6	+	OBSCN:CDS:2
chr1	235667482	235667755	X:36	7	+	B3GALNT2:promoter
chr1	235667482	235667755	X:36	7	+	B3GALNT2:promoter,B3GALNT2:5UTR
chr1	236686011	236686929	X:23	10	+	LGALS8:promoter
chr1	236686011	236686929	X:23	10	+	LGALS8:promoter,LGALS8:5UTR
chr1	247579508	247580310	X:17	8	+	NLRP3:promoter
chr10	6213830	6214153	X:14	8	+	PFKFB3:intron
chr10	35736721	35737318	X:14	6	+	CCNY:intron
chr10	49879397	49880157	X:37	12	+	FAM21B:intron
chr10	54074079	54074336	X:18	12	+	DKK1:promoter
chr10	54074079	54074336	X:18	12	+	DKK1:promoter,DKK1:5UTR

chr10	74094093	74094869	X:26	5	+	DNAJB12:3UTR
chr10	78011602	78012721	X:15	5	+	C10orf11:intron
chr10	102586628	102586778	X:16	9	+	PAX2:CDS:10
chr10	103534171	103534715	X:19	6	+	FGF8:promoter
chr10	103534171	103534715	X:19	6	+	FGF8:promoter,FGF8:5UTR
chr10	121578172	121578402	X:28	11	+	INPP5F:promoter
chr10	125447737	125450617	X:19	6	+	GPR26:3UTR
chr10	126840910	126841148	X:16	6	+	CTBP2:intron
chr10	134980773	134980953	X:17	5	+	KNDC1:CDS:2
chr11	1948028	1949013	X:29	7	+	TNNT3:intron
chr11	2159301	2159451	X:21	6	+	IGF2:intron,INS-IGF2:intron
chr11	2720829	2721027	X:29	7	+	KCNQ1OT1:ncexon:1
chr11	13032404	13032746	X:20	8	+	RASSF10:CDS:1
chr11	13689903	13690088	X:19	15	+	FAR1:promoter
chr11	27722013	27722332	X:38	6	+	BDNF:promoter
chr11	27722013	27722332	X:38	6	+	BDNF:promoter,BDNF:5UTR,BDNF:5UTR
chr11	64120582	64120895	X:17	6	+	CCDC88B:CDS:21,CCDC88B:CDS:22
chr11	66193851	66194008	X:13	6	+	NPAS4:3UTR
chr11	69518419	69518639	X:26	6	+	FGF19:CDS:1
chr12	3345678	3346257	X:14	5	+	TSPAN9:intron
chr12	42983100	42983268	X:15	11	+	PRICKLE1:5UTR
chr12	49208212	49208388	X:15	8	+	CACNB3:promoter,CACNB3:promoter
chr12	49208212	49208388	X:15	8	+	CACNB3:promoter,CACNB3:promoter,CACNB3:5UTR
chr12	58239441	58239680	X:23	5	+	CTDSP2:intron

chr12	94580367	94580738	X:19	5	+	PLXNC1:intron
chr12	115109568	115109701	X:12	6	+	TBX3:CDS:7
chr12	115112443	115112608	X:18	6	+	TBX3:CDS:6
chr12	120105873	120106085	X:19	7	+	PRKAB1:5UTR
chr12	124242063	124242821	X:10	7	+	ATP6V0A2:CDS:20
chr12	124873802	124874003	X:17	10	+	NCOR2:intron
chr12	132628607	132628711	X:19	5	+	NOC4L:promoter
chr13	78272441	78272829	X:44	6	+	SLAIN1:promoter
chr13	109806752	109808000	X:20	8	+	MYO16:intron
chr13	110953975	110954437	X:13	6	+	COL4A1:intron
chr13	114565770	114565961	X:17	7	+	GAS6:intron
chr14	24780256	24780463	X:24	13	+	LTB4R:promoter
chr14	24780256	24780463	X:24	13	+	LTB4R:promoter,CIDEB:5UTR
chr14	55139594	55140493	X:11	5	+	SAMD4A:intron
chr14	74222353	74223554	X:18	5	+	C14orf43:intron
chr14	97263704	97264018	X:51	15	+	VRK1:promoter
chr14	97263704	97264018	X:51	15	+	VRK1:promoter,VRK1:5UTR
chr14	102430943	102431130	X:24	7	+	DYNC1H1:promoter
chr14	102430943	102431130	X:24	7	+	DYNC1H1:promoter,DYNC1H1:5UTR
chr15	20710460	20711337	X:42	5	+	HERC2P3:ncexon:2
chr15	25200003	25200775	X:54	13	+	SNRPN:promoter,SNURF:promoter
chr15	25200003	25200775	X:54	13	+	SNRPN:promoter,SNURF:promoter,SNRPN:5UTR,SNURF:5UTR
chr15	40399722	40400614	X:18	5	+	BMF:promoter
chr15	40399722	40400614	X:18	5	+	BMF:promoter,BMF:5UTR

chr15	41061478	41062200	X:22	5	+	C15orf62:promoter
chr15	41061478	41062200	X:22	5	+	C15orf62:promoter,C15orf62:5UTR
chr15	41787761	41788038	X:25	5	+	ITPKA:intron
chr15	45315202	45315418	X:16	9	+	SORD:promoter
chr15	45315202	45315418	X:16	9	+	SORD:promoter,SORD:5UTR
chr15	67053256	67054632	X:28	6	+	SMAD6:intron
chr15	74427108	74427865	X:40	9	+	ISLR2:CDS:1
chr15	76634317	76634559	X:14	12	+	ISL2:3UTR
chr15	86314770	86315413	X:12	5	+	KLHL25:intron
chr15	100273533	100273946	X:44	9	+	LYSMD4:promoter
chr15	100273533	100273946	X:44	9	+	LYSMD4:promoter,LYSMD4:5UTR
chr16	216315	216625	X:32	17	+	HBM:CDS:2,HBM:CDS:3
chr16	433504	434082	X:25	5	+	LOC100134368:intron
chr16	672297	672576	X:25	10	+	RAB40C:intron
chr16	1204446	1204878	X:21	6	+	CACNA1H:intron
chr16	2097589	2097729	X:13	11	+	TSC2:promoter
chr16	3304362	3304625	X:24	6	+	MEFV:CDS:2
chr16	24863072	24863842	X:14	6	+	SLC5A11:intron
chr16	27829315	27829980	X:19	6	+	GSG1L:intron
chr16	28936431	28936492	X:11	9	+	RABEP2:promoter
chr16	28936431	28936492	X:11	9	+	RABEP2:promoter,RABEP2:5UTR
chr16	55364261	55365019	X:30	11	+	IRX6:3UTR
chr16	66613134	66613355	X:32	7	+	CMTM2:promoter
chr16	66613134	66613355	X:32	7	+	CMTM2:promoter,CMTM2:5UTR

chr16	78537144	78538574	X:11	5	+	WWOX:intron
chr16	85648027	85648263	X:16	5	+	KIAA0182:intron
chr16	86612451	86612589	X:13	5	+	FOXL1:CDS:1
chr16	88540162	88540349	X:14	5	+	ZFPM1:intron
chr17	1969792	1970573	X:18	5	+	SMG6:intron
chr17	6347681	6347790	X:11	8	+	FAM64A:promoter
chr17	6347681	6347790	X:11	8	+	FAM64A:promoter,FAM64A:5UTR
chr17	7196058	7197162	X:18	6	+	YBX2:CDS:2
chr17	11900526	11900821	X:37	10	+	ZNF18:promoter
chr17	11900526	11900821	X:37	10	+	ZNF18:promoter,ZNF18:5UTR
chr17	18585445	18585650	X:17	10	+	ZNF286B:promoter
chr17	18585445	18585650	X:17	10	+	ZNF286B:promoter,ZNF286B:5UTR
chr17	37380676	37381143	X:37	21	+	STAC2:intron
chr17	59490308	59490809	X:26	6	+	C17orf82:ncexon:1
chr17	77395836	77396488	X:24	7	+	RBFOX3:intron
chr17	79093142	79093348	X:26	9	+	AATK:CDS:11
chr17	79670077	79670280	X:30	12	+	MRPL12:promoter
chr17	80290051	80290226	X:13	6	+	SECTM1:intron
chr17	80605959	80606237	X:31	5	+	WDR45L:5UTR
chr18	20715369	20715469	X:14	5	+	CABLES1:intron
chr18	21083110	21083275	X:15	12	+	C18orf8:promoter
chr19	1009580	1009797	X:14	5	+	GRIN3B:CDS:9
chr19	1465410	1465639	X:26	5	+	APC2:CDS:14
chr19	2241107	2241325	X:15	5	+	SF3A2:intron

chr19	3668289	3668603	X:14	6	+	PIP5K1C:intron
chr19	4368257	4369372	X:32	6	+	SH3GL1:intron
chr19	4976449	4976931	X:21	9	+	KDM4B:intron
chr19	5340953	5341042	X:14	9	+	PTPRS:promoter
chr19	6750259	6750505	X:15	6	+	TRIP10:CDS:12
chr19	12983791	12984483	X:45	10	+	MAST1:CDS:25,MAST1:CDS:26
chr19	14260390	14260774	X:19	6	+	LPHN1:3UTR
chr19	18548703	18548888	X:21	6	+	ISYNA1:promoter
chr19	18548703	18548888	X:21	6	+	ISYNA1:promoter,ISYNA1:5UTR
chr19	30866169	30866489	X:38	11	+	ZNF536:intron
chr19	34113283	34113373	X:14	5	+	CHST8:intron
chr19	40366340	40366575	X:26	6	+	FCGBP:CDS:30
chr19	40421120	40421435	X:32	8	+	FCGBP:CDS:5
chr19	46196061	46196194	X:11	8	+	SNRPD2:promoter
chr19	48946126	48946668	X:93	7	+	GRIN2D:CDS:12
chr19	49561304	49561963	X:26	7	+	SNAR-A10:intron,SNAR-A7:intron,SNAR-A3:intron,SNAR-A4:intron,SNAR-A5:intron,SNAR-A14:intron,SNAR-A8:intron,SNAR-A6:intron,SNAR-A11:intron,SNAR-A9:intron
chr19	49935752	49935999	X:23	6	+	SLC17A7:CDS:9
chr19	51486749	51487309	X:16	7	+	KLK7:promoter
chr19	51486749	51487309	X:16	7	+	KLK7:promoter,KLK7:5UTR,KLK7:5UTR
chr19	55763066	55763326	X:11	6	+	PPP6R1:intron
chr19	58545285	58545420	X:17	8	+	ZSCAN1:promoter
chr19	58740222	58740358	X:16	9	+	ZNF544:promoter

chr19	58740222	58740358	X:16	9	+	ZNF544:promoter,ZNF544:5UTR
chr19	58867468	58867766	X:36	6	+	ZNF497:CDS:1
chr19	58879211	58879349	X:18	7	+	ZNF837:CDS:1
chr2	66672616	66673226	X:42	12	+	MEIS1:intron
chr2	74729802	74729915	X:10	5	+	LBX2:CDS:1
chr2	74781352	74781451	X:12	5	+	DOK1:promoter,LOXL3:promoter
chr2	88124677	88125044	X:42	13	+	RGPD2:intron,RGPD1:intron
chr2	91844804	91845379	X:21	5	+	LOC654342:intron
chr2	95787634	95787789	X:20	13	+	MRPS5:promoter
chr2	95787634	95787789	X:20	13	+	MRPS5:promoter,MRPS5:5UTR
chr2	120189353	120189544	X:27	5	+	TMEM37:promoter
chr2	120189353	120189544	X:27	5	+	TMEM37:promoter,TMEM37:5UTR
chr2	130691761	130691931	X:17	12	+	LOC389033:ncexon:1
chr2	133014647	133014966	X:26	6	+	MIR663B:ncexon:1
chr2	171570943	171571199	X:26	6	+	SP5:promoter
chr2	177029537	177029757	X:21	6	+	HOXD3:intron
chr2	177036436	177036608	X:18	5	+	HOXD3:CDS:2
chr2	192542655	192542828	X:18	12	+	OBFC2A:promoter
chr2	192542655	192542828	X:18	12	+	OBFC2A:promoter,OBFC2A:5UTR
chr2	200330906	200332521	X:33	10	+	SATB2:intron
chr2	223917511	223917734	X:13	6	+	KCNE4:promoter
chr2	223917511	223917734	X:13	6	+	KCNE4:promoter,KCNE4:5UTR
chr2	228337096	228337435	X:39	10	+	AGFG1:5UTR
chr20	15897688	15898740	X:10	5	+	MACROD2:intron

chr20	21685400	21685841	X:20	8	+	PAX1:promoter
chr20	30135112	30135362	X:21	6	+	PSIMCT-1:ncexon:1
chr20	37434950	37435127	X:18	8	+	PPP1R16B:intron
chr20	57465119	57465388	X:31	6	+	GNAS:intron
chr20	57766930	57767281	X:39	5	+	ZNF831:CDS:1
chr20	62120430	62120605	X:16	5	+	EEF1A2:CDS:6
chr21	15588309	15588491	X:20	6	+	RBM11:promoter
chr21	15588309	15588491	X:20	6	+	RBM11:promoter,RBM11:5UTR
chr21	34443296	34443508	X:17	7	+	OLIG1:CDS:1
chr21	43812801	43813768	X:23	6	+	TMPRSS3:intron
chr21	45564710	45565141	X:16	5	+	C21orf33:CDS:6
chr21	46826425	46827096	X:27	6	+	COL18A1:intron
chr22	18064009	18064263	X:14	5	+	SLC25A18:CDS:3
chr22	36766602	36767424	X:15	6	+	MYH9:intron
chr22	37261500	37261766	X:13	6	+	NCF4:intron
chr22	42084782	42084933	X:16	16	+	NHP2L1:promoter
chr22	42084782	42084933	X:16	16	+	NHP2L1:promoter,NHP2L1:5UTR
chr22	48948274	48948708	X:16	5	+	FAM19A5:intron
chr3	28390613	28390932	X:24	9	+	AZI2:promoter
chr3	28390613	28390932	X:24	9	+	AZI2:promoter,AZI2:5UTR
chr3	45837675	45837847	X:18	10	+	SLC6A20:CDS:1
chr3	46975614	46977038	X:16	6	+	CCDC12:intron
chr3	47844666	47844836	X:14	8	+	DHX30:5UTR
chr3	50273544	50273744	X:26	9	+	GNAI2:promoter

chr3	50273544	50273744	X:26	9	+	GNAI2:promoter,GNAI2:5UTR
chr3	129305777	129306109	X:13	5	+	PLXND1:intron
chr3	133502608	133502725	X:10	5	+	SRPRB:promoter
chr3	147122696	147123473	X:22	8	+	ZIC4:promoter,ZIC4:promoter
chr3	147122696	147123473	X:22	8	+	ZIC4:promoter,ZIC4:promoter,ZIC4:5UTR
chr4	1188632	1188760	X:15	11	+	SPON2:intron
chr4	13529671	13530416	X:29	12	+	LOC285547:ncexon:3
chr4	13542995	13543676	X:23	6	+	NKX3-2:CDS:2
chr4	57458843	57458974	X:17	6	+	THEGL:intron
chr4	81110244	81110530	X:20	5	+	PRDM8:intron
chr4	166033652	166033878	X:22	6	+	TMEM192:intron
chr4	187071281	187071868	X:28	5	+	FAM149A:intron
chr4	187124575	187124894	X:10	7	+	CYP4V2:intron
chr5	2748664	2748828	X:21	9	+	IRX2:CDS:3
chr5	10649619	10650054	X:51	9	+	ANKRD33B:CDS:4
chr5	31937105	31937829	X:12	5	+	PDZD2:intron
chr5	37249426	37249598	X:23	18	+	C5orf42:promoter
chr5	37249426	37249598	X:23	18	+	C5orf42:promoter,C5orf42:5UTR
chr5	77944712	77945044	X:43	13	+	LHFPL2:promoter
chr5	92923024	92923551	X:16	5	+	NR2F1:intron
chr5	92924343	92926913	X:57	11	+	NR2F1:intron
chr5	112257909	112258099	X:30	9	+	REEP5:promoter
chr5	112257909	112258099	X:30	9	+	REEP5:promoter,REEP5:5UTR
chr5	122434853	122435523	X:47	7	+	PRDM6:CDS:2

chr5	133513400	133513712	X:14	6	+	SKP1:promoter
chr5	135416032	135416725	X:34	5	+	VTRNA2-1:ncexon:1
chr5	140242203	140242414	X:24	6	+	PCDHA9:intron,PCDHA6:intron,PCDHA8:intron,PCDHA5:intron,PCDHA2:intron,PCDHA4:intron,PCDHA10:intron,PCDHA1:intron,PCDHA7:intron,PCDHA3:intron
chr5	159688055	159688772	X:19	5	+	CCNJL:intron
chr5	171433660	171433781	X:11	5	+	FBXW11:promoter
chr5	171433660	171433781	X:11	5	+	FBXW11:promoter,FBXW11:5UTR
chr5	171614782	171614925	X:16	9	+	STK10:CDS:1
chr5	180632027	180632401	X:42	24	+	TRIM7:promoter
chr5	180632027	180632401	X:42	24	+	TRIM7:promoter,TRIM7:5UTR
chr6	12291677	12292901	X:18	6	+	EDN1:CDS:2
chr6	18122832	18123164	X:28	11	+	NHLRC1:promoter
chr6	18122832	18123164	X:28	11	+	NHLRC1:promoter,NHLRC1:5UTR
chr6	24495219	24495442	X:30	7	+	ALDH5A1:promoter
chr6	24495219	24495442	X:30	7	+	ALDH5A1:promoter,ALDH5A1:5UTR
chr6	27441662	27441892	X:13	9	+	ZNF184:promoter
chr6	34113719	34114207	X:18	5	+	GRM4:promoter
chr6	34113719	34114207	X:18	5	+	GRM4:promoter,GRM4:5UTR
chr6	74008935	74009455	X:41	6	+	KHDC1:intron,C6orf147:intron
chr6	158438166	158438463	X:14	5	+	SYNJ2:promoter
chr6	158438166	158438463	X:14	5	+	SYNJ2:promoter,SYNJ2:5UTR
chr6	159465764	159466690	X:18	6	+	TAGAP:promoter
chr6	159465764	159466690	X:18	6	+	TAGAP:promoter,TAGAP:5UTR
chr6	160427262	160427616	X:23	13	+	IGF2R:intron

chr7	1094657	1094970	X:20	7	+	C7orf50:intron
chr7	1272905	1273301	X:42	14	+	UNCX:CDS:1,UNCX:CDS:2
chr7	4831752	4832660	X:72	5	+	KIAA0415:3UTR
chr7	5569549	5569825	X:43	5	+	ACTB:promoter
chr7	5821071	5821244	X:20	15	+	RNF216:5UTR
chr7	27206427	27206711	X:14	6	+	HOXA10-HOXA9:intron
chr7	40174597	40174734	X:14	6	+	C7orf11:promoter,C7orf10:promoter
chr7	40174597	40174734	X:14	6	+	C7orf11:promoter,C7orf10:promoter,C7orf10:5UTR
chr7	66532130	66532662	X:10	5	+	TYW1:CDS:10
chr7	87257532	87257703	X:21	5	+	RUNDC3B:promoter
chr7	94023618	94023924	X:18	5	+	COL1A2:promoter
chr7	94023618	94023924	X:18	5	+	COL1A2:promoter,COL1A2:5UTR
chr7	96631679	96632140	X:20	9	+	DLX6-AS1:intron
chr7	99036585	99036945	X:38	13	+	CPSF4:promoter,PTCD1:promoter
chr7	99036585	99036945	X:38	13	+	CPSF4:promoter,PTCD1:promoter,CPSF4:5UTR
chr7	100203398	100203599	X:18	6	+	PCOLCE:CDS:5
chr7	100823267	100823455	X:16	6	+	NAT16:5UTR
chr7	101049158	101049511	X:15	6	+	EMID2:intron
chr7	127225542	127225723	X:18	10	+	GCC1:promoter
chr7	127225542	127225723	X:18	10	+	GCC1:promoter,GCC1:5UTR
chr7	130130739	130130995	X:26	11	+	MEST:promoter
chr7	145945389	145946093	X:22	5	+	CNTNAP2:intron
chr7	157406375	157406737	X:30	6	+	PTPRN2:intron
chr7	157576860	157577667	X:29	5	+	PTPRN2:intron

chr7	157668518	157669348	X:21	6	+	PTPRN2:intron
chr8	1496930	1497185	X:35	22	+	DLGAP2:CDS:1
chr8	1950836	1951059	X:32	6	+	KBTBD11:CDS:1
chr8	8750331	8750534	X:22	9	+	MFHAS1:CDS:1
chr8	11272335	11272886	X:10	5	+	C8orf12:intron
chr8	22635193	22635576	X:13	6	+	PEBP4:intron
chr8	25896195	25897234	X:25	5	+	EBF2:intron
chr9	34637478	34637802	X:36	8	+	SIGMAR1:promoter
chr9	34637478	34637802	X:36	8	+	SIGMAR1:promoter,SIGMAR1:5UTR
chr9	90112861	90113035	X:18	5	+	DAPK1:promoter
chr9	90112861	90113035	X:18	5	+	DAPK1:promoter,DAPK1:5UTR
chr9	123476699	123476903	X:29	9	+	MEGF9:promoter
chr9	123476699	123476903	X:29	9	+	MEGF9:promoter,MEGF9:5UTR
chr9	126692428	126692758	X:30	9	+	DENND1A:promoter
chr9	130684061	130684177	X:15	15	+	PIP5KL1:CDS:4
chr9	131012953	131013323	X:36	8	+	DNM1:CDS:20
chr9	131939245	131939446	X:22	5	+	IER5L:CDS:1
chr9	133320154	133320631	X:48	10	+	ASS1:promoter
chr9	133320154	133320631	X:48	10	+	ASS1:promoter,ASS1:5UTR
chr9	138078062	138079193	X:30	5	+	LOC401557:ncexon:3
chrX	16737358	16737799	X:28	12	+	SYAP1:promoter
chrX	16737358	16737799	X:28	12	+	SYAP1:promoter,SYAP1:5UTR
chrX	19362105	19362392	X:29	5	+	PDHA1:promoter
chrX	19362105	19362392	X:29	5	+	PDHA1:promoter,PDHA1:5UTR

chrX	21392042	21392377	X:36	5	+	CNKSR2:promoter
chrX	100603685	100603839	X:19	11	+	TIMM8A:promoter
chrX	100603685	100603839	X:19	11	+	TIMM8A:promoter,TIMM8A:5UTR
chrX	153043745	153044220	X:19	6	+	PLXNB3:CDS:32,PLXNB3:CDS:33,PLXNB3:CDS:34

Supplementary Table 6: The genes with hypor-methylated DMRs in the OST-7D group which located in the gene promoter

gene symble
ACTB
ALDH5A1
ASS1
AZI2
B3GALNT2
BDNF
BMF
C15orf62
C18orf8
C5orf42
C7orf10
C7orf11
CACNB3
CMPK1
CMTM2
CNKSR2
COL1A2
CPSF4
DAPK1
DEGS1
DENND1A

DKK1
DOK1
DYNC1H1
FAM64A
FAR1
FBXW11
FGF8
GCC1
GNAI2
GRM4
INPP5F
ISYNA1
KCNE4
KLK7
LGALS8
LHFPL2
LOXL3
LTB4R
LYSMD4
MEGF9
MEST
MRPL12
MRPS5
NHLRC1

NHP2L1
NLRP3
NOC4L
OBFC2A
PAX1
PDHA1
PEX10
PTCD1
PTPRS
RABEP2
RBM11
REEP5
RUNDC3B
RUNX3
SIGMAR1
SKP1
SLAIN1
SNAP47
SNRPD2
SNRPE
SNRPN
SNURF
SORD
SP5

SRPRB
SYAP1
SYNJ2
TAGAP
TIMM8A
TMEM37
TP73
TRIM7
TSC2
VRK1
WNT2B
ZIC4
ZNF18
ZNF184
ZNF286B
ZNF544
ZSCAN1

Supplementary Table 7： The genes related DMRs between the OST-0D group and the OST-7D group by Radmeth methods analysis

Chr	Start	End	Dmr	Num-sites	the value of Meth-diff	Gene associated regions
chr1	567205	567766	dmr	10	-0.0229464	LOC100133331:intron
chr1	2425192	2425202	dmr	1	-0.607407	PLCH2:intron
chr1	3164571	3164765	dmr	2	-0.368367	PRDM16:intron
chr1	8698168	8698169	dmr	1	0.355556	RERE:intron
chr1	12365000	12365001	dmr	1	0.363636	VPS13D:intron
chr1	17091920	17091983	dmr	3	0.350362	MIR3675:intron
chr1	23066310	23066366	dmr	3	-0.541667	EPHB2:intron
chr1	23720400	23720628	dmr	5	0.532581	TCEA3:CDS:8
chr1	28334835	28335026	dmr	2	0.350625	EYA3:intron
chr1	35344960	35345076	dmr	2	0.391597	DLGAP3:intron
chr1	35739062	35739067	dmr	2	0.289157	ZMYM4:intron
chr1	35853316	35853331	dmr	1	0.246359	ZMYM4:intron
chr1	39346611	39346788	dmr	2	0.309054	GJA9-MYCBP:intron
chr1	41553154	41553155	dmr	1	0.344615	SCMH1:intron
chr1	42790151	42790245	dmr	3	0.317187	FOXJ3:promoter
chr1	48819122	48819123	dmr	1	0.421429	SPATA6:intron
chr1	48930522	48930663	dmr	1	0.29708	SPATA6:intron
chr1	52759689	52759690	dmr	1	0.227273	ZFYVE9:intron
chr1	59002496	59002630	dmr	4	0.304056	OMA1:intron
chr1	59003625	59003644	dmr	2	0.311429	OMA1:intron
chr1	60317473	60317664	dmr	3	0.288807	HOOK1:intron

chr1	61704899	61704938	dmr	1	0.260943	NFIA:intron
chr1	61737927	61737978	dmr	1	0.226016	NFIA:intron
chr1	63058714	63058715	dmr	1	0.342432	DOCK7:intron
chr1	63128766	63128989	dmr	3	0.267032	DOCK7:CDS:2
chr1	68310225	68310408	dmr	2	0.314199	LOC100289178:intron
chr1	68325596	68325651	dmr	2	0.372451	LOC100289178:intron
chr1	68955171	68955276	dmr	2	0.327847	DEPDC1:CDS:3
chr1	70478692	70478877	dmr	3	0.281626	LRRC7:CDS:11
chr1	71321220	71321234	dmr	3	0.359628	PTGER3:intron
chr1	71534966	71535040	dmr	2	0.186862	ZRANB2:CDS:8
chr1	72540098	72540268	dmr	1	0.266067	NEGR1:intron
chr1	74662856	74662930	dmr	1	0.197648	FPGT:promoter,FPGT-TNNI3K:promoter
chr1	76621623	76621685	dmr	1	0.290476	ST6GALNAC3:intron
chr1	77629530	77629565	dmr	2	0.276471	PIGK:CDS:6
chr1	78164646	78164647	dmr	1	0.354167	USP33:intron
chr1	78339713	78339901	dmr	1	0.282849	FAM73A:intron
chr1	78409261	78409546	dmr	3	0.270378	NEXN:3UTR
chr1	82279817	82279818	dmr	1	0.441077	LPHN2:intron
chr1	82396988	82397154	dmr	1	0.242892	LPHN2:intron
chr1	86159953	86159954	dmr	1	0.353175	ZNHIT6:intron
chr1	86261066	86261220	dmr	2	0.3401	COL24A1:intron
chr1	89457268	89457565	dmr	4	-0.395997	RBMXL1:intron,CCBL2:intron
chr1	90312540	90312696	dmr	3	0.262198	LRRC8D:intron
chr1	91476874	91477100	dmr	3	0.271658	ZNF644:intron

chr1	93172105	93172238	dmr	2	0.361004	EVI5:intron
chr1	97236283	97236367	dmr	3	0.312745	PTBP2:CDS:5
chr1	97614364	97614365	dmr	1	0.354255	DPYD:intron
chr1	97666839	97666840	dmr	1	0.3125	DPYD:intron
chr1	97728021	97728022	dmr	1	0.423219	DPYD:intron
chr1	98230334	98230371	dmr	1	0.250754	DPYD:intron
chr1	98242508	98242557	dmr	3	0.313531	DPYD:intron
chr1	98260094	98260095	dmr	1	0.268293	DPYD:intron
chr1	98690171	98690364	dmr	3	0.293318	LOC729987:intron
chr1	103362096	103362107	dmr	2	0.339593	COL11A1:intron
chr1	107708892	107709041	dmr	3	0.344241	NTNG1:intron
chr1	109947664	109947858	dmr	2	0.355396	PSMA5:intron
chr1	111504388	111504481	dmr	2	0.315741	LRIF1:intron
chr1	113986086	113986266	dmr	2	0.292453	MAGI3:intron
chr1	114090051	114090179	dmr	1	0.228214	MAGI3:intron
chr1	115030709	115030876	dmr	4	0.320393	TRIM33:intron
chr1	116230796	116230882	dmr	2	0.411111	VANGL1:intron
chr1	117954829	117955015	dmr	3	0.283503	MAN1A2:intron
chr1	118407412	118407507	dmr	3	0.361763	GDAP2:3UTR
chr1	118478166	118478355	dmr	3	0.326943	WDR3:intron
chr1	120519903	120519904	dmr	1	0.185185	NOTCH2:intron
chr1	144990677	144990678	dmr	1	0.204949	PDE4DIP:intron,FLJ39739:intron,LOC728855:intron,PPIAL4B:intron,PPIAL4A:intron
chr1	145241182	145241227	dmr	2	0.221322	NOTCH2NL:intron,FLJ39739:intron,LOC728855:intron,PPIAL4B:intron,PPIA

						L4A:intron
chr1	162472869	162472917	dmr	1	0.180025	UHMK1:intron
chr1	169109100	169109247	dmr	3	0.346001	NME7:intron
chr1	169777440	169777448	dmr	2	0.377049	C1orf112:intron
chr1	169942822	169942997	dmr	3	0.295231	KIFAP3:intron
chr1	171813076	171813077	dmr	1	0.403226	DNM3:intron
chr1	172117349	172117350	dmr	1	0.35101	DNM3:intron
chr1	172118269	172118303	dmr	1	0.282458	DNM3:intron
chr1	172549106	172549167	dmr	3	0.28529	C1orf9:intron
chr1	175923526	175923641	dmr	2	0.399076	RFWD2:intron
chr1	175978668	175978678	dmr	2	0.346154	RFWD2:intron
chr1	176166821	176167018	dmr	3	0.305772	RFWD2:intron
chr1	178241304	178241431	dmr	2	0.278413	RASAL2:intron
chr1	178353971	178354126	dmr	3	0.266376	RASAL2:intron
chr1	178858406	178858577	dmr	2	0.239123	RALGPS2:intron
chr1	180010605	180010606	dmr	1	0.413665	CEP350:intron
chr1	183637224	183637225	dmr	1	0.571429	RGL1:intron
chr1	184702351	184702505	dmr	3	0.377004	EDEM3:intron
chr1	184908014	184908089	dmr	3	0.44089	FAM129A:intron
chr1	185018879	185018880	dmr	1	0.444444	RNF2:intron
chr1	186155241	186155310	dmr	1	0.182877	HMCN1:intron,MIR548F1:intron
chr1	186302107	186302108	dmr	1	0.326788	TPR:intron,MIR548F1:intron
chr1	186312533	186312716	dmr	2	0.271021	TPR:CDS:27
chr1	190143540	190143541	dmr	1	0.545455	FAM5C:intron

chr1	192606270	192606366	dmr	3	0.345938	RGS13:promoter
chr1	192997806	192998012	dmr	5	0.319251	UCHL5:intron
chr1	196365961	196366102	dmr	3	0.280713	KCNT2:intron
chr1	196437443	196437444	dmr	1	0.340351	KCNT2:intron
chr1	197147551	197147664	dmr	3	0.325262	ZBTB41:CDS:6
chr1	197608774	197608809	dmr	3	0.402718	DENND1B:intron
chr1	198273098	198273099	dmr	1	0.312937	NEK7:intron
chr1	198833507	198833508	dmr	1	0.267316	LOC100131234:intron
chr1	198900868	198901032	dmr	2	0.256372	LOC100131234:intron
chr1	202731475	202731607	dmr	2	0.27416	KDM5B:intron
chr1	212530530	212530648	dmr	3	0.354975	PPP2R5A:CDS:11
chr1	216495480	216495715	dmr	3	0.371871	USH2A:intron
chr1	216724829	216724924	dmr	1	0.300447	ESRRG:intron
chr1	217644330	217644331	dmr	1	0.371053	GPATCH2:intron
chr1	217774661	217774694	dmr	1	0.232323	GPATCH2:intron
chr1	220196023	220196193	dmr	2	0.271275	EPRS:intron,RNU5F-1:intron
chr1	222747739	222747861	dmr	2	0.313725	TAF1A:intron
chr1	223173786	223173787	dmr	1	0.432692	DISP1:intron
chr1	224599128	224599246	dmr	2	0.295915	WDR26:CDS:7
chr1	225592386	225592387	dmr	1	0.357143	LBR:CDS:11
chr1	225682541	225682591	dmr	1	0.24055	ENAH:3UTR
chr1	226434673	226434842	dmr	3	0.269585	LIN9:intron
chr1	230822133	230822134	dmr	1	0.34632	COG2:intron
chr1	233400534	233400535	dmr	1	0.342105	PCNXL2:intron

chr1	235335955	235336150	dmr	4	0.220324	ARID4B:CDS:22
chr1	237585645	237585811	dmr	3	0.306275	RYR2:intron
chr1	243358689	243358890	dmr	3	0.266174	CEP170:intron
chr1	243535563	243535564	dmr	1	0.27295	SDCCAG8:intron
chr1	243586805	243586852	dmr	1	0.29798	SDCCAG8:intron
chr1	245135019	245135180	dmr	4	-0.247271	EFCAB2:intron
chr1	245354997	245355133	dmr	1	0.281977	KIF26B:intron
chr1	246540054	246540176	dmr	3	0.272494	SMYD3:intron
chr1	247002354	247002525	dmr	3	0.307069	AHCTF1:3UTR
chr10	251032	251073	dmr	2	0.343284	ZMYND11:intron
chr10	282322	282369	dmr	1	0.328283	ZMYND11:intron
chr10	11607060	11607227	dmr	3	0.327878	USP6NL:intron
chr10	16734724	16734725	dmr	1	0.483333	RSU1:intron
chr10	16773682	16773683	dmr	1	0.3125	RSU1:intron
chr10	17202031	17202230	dmr	2	0.349199	TRDMT1:intron
chr10	17636320	17636321	dmr	1	0.363739	PTPLA:CDS:6
chr10	21418802	21418803	dmr	1	0.367857	NEBL:intron
chr10	21979413	21979580	dmr	4	0.308317	MLLT10:intron
chr10	27047520	27047718	dmr	3	0.270566	ABI1:intron
chr10	27518763	27518764	dmr	1	0.266667	ACBD5:intron
chr10	31627632	31627637	dmr	2	0.284615	ZEB1:intron
chr10	32581510	32581568	dmr	4	0.337746	EPC1:CDS:5
chr10	50712219	50712401	dmr	3	0.295491	ERCC6:intron,FAM21B:intron
chr10	52754793	52754989	dmr	2	0.314367	PRKG1:intron

chr10	54008234	54008408	dmr	2	0.329317	PRKG1:intron
chr10	60412366	60412374	dmr	2	0.325175	BICC1:intron
chr10	63981225	63981226	dmr	1	0.326667	RTKN2:intron
chr10	70549577	70549638	dmr	2	0.342895	CCAR1:CDS:23
chr10	70766653	70766694	dmr	1	0.375	KIAA1279:intron
chr10	74526713	74526714	dmr	1	0.31875	MCU:intron
chr10	75801768	75801838	dmr	2	0.355426	VCL:intron
chr10	76023503	76023592	dmr	2	0.395664	ADK:intron
chr10	76068133	76068278	dmr	3	0.306088	ADK:intron
chr10	78083149	78083306	dmr	2	0.297529	C10orf11:intron
chr10	88554389	88554390	dmr	1	0.324324	BMPR1A:intron
chr10	93942968	93942969	dmr	1	0.287718	CPEB3:intron
chr10	94110759	94110858	dmr	1	0.233083	MARCH5:CDS:6
chr10	96336484	96336670	dmr	2	0.232471	HELLS:CDS:10
chr10	96344683	96344879	dmr	3	0.32134	HELLS:intron
chr10	97434189	97434262	dmr	3	0.373835	TCTN3:intron
chr10	114326154	114326298	dmr	3	0.338088	VTI1A:intron
chr10	114422183	114422184	dmr	1	0.342742	VTI1A:intron
chr10	114814473	114814516	dmr	2	0.337365	TCF7L2:intron
chr10	115606610	115606734	dmr	1	0.209727	DCLRE1A:intron
chr10	116731239	116731240	dmr	1	0.235294	TRUB1:intron
chr10	126662405	126662406	dmr	1	0.245588	ZRANB1:intron
chr10	129037887	129038060	dmr	4	0.284069	DOCK1:intron
chr11	9462591	9462701	dmr	2	0.363871	IPO7:intron

chr11	9889988	9890021	dmr	2	0.292683	SBF2:intron
chr11	10226767	10226829	dmr	1	0.269398	SBF2:intron
chr11	10874261	10874262	dmr	1	0.311364	ZBED5:ncexon:3
chr11	11934436	11934564	dmr	1	0.248793	USP47:intron
chr11	13433197	13433198	dmr	1	0.341463	BTBD10:intron
chr11	16239493	16239663	dmr	3	0.318134	SOX6:intron
chr11	17141871	17141872	dmr	1	0.306713	PIK3C2A:intron
chr11	20431720	20431721	dmr	1	0.347967	PRMT3:intron
chr11	24773539	24773730	dmr	3	0.347356	LUZP2:intron
chr11	28218675	28218777	dmr	3	0.303883	METTL15:intron
chr11	28246036	28246478	dmr	5	0.344889	METTL15:intron
chr11	28261820	28262006	dmr	3	0.29586	METTL15:intron
chr11	28285151	28285152	dmr	1	0.302326	METTL15:intron
chr11	28343407	28343408	dmr	1	0.321429	METTL15:intron
chr11	31511368	31511545	dmr	4	0.329058	IMMP1L:intron
chr11	31661078	31661248	dmr	3	0.345307	ELP4:intron
chr11	32990944	32991051	dmr	2	0.285621	QSER1:intron
chr11	33133123	33133235	dmr	3	0.29853	CSTF3:intron
chr11	34953896	34954057	dmr	2	0.252301	PDHX:intron
chr11	34971662	34971663	dmr	1	0.4	PDHX:intron
chr11	43850422	43850455	dmr	3	0.334915	HSD17B12:intron
chr11	44325657	44325865	dmr	4	0.509895	ALX4:intron
chr11	47046838	47046907	dmr	3	0.308642	C11orf49:intron
chr11	63371515	63371720	dmr	3	0.355789	PLA2G16:intron

chr11	67947752	67947817	dmr	2	0.309559	SUV420H1:intron
chr11	75809055	75809144	dmr	3	0.28352	UVRAG:intron
chr11	77450626	77450780	dmr	3	0.287146	RSF1:intron
chr11	78238008	78238104	dmr	3	0.249824	NARS2:intron
chr11	86766601	86766602	dmr	1	0.287879	TMEM135:intron
chr11	95645812	95645813	dmr	1	0.333333	MTMR2:intron
chr11	100730292	100730299	dmr	1	0.279266	ARHGAP42:CDS:4
chr11	102208176	102208177	dmr	1	0.457143	BIRC3:3UTR
chr11	103139282	103139291	dmr	2	0.44251	DYNC2H1:intron
chr11	107321318	107321319	dmr	1	0.230769	CWF19L2:intron
chr11	107960165	107960166	dmr	1	0.368421	CUL5:intron
chr11	108155278	108155488	dmr	3	0.299273	ATM:intron
chr11	108163916	108164101	dmr	2	0.335065	ATM:CDS:30
chr11	108597944	108598121	dmr	3	0.29858	DDX10:intron
chr11	108639512	108639547	dmr	2	0.314892	DDX10:intron
chr11	110144519	110144624	dmr	3	0.32545	RDX:intron
chr11	111909781	111909819	dmr	3	0.254212	DLAT:intron
chr11	120319762	120319893	dmr	2	0.173559	ARHGEF12:CDS:21
chr11	130174147	130174198	dmr	2	0.465672	ZBTB44:intron
chr12	940174	940205	dmr	1	0.268421	WNK1:intron
chr12	1188037	1188363	dmr	3	0.312692	ERC1:intron
chr12	1871722	1871731	dmr	2	0.288961	ADIPOR2:intron
chr12	10369598	10369631	dmr	2	0.422222	GABARAPL1:intron
chr12	14613165	14613201	dmr	2	0.308653	ATF7IP:intron

chr12	18459797	18459963	dmr	3	0.311677	PIK3C2G:intron
chr12	18862900	18862997	dmr	3	0.339758	PLCZ1:intron
chr12	19499059	19499240	dmr	2	0.304832	PLEKHA5:intron
chr12	20571645	20571646	dmr	1	0.375458	PDE3A:intron
chr12	22624760	22624761	dmr	1	0.333333	KIAA0528:intron
chr12	22658485	22658618	dmr	1	0.290148	KIAA0528:intron
chr12	22659658	22659702	dmr	2	0.430769	KIAA0528:CDS:9
chr12	24477322	24477476	dmr	2	0.294925	SOX5:intron
chr12	28424976	28425008	dmr	3	0.466725	CCDC91:intron
chr12	28454089	28454230	dmr	2	0.372764	CCDC91:intron
chr12	29520174	29520329	dmr	3	0.316362	ERGIC2:intron
chr12	30810824	30810825	dmr	1	0.32	IPO8:intron
chr12	30885658	30885810	dmr	3	0.271824	CAPRIN2:intron
chr12	39702586	39702766	dmr	3	0.342929	KIF21A:intron
chr12	44468215	44468385	dmr	3	0.321929	TMEM117:intron
chr12	46637538	46637574	dmr	1	0.244721	SLC38A1:promoter
chr12	48460019	48460026	dmr	2	0.338827	SENP1:intron
chr12	62718777	62718960	dmr	3	0.29373	USP15:intron
chr12	65033116	65033117	dmr	1	0.297619	RASSF3:intron
chr12	69236269	69236270	dmr	1	0.37931	MDM2:3UTR
chr12	72050154	72050331	dmr	3	0.312401	ZFC3H1:intron
chr12	75731416	75731648	dmr	4	0.305015	GLIPR1L1:intron
chr12	75795251	75795351	dmr	2	0.356463	GLIPR1L2:intron
chr12	77448975	77449040	dmr	2	0.359375	E2F7:intron

chr12	79370466	79370664	dmr	2	0.277498	SYT1:promoter
chr12	80184367	80184434	dmr	1	0.267654	PPP1R12A:intron
chr12	80200983	80201169	dmr	2	0.220163	PPP1R12A:CDS:11
chr12	80324833	80325024	dmr	4	0.338727	PPP1R12A:intron
chr12	81024364	81024564	dmr	2	0.342931	PTPRQ:intron
chr12	81302598	81302787	dmr	3	0.304492	LIN7A:intron
chr12	86717419	86717590	dmr	3	0.290585	MGAT4C:intron
chr12	86876563	86876564	dmr	1	0.300785	MGAT4C:intron
chr12	87225991	87225992	dmr	1	0.25	MGAT4C:intron
chr12	88497081	88497082	dmr	1	0.27619	CEP290:intron
chr12	88969713	88969913	dmr	3	0.31617	KITLG:intron
chr12	94091332	94091340	dmr	1	0.308362	CRADD:intron
chr12	95625479	95625589	dmr	3	0.310714	VEZT:intron
chr12	95660168	95660382	dmr	4	0.324206	VEZT:CDS:5
chr12	95669575	95669744	dmr	3	0.29558	VEZT:intron
chr12	96764423	96764677	dmr	3	0.263889	CDK17:intron
chr12	99099687	99099733	dmr	3	0.434211	APAF1:intron
chr12	100732033	100732163	dmr	2	0.313865	SCYL2:intron
chr12	101016041	101016125	dmr	1	0.227385	GAS2L3:CDS:7
chr12	102151035	102151060	dmr	3	0.299065	GNPTAB:CDS:18
chr12	102199614	102199800	dmr	4	0.272657	GNPTAB:intron
chr12	102505093	102505236	dmr	3	0.299167	NUP37:intron
chr12	105440677	105440719	dmr	1	0.281981	ALDH1L2:CDS:14
chr12	105540785	105541004	dmr	3	0.184712	KIAA1033:CDS:24

chr12	109073496	109073667	dmr	3	0.248055	CORO1C:intron
chr12	111992114	111992247	dmr	3	0.270865	ATXN2:intron
chr12	120274459	120274465	dmr	1	0.258065	CIT:intron
chr12	124203180	124203181	dmr	1	0.241379	ATP6V0A2:CDS:2
chr12	124242215	124242316	dmr	3	-0.297466	ATP6V0A2:intron
chr12	133618338	133618432	dmr	3	0.208333	ZNF84:intron
chr12	133757479	133757755	dmr	2	0.206664	ZNF268:promoter
chr13	20576545	20576594	dmr	1	0.359597	ZMYM2:intron
chr13	21429256	21429257	dmr	1	0.318182	XPO4:intron
chr13	26975256	26975257	dmr	1	0.205051	CDK8:intron
chr13	27660521	27660656	dmr	3	0.363553	USP12:intron
chr13	27731513	27731574	dmr	2	0.301198	USP12:intron
chr13	28228180	28228181	dmr	1	0.346154	POLR1D:intron
chr13	32920977	32921146	dmr	4	0.291112	BRCA2:CDS:12
chr13	33084269	33084316	dmr	2	0.394195	N4BP2L2:intron
chr13	33719436	33719528	dmr	3	0.222222	STARD13:intron
chr13	34127366	34127551	dmr	2	0.364274	STARD13:intron
chr13	35531960	35532099	dmr	3	0.307384	NBEA:intron
chr13	35814194	35814269	dmr	1	0.241841	NBEA:intron
chr13	37605646	37605647	dmr	1	0.294077	FAM48A:intron
chr13	41906634	41906635	dmr	1	0.366667	NAA16:intron
chr13	42279213	42279251	dmr	2	0.273684	KIAA0564:intron
chr13	42867247	42867368	dmr	3	0.319195	AKAP11:intron
chr13	46617683	46617820	dmr	3	0.293485	ZC3H13:intron

chr13	48909182	48909208	dmr	2	0.272683	RB1:intron
chr13	48941500	48941653	dmr	3	0.30303	RB1:CDS:10
chr13	49748670	49748671	dmr	1	0.30031	FNDC3A:CDS:10
chr13	49923363	49923499	dmr	3	0.460317	CAB39L:intron
chr13	53216582	53216743	dmr	3	0.224129	HNRNPA1L2:promoter
chr13	53216582	53216743	dmr	3	0.224129	HNRNPA1L2:promoter,HNRNPA1L2:5UTR
chr13	60552627	60552628	dmr	1	0.307018	DIAPH3:intron
chr13	60689749	60689750	dmr	1	0.324324	DIAPH3:intron
chr13	60700599	60700750	dmr	2	0.216633	DIAPH3:intron
chr13	61120993	61121052	dmr	3	0.284927	TDRD3:intron
chr13	67219603	67219604	dmr	1	0.3	PCDH9:intron
chr13	67250005	67250167	dmr	5	-0.528061	PCDH9:intron
chr13	67574927	67574928	dmr	1	0.379345	PCDH9:intron
chr13	67731216	67731410	dmr	4	0.274336	PCDH9:intron
chr13	73325784	73325890	dmr	2	0.248839	BORA:intron
chr13	77702145	77702209	dmr	3	0.430446	MYCBP2:intron
chr13	77755724	77755895	dmr	1	0.309514	MYCBP2:CDS:33
chr13	77847766	77847895	dmr	3	0.360617	MYCBP2:CDS:5
chr13	93175157	93175473	dmr	3	0.37157	GPC5:intron
chr13	93963058	93963120	dmr	1	0.264039	GPC6:intron
chr13	93973073	93973178	dmr	2	0.293007	GPC6:intron
chr13	94070859	94071097	dmr	4	0.342451	GPC6:intron
chr13	95696069	95696070	dmr	1	0.324324	ABCC4:intron
chr13	98882116	98882309	dmr	2	0.277228	FARP1:intron

chr13	100916123	100916426	dmr	2	0.347908	PCCA:intron
chr13	101051267	101051544	dmr	3	0.334892	PCCA:intron
chr13	101776553	101776554	dmr	1	0.5	NALCN:intron
chr13	101974032	101974218	dmr	3	0.32368	NALCN:intron
chr13	103520470	103520607	dmr	2	0.299046	ERCC5:CDS:12,BIVM-ERCC5:CDS:20
chr13	108095249	108095250	dmr	1	0.274428	FAM155A:intron
chr13	108886160	108886314	dmr	3	0.324101	ABHD13:ncexon:2
chr13	109756159	109756355	dmr	2	0.281933	MYO16:intron
chr14	32588383	32588384	dmr	1	0.229365	ARHGAP5:intron
chr14	33114446	33114677	dmr	3	0.29578	AKAP6:intron
chr14	34141409	34141410	dmr	1	0.24	NPAS3:intron
chr14	36244915	36244916	dmr	1	0.296228	RALGAPA1:CDS:2
chr14	37794422	37794477	dmr	3	0.336088	MIPOL1:intron
chr14	39514390	39514391	dmr	1	0.318182	SEC23A:CDS:15
chr14	39773832	39774009	dmr	1	0.243309	CTAGE5:intron
chr14	39776208	39776221	dmr	1	0.233949	CTAGE5:intron
chr14	39806241	39806350	dmr	1	0.25487	CTAGE5:intron
chr14	42229368	42229405	dmr	2	0.326923	LRFN5:intron
chr14	45472465	45472562	dmr	3	0.272059	FAM179B:intron
chr14	50904786	50904819	dmr	3	0.314362	MAP4K5:intron
chr14	50915236	50915363	dmr	3	0.385375	MAP4K5:intron
chr14	57706025	57706026	dmr	1	0.333333	EXOC5:intron
chr14	61363954	61363955	dmr	1	0.25	MNAT1:intron
chr14	65983539	65983591	dmr	3	0.368222	FUT8:intron

chr14	67002387	67002530	dmr	4	0.295517	GPHN:intron
chr14	67267059	67267077	dmr	3	0.288506	GPHN:intron
chr14	68875399	68875400	dmr	1	0.312069	RAD51B:intron
chr14	71996223	71996224	dmr	1	0.25515	SIPA1L1:intron
chr14	73572942	73572943	dmr	1	0.578947	RBM25:CDS:11
chr14	81388929	81388930	dmr	1	0.347902	CEP128:intron
chr14	81963528	81963529	dmr	1	0.261905	SEL1L:intron
chr14	88971789	88971790	dmr	1	0.386202	PTPN21:intron
chr14	92438419	92438584	dmr	2	0.268404	TRIP11:intron
chr14	97001620	97001621	dmr	1	0.343605	PAPOLA:3UTR
chr14	97004290	97004375	dmr	2	0.242325	PAPOLA:intron
chr15	24724872	24724873	dmr	1	0.4375	MIR4509-3:intron,MIR4509-2:intron,MIR4509-1:intron
chr15	25200003	25200253	dmr	4	-0.515487	SNRPN:promoter,SNURF:promoter
chr15	25200003	25200253	dmr	4	-0.515487	SNRPN:promoter,SNURF:promoter,SNRPN:5UTR,SNURF:5UTR
chr15	30036794	30036974	dmr	2	0.25565	TJP1:intron
chr15	31203203	31203418	dmr	4	0.282651	FAN1:3UTR
chr15	36131131	36131296	dmr	4	0.489838	LOC100507466:intron
chr15	36973776	36973929	dmr	2	0.285227	C15orf41:intron
chr15	37389383	37389395	dmr	1	-0.414286	MEIS2:intron
chr15	38756432	38756433	dmr	1	0.266667	FAM98B:intron
chr15	40920781	40920983	dmr	4	0.25903	CASC5:CDS:12
chr15	44685090	44685091	dmr	1	0.287879	CASC4:intron
chr15	44745204	44745205	dmr	1	0.285714	CTDSPL2:intron
chr15	45796598	45796764	dmr	3	0.316236	SLC30A4:intron

chr15	49335356	49335533	dmr	2	0.359726	SECISBP2L:intron
chr15	49443724	49443744	dmr	2	0.37688	COPS2:intron
chr15	50315483	50315528	dmr	3	0.482385	ATP8B4:intron
chr15	50885672	50885829	dmr	2	0.283106	TRPM7:CDS:25
chr15	50927095	50927289	dmr	2	0.203632	TRPM7:intron
chr15	56226732	56226733	dmr	1	0.323684	NEDD4:intron
chr15	56462265	56462303	dmr	2	0.295267	RFX7:intron
chr15	56488627	56488628	dmr	1	0.251232	RFX7:intron
chr15	56686783	56686925	dmr	1	0.225443	TEX9:CDS:9
chr15	56935175	56935390	dmr	4	0.292695	ZNF280D:CDS:17
chr15	56996745	56996746	dmr	1	0.214286	ZNF280D:promoter
chr15	57402065	57402219	dmr	3	0.342696	TCF12:intron
chr15	59191462	59191463	dmr	1	0.371429	SLTM:intron
chr15	59342618	59342619	dmr	1	0.258065	RNF111:intron
chr15	59491743	59491923	dmr	2	0.292689	MYO1E:intron
chr15	64088660	64088689	dmr	2	0.319167	HERC1:intron
chr15	71220491	71220650	dmr	3	0.28184	LRRC49:intron
chr15	72169604	72169611	dmr	1	0.217669	MYO9A:intron
chr15	76634307	76634694	dmr	6	-0.352876	ISL2:3UTR
chr15	76705226	76705253	dmr	2	0.325594	SCAPER:intron
chr15	86100493	86100494	dmr	1	0.357143	AKAP13:intron
chr15	99784829	99784830	dmr	1	0.336898	TTC23:intron
chr15	100674365	100674366	dmr	1	0.571429	ADAMTS17:intron
chr16	7087134	7087135	dmr	1	-0.503676	RBFOX1:intron

chr16	14307140	14307149	dmr	2	0.313549	MKL2:intron
chr16	24765563	24765726	dmr	3	0.300952	TNRC6A:intron
chr16	56466591	56466615	dmr	1	0.21866	NUDT21:3UTR
chr16	67078249	67078250	dmr	1	0.34188	CBFB:intron
chr16	69666325	69666515	dmr	3	0.249649	NFAT5:intron
chr16	79006762	79006898	dmr	3	-0.361539	WWOX:intron
chr16	80580459	80580581	dmr	1	0.165404	DYNLRB2:intron
chr16	87431334	87431335	dmr	1	0.257143	MAP1LC3B:intron
chr17	8472648	8472716	dmr	2	0.243111	MYH10:intron
chr17	12007035	12007152	dmr	2	0.349528	MAP2K4:intron
chr17	16034860	16034971	dmr	2	0.273183	NCOR1:intron
chr17	20178209	20178349	dmr	2	0.31746	SPECC1:intron
chr17	28055296	28055421	dmr	1	0.316303	SSH2:intron
chr17	40422339	40422340	dmr	1	-0.344828	STAT5B:intron
chr17	54934928	54935078	dmr	1	0.22119	DGKE:intron
chr17	56801292	56801489	dmr	2	0.290278	RAD51C:CDS:7
chr17	57158491	57158532	dmr	3	0.296982	TRIM37:CDS:6
chr17	58911036	58911041	dmr	1	0.288576	BCAS3:intron
chr17	59899440	59899567	dmr	2	0.292488	BRIP1:intron
chr17	60053645	60053710	dmr	1	0.369907	MED13:intron
chr17	60721217	60721218	dmr	1	0.30303	MRC2:intron
chr17	61213122	61213243	dmr	4	0.329623	TANC2:intron,MIR548W:intron
chr17	62487030	62487031	dmr	1	0.3	POLG2:CDS:4
chr17	62646187	62646241	dmr	1	0.291903	SMURF2:intron

chr17	66937388	66937547	dmr	2	0.268982	ABCA8:intron
chr17	67121612	67121613	dmr	1	0.268519	ABCA6:intron
chr17	78596834	78596996	dmr	2	0.264629	RPTOR:intron
chr17	80049289	80049589	dmr	3	0.531924	FASN:CDS:8
chr17	80970789	80971075	dmr	1	0.375652	B3GNTL1:intron
chr18	2666910	2667090	dmr	3	0.212565	SMCHD1:CDS:3
chr18	2931672	2931689	dmr	2	0.252747	LPIN2:intron
chr18	9820956	9821104	dmr	3	0.451215	RAB31:intron
chr18	10918580	10918729	dmr	2	0.366924	PIEZO2:intron
chr18	12697276	12697281	dmr	1	0.238095	CEP76:CDS:5
chr18	13575768	13575777	dmr	1	0.341837	C18orf1:intron
chr18	18549466	18549548	dmr	3	0.302001	ROCK1:intron
chr18	19141158	19141159	dmr	1	0.26412	ESCO1:intron
chr18	19371366	19371418	dmr	1	0.247527	MIB1:CDS:7
chr18	25614083	25614254	dmr	2	0.308333	CDH2:intron
chr18	30529031	30529173	dmr	2	0.356758	C18orf34:intron
chr18	31259947	31260172	dmr	3	0.35537	ASXL3:intron
chr18	34343163	34343507	dmr	3	0.385086	FHOD3:intron
chr18	37268347	37268406	dmr	2	0.365495	LOC647946:intron
chr18	46210961	46210962	dmr	1	0.314286	CTIF:intron
chr18	46749226	46749409	dmr	3	0.284143	DYM:intron
chr18	46785913	46785914	dmr	1	0.290143	DYM:intron
chr18	46953097	46953098	dmr	1	0.5	DYM:intron
chr18	54539731	54539732	dmr	1	0.333333	WDR7:intron

chr18	59892807	59892808	dmr	1	0.318083	KIAA1468:intron
chr18	59933525	59933558	dmr	2	0.415932	KIAA1468:intron
chr18	60597400	60597401	dmr	1	0.377358	PHLPP1:intron
chr18	61594202	61594203	dmr	1	0.392308	SERPINB10:intron
chr18	65492493	65492494	dmr	1	0.423188	LOC643542:intron
chr18	65545577	65545604	dmr	2	0.284324	LOC643542:intron
chr18	66355288	66355289	dmr	1	0.260163	TMX3:intron
chr18	66643582	66643783	dmr	2	0.306616	CCDC102B:intron
chr18	72396445	72396569	dmr	2	0.219655	ZNF407:intron
chr18	72769089	72769090	dmr	1	0.345644	ZNF407:intron
chr18	76886293	76886404	dmr	2	0.276335	ATP9B:CDS:5
chr19	6750376	6750570	dmr	1	-0.326923	TRIP10:CDS:12,TRIP10:CDS:13
chr19	21609515	21609623	dmr	3	0.375816	ZNF493:3UTR
chr19	32959665	32959836	dmr	4	0.309903	DPY19L3:CDS:15
chr19	37129014	37129075	dmr	1	0.17619	ZNF461:3UTR
chr19	38134546	38134597	dmr	3	0.329987	ZFP30:intron
chr19	44515556	44515669	dmr	2	0.263889	ZNF230:CDS:4
chr19	52510332	52510333	dmr	1	0.330714	ZNF615:intron
chr19	52547463	52547480	dmr	2	0.288462	ZNF432:intron
chr19	56921550	56921563	dmr	1	0.226807	ZNF583:intron
chr2	8909694	8909883	dmr	3	0.254014	KIDINS220:intron
chr2	8923709	8923861	dmr	2	0.37561	KIDINS220:intron
chr2	8964272	8964374	dmr	2	0.350183	KIDINS220:intron
chr2	9140846	9141023	dmr	2	0.311228	MBOAT2:intron

chr2	9736436	9736538	dmr	1	0.265501	YWHAQ:intron
chr2	15607468	15607502	dmr	3	0.256692	NBAS:CDS:19
chr2	17871955	17871956	dmr	1	0.268726	SMC6:intron
chr2	17872059	17872155	dmr	1	0.281894	SMC6:intron
chr2	17897188	17897333	dmr	4	0.295945	SMC6:intron
chr2	24919927	24919962	dmr	3	0.355556	NCOA1:intron
chr2	36770622	36770772	dmr	2	0.287047	CRIM1:intron
chr2	37132800	37132832	dmr	2	0.34232	STRN:intron
chr2	37443484	37443485	dmr	1	0.315897	CEBPZ:CDS:7
chr2	37535226	37535227	dmr	1	0.314286	PRKD3:intron
chr2	39554216	39554365	dmr	3	0.236312	MAP4K3:intron
chr2	39983877	39983913	dmr	1	0.375	THUMPD2:intron
chr2	43757505	43757649	dmr	2	0.273587	THADA:intron
chr2	43921975	43922115	dmr	3	0.324114	PLEKHH2:intron
chr2	45806897	45807036	dmr	3	0.338579	SRBD1:CDS:6
chr2	48764369	48764370	dmr	1	0.290323	STON1:intron,STON1-GTF2A1L:intron
chr2	54130971	54131093	dmr	3	0.320683	PSME4:intron
chr2	54504206	54504207	dmr	1	0.236842	ACYP2:intron
chr2	54807105	54807286	dmr	1	0.379682	SPTBN1:intron
chr2	55867765	55867798	dmr	1	0.325955	PNPT1:CDS:26
chr2	55875342	55875533	dmr	2	0.360152	PNPT1:intron
chr2	58391841	58392028	dmr	3	0.280899	FANCL:intron
chr2	61147182	61147243	dmr	1	0.315579	REL:CDS:8
chr2	61505486	61505705	dmr	4	0.267994	USP34:CDS:40

chr2	61516421	61516448	dmr	2	0.36314	USP34:intron
chr2	63191092	63191158	dmr	2	0.267716	EHBP1:intron
chr2	63713058	63713236	dmr	2	0.392983	WDPCP:intron
chr2	64805466	64805467	dmr	1	0.241379	AFTPH:intron
chr2	65564899	65565078	dmr	2	0.369149	SPRED2:intron
chr2	69279185	69279186	dmr	1	0.279762	ANTXR1:intron
chr2	69601202	69601212	dmr	2	0.246863	GFPT1:CDS:2
chr2	71645944	71646151	dmr	2	0.32322	ZNF638:intron
chr2	88124816	88124926	dmr	1	-0.465789	RGPD2:intron,RGPD1:intron
chr2	95537491	95537568	dmr	2	-0.675	TEKT4:CDS:1
chr2	99938152	99938240	dmr	3	0.295597	TXNDC9:intron
chr2	100415149	100415255	dmr	2	0.331309	AFF3:intron
chr2	112874967	112874968	dmr	1	0.301136	TMEM87B:3UTR
chr2	113057458	113057592	dmr	1	0.330224	ZC3H6:CDS:2
chr2	120706432	120706433	dmr	1	0.404558	PTPN4:intron
chr2	130691712	130691977	dmr	1	-0.349752	LOC389033:ncexon:1
chr2	135896641	135896642	dmr	1	0.333333	RAB3GAP1:intron
chr2	136346533	136346634	dmr	3	0.317909	R3HDM1:intron
chr2	141898954	141898955	dmr	1	0.4375	LRP1B:intron
chr2	142604201	142604472	dmr	5	0.350689	LRP1B:intron
chr2	143986662	143986663	dmr	1	0.277778	ARHGAP15:intron
chr2	144213247	144213248	dmr	1	0.280357	ARHGAP15:intron
chr2	144911451	144911452	dmr	1	0.356522	GTDC1:intron
chr2	144934884	144935072	dmr	3	0.262677	GTDC1:intron

chr2	145243871	145244019	dmr	3	0.33312	ZEB2:intron
chr2	145786242	145786339	dmr	4	0.474321	DKFZp686O1327:intron
chr2	152298181	152298383	dmr	2	0.319332	RIF1:intron
chr2	152324697	152324698	dmr	1	0.307692	RIF1:intron
chr2	153213563	153213864	dmr	4	0.335858	FMNL2:intron
chr2	160382850	160382993	dmr	3	0.324514	BAZ2B:intron
chr2	160461463	160461466	dmr	2	0.818182	BAZ2B:intron
chr2	160602628	160602724	dmr	3	0.293693	MARCH7:intron
chr2	161346174	161346233	dmr	2	0.266674	RBMS1:intron
chr2	162055495	162055681	dmr	2	0.303797	TANK:intron
chr2	162186211	162186299	dmr	1	0.265537	PSMD14:intron
chr2	165798320	165798321	dmr	1	0.4	SLC38A11:intron
chr2	167277612	167277925	dmr	3	0.341881	SCN7A:CDS:18
chr2	169732592	169732593	dmr	1	0.403251	SPC25:CDS:4
chr2	169739665	169739666	dmr	1	0.266667	SPC25:intron
chr2	172300377	172300378	dmr	1	0.352087	DCAF17:intron
chr2	172582737	172582825	dmr	2	0.289986	DYNC1I2:CDS:9
chr2	172848233	172848249	dmr	2	0.31098	HAT1:CDS:11
chr2	175963638	175963639	dmr	1	0.357143	ATF2:intron
chr2	177141686	177141828	dmr	2	0.22861	MTX2:intron
chr2	180047699	180047900	dmr	3	0.39913	SESTD1:CDS:2
chr2	180819082	180819083	dmr	1	0.317587	CWC22:CDS:15
chr2	180850277	180850449	dmr	4	0.329339	CWC22:intron
chr2	183834246	183834247	dmr	1	0.275862	NCKAP1:intron

chr2	183849961	183849962	dmr	1	0.351351	NCKAP1:intron
chr2	183866180	183866344	dmr	3	0.396748	NCKAP1:intron
chr2	184026167	184026354	dmr	3	0.277911	NUP35:3UTR
chr2	188226621	188226622	dmr	1	0.28734	CALCRL:intron
chr2	188331910	188331980	dmr	2	0.317545	TFPI:intron
chr2	189453937	189454069	dmr	2	0.342694	GULP1:intron
chr2	191535067	191535068	dmr	1	0.279915	NAB1:CDS:2
chr2	192930995	192930996	dmr	1	0.321059	TMEFF2:intron
chr2	192944552	192944703	dmr	2	0.346667	TMEFF2:intron
chr2	196889798	196889799	dmr	1	0.392857	DNAH7:intron
chr2	197021423	197021424	dmr	1	0.514286	STK17B:intron
chr2	197431051	197431075	dmr	2	0.32815	HECW2:intron
chr2	197700593	197700787	dmr	3	0.292079	PGAP1:3UTR
chr2	197782184	197782287	dmr	1	0.362772	PGAP1:intron
chr2	198414864	198415087	dmr	3	0.251793	HSPE1-MOB4:CDS:8,MOB4:CDS:6
chr2	198694056	198694189	dmr	3	0.298916	PLCL1:intron
chr2	202326941	202327073	dmr	2	0.322186	STRADB:intron
chr2	203408416	203408503	dmr	2	0.311201	BMPR2:intron
chr2	203557346	203557422	dmr	3	0.326598	FAM117B:intron
chr2	203959274	203959275	dmr	1	0.245736	NBEAL1:intron
chr2	205561476	205561477	dmr	1	0.360256	PARD3B:intron
chr2	205722724	205722725	dmr	1	0.305897	PARD3B:intron
chr2	206002058	206002071	dmr	1	0.456017	PARD3B:intron
chr2	209116356	209116377	dmr	1	0.272683	IDH1:promoter

chr2	212500468	212500469	dmr	1	0.249474	ERBB4:intron
chr2	212507647	212507648	dmr	1	0.464286	ERBB4:intron
chr2	212995404	212995459	dmr	1	0.311912	ERBB4:intron
chr2	213137374	213137476	dmr	2	0.375213	ERBB4:intron
chr2	213894812	213894813	dmr	1	0.354167	IKZF2:intron
chr2	215627285	215627501	dmr	4	0.252005	BARD1:intron
chr2	216281791	216281792	dmr	1	0.37538	FN1:intron
chr2	216620670	216620680	dmr	2	-0.452588	LOC646324:intron
chr2	216623263	216623353	dmr	3	0.320513	LOC646324:intron
chr2	223787634	223787641	dmr	2	0.26238	ACSL3:intron
chr2	225370765	225370822	dmr	3	0.305789	CUL3:CDS:8
chr2	228403780	228403795	dmr	1	0.301075	AGFG1:intron
chr2	228748774	228748860	dmr	3	0.383228	WDR69:intron
chr2	232003429	232003623	dmr	4	0.276859	PSMD1:CDS:17
chr2	234420907	234420997	dmr	3	0.302108	USP40:intron
chr2	242035423	242035523	dmr	3	0.266148	MTERFD2:CDS:4
chr20	13605010	13605180	dmr	3	0.403873	TASP1:intron
chr20	13752050	13752051	dmr	1	0.343544	ESF1:CDS:5
chr20	14217176	14217274	dmr	1	0.257843	MACROD2:intron
chr20	14472009	14472142	dmr	3	0.304984	MACROD2:intron
chr20	14473552	14473751	dmr	3	0.377387	MACROD2:intron
chr20	17535346	17535471	dmr	2	0.375	BFSP1:intron
chr20	35696397	35696524	dmr	3	0.312362	RBL1:CDS:3
chr20	51601752	51601772	dmr	1	0.274704	TSHZ2:intron

chr20	57608516	57608660	dmr	3	0.293738	SLMO2:3UTR
chr21	17891029	17891077	dmr	3	0.345333	LINC00478:intron
chr21	18965928	18966132	dmr	3	0.241366	BTG3:3UTR
chr21	26326414	26326415	dmr	1	0.326491	LOC339622:intron
chr21	33647015	33647159	dmr	2	0.279289	MIS18A:CDS:2
chr21	34060657	34060781	dmr	2	0.225589	SYNJ1:CDS:6
chr21	37747450	37747525	dmr	3	0.264515	MORC3:CDS:17
chr21	38327884	38328077	dmr	3	0.332593	HLCS:intron
chr21	38802141	38802180	dmr	1	0.329992	DYRK1A:intron
chr21	40636016	40636189	dmr	3	0.269446	BRWD1:intron
chr21	44445955	44445978	dmr	3	-0.513333	PKNOX1:intron
chr21	47702232	47702262	dmr	3	0.317614	MCM3AP:intron
chr22	19496615	19496616	dmr	1	0.338889	CDC45:intron
chr22	21129710	21129788	dmr	2	0.352691	SERPIND1:intron,PI4KA:intron
chr22	24747438	24747610	dmr	1	0.266724	SPECC1L:intron
chr22	28883565	28883589	dmr	1	0.304668	TTC28:intron
chr22	31861844	31861856	dmr	2	0.30137	EIF4ENIF1:intron
chr22	36194964	36195060	dmr	2	0.341605	RBFOX2:intron
chr22	36272595	36272596	dmr	1	0.3875	RBFOX2:intron
chr22	40505773	40505935	dmr	2	0.232288	TNRC6B:intron
chr22	42042666	42042681	dmr	2	0.232877	XRCC6:intron
chr22	46081479	46081480	dmr	1	0.375	ATXN10:intron
chr3	4882398	4882399	dmr	1	0.3875	ITPR1:intron
chr3	17423791	17424028	dmr	4	0.326514	TBC1D5:intron

chr3	17469544	17469599	dmr	2	0.359617	TBC1D5:intron
chr3	17553506	17553573	dmr	2	0.317988	TBC1D5:intron
chr3	20144617	20144809	dmr	3	0.280486	KAT2B:intron
chr3	23246805	23246806	dmr	1	0.41133	UBE2E2:intron
chr3	29393691	29393692	dmr	1	0.428571	RBMS3:intron
chr3	29554149	29554179	dmr	3	0.552756	RBMS3:intron
chr3	32802232	32802233	dmr	1	0.428571	CNOT10:intron
chr3	33668489	33668490	dmr	1	0.291925	CLASP2:CDS:4
chr3	37053759	37053760	dmr	1	0.264706	MLH1:intron
chr3	37139759	37139816	dmr	2	0.31875	LRRFIP2:intron
chr3	37160851	37160852	dmr	1	0.338095	LRRFIP2:intron
chr3	38217877	38217982	dmr	2	0.318942	OXSR1:intron
chr3	42667982	42668155	dmr	3	0.318434	NKTR:intron
chr3	43451624	43451818	dmr	2	0.260831	ANO10:intron
chr3	44830760	44830841	dmr	1	0.27399	KIF15:intron
chr3	45750317	45750318	dmr	1	0.37688	SACM1L:intron
chr3	56711000	56711201	dmr	3	0.264151	FAM208A:intron
chr3	56911221	56911222	dmr	1	0.316176	ARHGEF3:intron
chr3	57819936	57820106	dmr	3	0.382313	SLMAP:intron
chr3	58848398	58848551	dmr	2	0.260236	C3orf67:intron
chr3	65632353	65632536	dmr	3	0.331381	MAGI1:intron
chr3	65937091	65937351	dmr	4	0.270136	MAGI1:intron
chr3	67672850	67672992	dmr	3	0.327689	SUCLG2:intron
chr3	71604813	71604970	dmr	2	0.24607	FOXP1:intron

chr3	78838611	78838612	dmr	1	0.293844	ROBO1:intron
chr3	78914048	78914116	dmr	2	0.334122	ROBO1:intron
chr3	81696009	81696010	dmr	1	0.279167	GBE1:intron
chr3	93729878	93729909	dmr	1	0.249714	ARL13B:intron
chr3	100012762	100012763	dmr	1	0.266667	TBC1D23:intron
chr3	100468783	100468784	dmr	1	0.368421	ABI3BP:3UTR
chr3	102169756	102169888	dmr	4	0.310904	ZPLD1:intron
chr3	105394087	105394097	dmr	2	0.340909	CBLB:intron
chr3	105417607	105417608	dmr	1	0.355349	CBLB:intron
chr3	106965727	106965897	dmr	3	0.201401	LOC344595:ncexon:2
chr3	112297673	112297674	dmr	1	0.342742	SLC35A5:intron
chr3	114363922	114364038	dmr	2	0.266601	ZBTB20:intron
chr3	114551479	114551655	dmr	2	0.25875	ZBTB20:intron
chr3	114664084	114664099	dmr	2	0.30625	ZBTB20:intron
chr3	114848212	114848267	dmr	2	0.311151	ZBTB20:intron
chr3	121460237	121460377	dmr	3	0.343067	GOLGB1:intron
chr3	121521022	121521023	dmr	1	0.519841	IQCB1:intron
chr3	121526647	121526648	dmr	1	0.255051	IQCB1:intron
chr3	123658632	123658691	dmr	2	0.409091	CCDC14:intron
chr3	126561907	126562089	dmr	3	0.290339	CHCHD6:intron
chr3	126644852	126644922	dmr	4	-0.42213	CHCHD6:intron
chr3	131060130	131060131	dmr	1	0.348291	NEK11:intron,LOC339874:intron
chr3	132232861	132232951	dmr	3	0.323822	DNAJC13:intron
chr3	132395032	132395054	dmr	3	0.252095	UBA5:intron,NPHP3-ACAD11:intron

chr3	135709329	135709513	dmr	1	0.244364	PPP2R3A:intron
chr3	137890479	137890607	dmr	2	0.304853	DBR1:CDS:3
chr3	137958483	137958484	dmr	1	0.285714	ARMC8:intron
chr3	137997494	137997678	dmr	1	0.32776	ARMC8:intron,NME9:intron
chr3	141925042	141925195	dmr	3	0.27395	GK5:intron
chr3	143221440	143221589	dmr	3	0.253822	SLC9A9:intron
chr3	145910167	145910540	dmr	4	0.29794	PLSCR4:3UTR
chr3	146174872	146174898	dmr	1	0.375834	PLSCR2:intron
chr3	148907683	148907684	dmr	1	0.285714	CP:intron
chr3	153871929	153871930	dmr	1	0.310345	ARHGEF26:intron
chr3	155493557	155493614	dmr	3	0.285038	C3orf33:CDS:2
chr3	160141068	160141226	dmr	2	0.0362903	SMC4:CDS:13
chr3	165510102	165510248	dmr	3	0.304623	BCHE:intron
chr3	170608643	170608644	dmr	1	0.317532	EIF5A2:3UTR
chr3	171340771	171340772	dmr	1	0.404255	PLD1:intron
chr3	172477701	172477831	dmr	1	0.333218	ECT2:intron
chr3	174699290	174699452	dmr	4	0.207101	NAALADL2:intron
chr3	182599590	182599629	dmr	1	0.240698	ATP11B:intron
chr3	184547647	184547648	dmr	1	0.305556	VPS8:intron
chr3	185873797	185873889	dmr	2	0.36936	DGKG:intron
chr3	188030045	188030091	dmr	3	0.358278	LPP:intron
chr3	190347524	190347525	dmr	1	0.264706	IL1RAP:3UTR
chr3	193353070	193353231	dmr	2	0.324652	OPA1:CDS:9
chr3	194918410	194918443	dmr	2	0.314611	XXYLT1:intron

chr3	195244481	195244677	dmr	1	0.286806	PPP1R2:intron
chr3	196826402	196826439	dmr	1	0.291157	DLG1:intron
chr3	196882959	196882964	dmr	2	0.286453	DLG1:intron
chr4	17892241	17892389	dmr	3	0.297529	LCORL:intron
chr4	17971019	17971347	dmr	3	0.301264	LCORL:intron
chr4	20738115	20738116	dmr	1	0.243902	KCNIP4:intron
chr4	41091897	41091976	dmr	3	0.274238	APBB2:intron
chr4	48645832	48645833	dmr	1	0.270588	FRYL:intron
chr4	54324883	54325020	dmr	3	0.282484	FIP1L1:CDS:17
chr4	56359608	56359609	dmr	1	0.305556	CLOCK:intron
chr4	56878013	56878071	dmr	2	0.321818	CEP135:CDS:20
chr4	62514932	62514933	dmr	1	0.31116	LPHN3:intron
chr4	65195407	65195408	dmr	1	0.372954	TECRL:intron
chr4	66248107	66248247	dmr	2	0.311223	EPHA5:intron
chr4	68338612	68338645	dmr	1	0.304091	CENPC1:intron
chr4	73233895	73233896	dmr	1	0.267677	ADAMTS3:intron
chr4	75399406	75399595	dmr	3	0.312715	AREG:intron
chr4	77272830	77272925	dmr	2	0.327922	CCDC158:CDS:15
chr4	78793133	78793134	dmr	1	0.307692	MRPL1:intron
chr4	79747782	79747853	dmr	3	0.30581	BMP2K:intron
chr4	85736944	85737105	dmr	2	0.272469	WDFY3:intron
chr4	86552572	86552573	dmr	1	0.352196	ARHGAP24:intron
chr4	86802539	86802540	dmr	1	0.407407	ARHGAP24:intron
chr4	86911192	86911237	dmr	2	0.321723	ARHGAP24:intron

chr4	95197500	95197521	dmr	1	0.249825	SMARCAD1:CDS:6
chr4	95197816	95198012	dmr	4	0.288182	SMARCAD1:intron
chr4	95507574	95507691	dmr	2	0.30875	PDLIM5:promoter
chr4	95507574	95507691	dmr	2	0.30875	PDLIM5:promoter,PDLIM5:5UTR
chr4	99287020	99287021	dmr	1	0.382979	RAP1GDS1:intron
chr4	99358002	99358061	dmr	3	0.320136	RAP1GDS1:intron
chr4	102042521	102042722	dmr	2	0.390476	PPP3CA:intron
chr4	102231053	102231205	dmr	2	0.247537	PPP3CA:intron
chr4	102932666	102932669	dmr	1	0.214147	BANK1:intron
chr4	103575515	103575666	dmr	3	0.250198	MANBA:intron
chr4	104035266	104035440	dmr	3	0.303905	CENPE:intron
chr4	104051320	104051502	dmr	3	0.27551	CENPE:intron
chr4	106291745	106291853	dmr	3	0.283688	PPA2:intron
chr4	107115819	107115874	dmr	3	0.3	TBCK:intron
chr4	109853638	109853639	dmr	1	0.268293	COL25A1:intron
chr4	110459351	110459352	dmr	1	0.414035	SEC24B:intron
chr4	110486966	110486967	dmr	1	0.315789	CCDC109B:intron
chr4	113187729	113187832	dmr	1	0.238884	AP1AR:CDS:8
chr4	113945014	113945015	dmr	1	0.178571	ANK2:intron
chr4	114645152	114645458	dmr	3	0.289216	CAMK2D:intron
chr4	114646147	114646183	dmr	3	0.260195	CAMK2D:intron
chr4	119837095	119837096	dmr	1	0.363354	SYNPO2:intron
chr4	120138023	120138024	dmr	1	0.222567	USP53:promoter
chr4	120509884	120510022	dmr	3	0.325459	PDE5A:intron

chr4	123226316	123226317	dmr	1	0.392157	KIAA1109:intron
chr4	124797138	124797174	dmr	2	0.292237	LOC285419:intron
chr4	128753347	128753522	dmr	2	0.317526	HSPA4L:intron
chr4	129028764	129028863	dmr	2	0.352941	LARP1B:3UTR
chr4	130028089	130028161	dmr	3	0.375458	C4orf33:intron
chr4	143763003	143763148	dmr	2	0.362804	INPP4B:intron
chr4	146790758	146790759	dmr	1	0.241881	ZNF827:intron
chr4	147346497	147346498	dmr	1	0.348837	SLC10A7:intron
chr4	151214298	151214443	dmr	3	0.401786	LRBA:intron
chr4	151287094	151287147	dmr	3	0.387996	LRBA:intron
chr4	151461049	151461098	dmr	2	0.273793	LRBA:intron
chr4	151622624	151622734	dmr	2	0.424908	LRBA:intron
chr4	151684624	151684625	dmr	1	0.333333	LRBA:intron
chr4	153271060	153271242	dmr	3	0.328231	FBXW7:CDS:2
chr4	153447728	153447769	dmr	1	0.244582	FBXW7:intron
chr4	155459285	155459389	dmr	2	0.297297	PLRG1:intron
chr4	156719475	156719670	dmr	2	0.281922	GUCY1B3:intron
chr4	156836526	156836527	dmr	1	0.444894	TDO2:intron
chr4	164452563	164452722	dmr	2	0.192395	MARCH1:intron
chr4	165131293	165131399	dmr	2	0.349008	MARCH1:intron
chr4	166239040	166239187	dmr	2	0.26186	KLHL2:CDS:14
chr4	169912856	169913011	dmr	2	0.458889	CBR4:intron
chr4	173238459	173238574	dmr	4	0.268073	GALNTL6:intron
chr4	175162241	175162242	dmr	1	0.416923	FBXO8:intron

chr4	175575051	175575052	dmr	1	0.390476	GLRA3:intron
chr4	177038584	177038624	dmr	3	0.296225	WDR17:intron
chr4	177632923	177632931	dmr	1	0.234673	VEGFC:intron
chr4	183275849	183275995	dmr	4	0.282988	ODZ3:intron
chr4	183386868	183386869	dmr	1	0.342017	ODZ3:intron
chr4	185649904	185649905	dmr	1	0.25	MLF1IP:intron
chr4	186454947	186455000	dmr	2	0.377083	PDLIM3:intron
chr4	187560903	187560921	dmr	1	0.229508	FAT1:CDS:3
chr5	365313	365514	dmr	3	0.309091	AHRR:intron
chr5	7890784	7890785	dmr	1	0.279167	MTRR:intron
chr5	19489739	19489745	dmr	1	0.351852	CDH18:intron
chr5	31410265	31410426	dmr	2	0.281378	DROSHA:intron
chr5	34708690	34708691	dmr	1	0.214286	RAI14:intron
chr5	34841264	34841401	dmr	3	0.289247	TTC23L:intron
chr5	37017890	37017891	dmr	1	0.350739	NIPBL:intron
chr5	37303391	37303482	dmr	5	0.330597	NUP155:CDS:28
chr5	37584328	37584480	dmr	3	0.253874	WDR70:intron
chr5	37701490	37701491	dmr	1	0.333333	WDR70:intron
chr5	38968287	38968295	dmr	1	0.173701	RICTOR:intron
chr5	39110318	39110495	dmr	3	0.293384	FYB:CDS:16
chr5	44812094	44812255	dmr	4	0.243594	MRPS30:CDS:3
chr5	52374440	52374634	dmr	2	0.288889	ITGA2:CDS:24
chr5	54447446	54447500	dmr	1	0.236899	CDC20B:intron
chr5	56559382	56559564	dmr	2	0.304676	GPBP1:3UTR

chr5	59776180	59776226	dmr	3	0.311258	PDE4D:intron
chr5	60422325	60422456	dmr	2	0.258732	NDUFAF2:intron
chr5	61801790	61801791	dmr	1	0.269231	IPO11:intron
chr5	61873820	61874017	dmr	2	0.306633	LRRC70:promoter
chr5	63824836	63824837	dmr	1	0.421053	RGS7BP:intron
chr5	64514764	64514960	dmr	3	0.31066	ADAMTS6:intron
chr5	64640729	64640730	dmr	1	0.275824	ADAMTS6:intron
chr5	65288860	65288952	dmr	2	0.265075	ERBB2IP:intron
chr5	65320323	65320389	dmr	2	0.348451	ERBB2IP:intron
chr5	65448206	65448334	dmr	1	0.256429	SREK1:promoter
chr5	66380744	66380830	dmr	3	0.331169	MAST4:intron
chr5	68414328	68414385	dmr	3	0.393541	SLC30A5:CDS:12
chr5	72149761	72149898	dmr	3	0.315104	TNPO1:intron
chr5	77042515	77042516	dmr	1	0.275	TBCA:intron
chr5	77043198	77043328	dmr	3	0.296438	TBCA:intron
chr5	77045683	77045684	dmr	1	0.25	TBCA:intron
chr5	77313902	77314099	dmr	4	0.20945	AP3B1:intron
chr5	77675602	77675791	dmr	1	0.309608	SCAMP1:intron
chr5	80335711	80335760	dmr	3	0.362003	RASGRF2:intron
chr5	80600909	80601081	dmr	3	0.264368	ZCCHC9:CDS:1
chr5	81031443	81031644	dmr	3	0.298427	SSBP2:intron
chr5	82870875	82870876	dmr	1	0.343137	VCAN:intron
chr5	83378764	83378819	dmr	3	0.290337	EDIL3:intron
chr5	87519992	87519993	dmr	1	0.3125	TMEM161B:intron

chr5	88165684	88165802	dmr	3	0.358453	MEF2C:intron
chr5	89910457	89910458	dmr	1	0.377778	GPR98:intron
chr5	90256209	90256237	dmr	2	0.260141	GPR98:intron
chr5	93311558	93311713	dmr	1	0.227013	FAM172A:intron
chr5	96015595	96015643	dmr	3	0.309973	CAST:intron
chr5	98223843	98224001	dmr	3	0.233083	CHD1:CDS:16
chr5	100181354	100181462	dmr	2	0.383574	ST8SIA4:intron
chr5	102300572	102300708	dmr	2	0.347491	PAM:intron
chr5	102423675	102423809	dmr	4	0.311671	GIN1:CDS:7
chr5	102525530	102525629	dmr	2	0.252405	PPIP5K2:intron
chr5	106937739	106937749	dmr	1	0.308608	EFNA5:intron
chr5	107252567	107252652	dmr	2	0.35097	FBXL17:intron
chr5	107652193	107652194	dmr	1	0.531818	FBXL17:intron
chr5	108087585	108087586	dmr	1	0.250947	FER:intron
chr5	108114203	108114204	dmr	1	0.3	FER:intron
chr5	108382595	108382783	dmr	2	0.359677	FER:intron
chr5	108718672	108718682	dmr	2	0.312985	PJA2:intron
chr5	110569198	110569257	dmr	3	0.270561	CAMK4:intron
chr5	111151204	111151405	dmr	3	0.261546	NREP:intron
chr5	112861702	112861841	dmr	3	0.34385	YTHDC2:intron
chr5	112901382	112901545	dmr	2	0.210852	YTHDC2:intron
chr5	112924816	112924918	dmr	2	0.407295	YTHDC2:intron
chr5	118437636	118437637	dmr	1	0.341615	DMXL1:CDS:3
chr5	118488441	118488442	dmr	1	0.449134	DMXL1:intron

chr5	118642792	118642793	dmr	1	0.277778	TNFAIP8:intron
chr5	118859070	118859319	dmr	4	0.33504	HSD17B4:intron
chr5	121309763	121310112	dmr	2	0.253497	SRFBP1:CDS:2
chr5	122866774	122866969	dmr	3	0.34558	CSNK1G3:intron
chr5	122931286	122931473	dmr	2	0.31746	CSNK1G3:intron
chr5	125705096	125705119	dmr	2	0.364464	GRAMD3:intron
chr5	127638762	127638934	dmr	3	0.297006	FBN2:CDS:46
chr5	127850357	127850483	dmr	3	0.25194	FBN2:intron
chr5	128878827	128879024	dmr	3	0.29907	ADAMTS19:intron
chr5	129279117	129279118	dmr	1	0.327869	CHSY3:intron
chr5	132578746	132578880	dmr	1	0.192308	FSTL4:intron
chr5	134007520	134007521	dmr	1	0.363636	SEC24A:CDS:4
chr5	134149934	134150117	dmr	3	0.308537	DDX46:intron
chr5	134287770	134287800	dmr	1	0.305921	PCBD2:intron
chr5	135270165	135270214	dmr	1	0.287582	FBXL21:promoter
chr5	139884964	139884965	dmr	1	0.342857	ANKHD1-EIF4EBP3:intron,ANKHD1:intron
chr5	145602516	145602519	dmr	2	0.315205	RBM27:intron
chr5	145890839	145890840	dmr	1	0.359046	TCERG1:3UTR
chr5	149386391	149386406	dmr	1	0.305621	HMGXB3:intron
chr5	157077683	157077684	dmr	1	0.326991	SOX30:intron
chr5	159832900	159832901	dmr	1	0.30119	SLU7:intron
chr5	162918071	162918135	dmr	1	0.237473	HMMR:CDS:15
chr5	170343135	170343352	dmr	3	0.256081	RANBP17:intron
chr5	170597069	170597192	dmr	3	0.258055	RANBP17:CDS:15

chr5	179747994	179748061	dmr	2	0.247917	GFPT2:intron
chr6	1830911	1831092	dmr	2	0.329787	GMDS:intron
chr6	2156216	2156217	dmr	1	0.25	GMDS:intron
chr6	4866335	4866424	dmr	2	0.233071	CDYL:intron
chr6	7296318	7296319	dmr	1	0.442958	SSR1:intron
chr6	7601612	7601720	dmr	3	0.318474	SNRNP48:CDS:5
chr6	8420036	8420066	dmr	2	0.366815	SLC35B3:intron
chr6	21165645	21165846	dmr	1	0.283391	CDKAL1:intron
chr6	24518776	24518777	dmr	1	0.35576	ALDH5A1:intron
chr6	43754057	43754276	dmr	2	0.275966	VEGFA:3UTR
chr6	46113367	46113529	dmr	2	0.360577	ENPP4:3UTR
chr6	55389037	55389038	dmr	1	0.357143	HMGCLL1:intron
chr6	56338749	56338850	dmr	1	0.212993	DST:CDS:74
chr6	57059368	57059517	dmr	3	0.358161	RAB23:intron
chr6	64394226	64394235	dmr	1	0.178864	PHF3:CDS:3
chr6	64420834	64420835	dmr	1	0.416667	PHF3:intron
chr6	66293169	66293371	dmr	3	0.309524	EYS:intron
chr6	70631698	70631778	dmr	1	0.225758	COL19A1:intron
chr6	71203050	71203229	dmr	3	0.313787	FAM135A:intron
chr6	71501033	71501192	dmr	4	0.300151	SMAP1:intron
chr6	76350225	76350226	dmr	1	0.346154	SENP6:intron
chr6	79784778	79784792	dmr	1	0.262424	PHIP:intron
chr6	80384909	80385038	dmr	3	0.293831	SH3BGRL2:intron
chr6	80746707	80746708	dmr	1	0.268293	TTK:intron

chr6	80926335	80926537	dmr	2	0.260895	BCKDHB:intron
chr6	82458808	82458917	dmr	2	0.277062	FAM46A:3UTR
chr6	83610703	83610878	dmr	3	0.298607	UBE3D:intron
chr6	84896958	84897047	dmr	3	0.272894	KIAA1009:intron
chr6	84922710	84922925	dmr	2	0.214581	KIAA1009:CDS:5
chr6	89386358	89386359	dmr	1	0.30848	RNGTT:intron
chr6	89423369	89423496	dmr	3	0.273812	RNGTT:intron
chr6	90047011	90047022	dmr	2	0.369231	UBE2J1:intron
chr6	90856586	90856754	dmr	2	0.303828	BACH2:intron
chr6	99848488	99848495	dmr	1	0.287913	PNISR:CDS:10
chr6	99909855	99910029	dmr	2	0.27673	USP45:intron
chr6	100987614	100987615	dmr	1	0.252252	ASCC3:intron
chr6	101060698	101060714	dmr	2	0.366667	ASCC3:intron
chr6	101152149	101152203	dmr	1	0.221538	ASCC3:intron
chr6	102177554	102177702	dmr	1	0.237374	GRIK2:intron
chr6	105182183	105182366	dmr	3	0.292255	HACE1:intron
chr6	106649644	106649729	dmr	3	0.350538	ATG5:intron
chr6	107743827	107743828	dmr	1	0.454545	PDSS2:intron
chr6	110464981	110465127	dmr	2	0.293153	WASF1:intron
chr6	110482244	110482350	dmr	1	0.275116	WASF1:promoter
chr6	111656065	111656114	dmr	2	0.283017	REV3L:intron
chr6	112402335	112402336	dmr	1	0.324324	TUBE1:CDS:5
chr6	125336606	125336688	dmr	1	0.331665	RNF217:intron
chr6	128454405	128454534	dmr	3	0.312112	PTPRK:intron

chr6	128475715	128475716	dmr	1	0.227731	PTPRK:intron
chr6	128629288	128629595	dmr	2	0.431507	PTPRK:intron
chr6	129506928	129507153	dmr	3	0.304744	LAMA2:intron
chr6	129555901	129555971	dmr	3	0.328125	LAMA2:intron
chr6	131296226	131296227	dmr	1	0.295455	EPB41L2:intron
chr6	134322566	134322719	dmr	3	0.35081	SLC2A12:intron
chr6	135802780	135802970	dmr	2	0.258716	AHI1:intron
chr6	139459434	139459435	dmr	1	0.27561	HECA:intron
chr6	143954882	143954979	dmr	2	0.299663	PHACTR2:intron
chr6	146234629	146234630	dmr	1	0.27864	SHPRH:CDS:23
chr6	147119773	147119774	dmr	1	0.270833	ADGB:intron
chr6	147303703	147303879	dmr	2	0.326395	LOC729178:intron
chr6	149674683	149674832	dmr	3	0.261458	TAB2:intron
chr6	152707781	152707942	dmr	3	0.25281	SYNE1:intron
chr6	155098378	155098440	dmr	2	0.245283	SCAF8:intron
chr6	157156562	157156736	dmr	2	0.306366	ARID1B:intron
chr6	157501270	157501314	dmr	2	0.394737	ARID1B:intron
chr6	159025828	159025897	dmr	3	0.343817	TMEM181:intron
chr6	159596114	159596115	dmr	1	0.339161	FNDC1:intron
chr6	160157271	160157422	dmr	2	0.336985	WTAP:promoter
chr6	160157271	160157422	dmr	2	0.336985	WTAP:promoter,WTAP:5UTR
chr6	161853966	161854026	dmr	2	0.277406	PARK2:intron
chr7	3944838	3944925	dmr	4	0.344444	SDK1:intron
chr7	7572439	7572462	dmr	3	0.367685	COL28A1:promoter

chr7	11158936	11158937	dmr	1	0.27032	PHF14:intron
chr7	15576442	15576473	dmr	1	0.298063	AGMO:intron
chr7	18769533	18769709	dmr	2	0.386946	HDAC9:intron
chr7	18936938	18937127	dmr	3	0.333353	HDAC9:intron
chr7	24652147	24652271	dmr	3	0.399397	MPP6:intron
chr7	24657054	24657055	dmr	1	0.294009	MPP6:intron
chr7	24676496	24676521	dmr	1	0.265893	MPP6:intron
chr7	32756995	32757042	dmr	3	0.396369	DPY19L1P1:intron
chr7	34994074	34994140	dmr	2	0.326812	DPY19L1:intron
chr7	36238126	36238214	dmr	3	0.337321	EEPD1:intron
chr7	40543499	40543500	dmr	1	0.431034	C7orf10:intron
chr7	45794411	45794594	dmr	3	0.374627	SEPT7P2:intron
chr7	69541076	69541182	dmr	1	0.301341	AUTS2:intron
chr7	73675539	73675540	dmr	1	0.316197	PMS2L2:intron,GTF2IP1:intron,SPDYE8P:intron,LOC100093631:intron
chr7	77365945	77366067	dmr	3	0.31492	RSBN1L:intron
chr7	78787714	78787715	dmr	1	0.317227	MAGI2:intron
chr7	78846320	78846321	dmr	1	0.362552	MAGI2:intron
chr7	81352243	81352402	dmr	4	0.397034	HGF:intron
chr7	81603354	81603700	dmr	3	0.34268	CACNA2D1:intron
chr7	81731068	81731079	dmr	2	0.2874	CACNA2D1:intron
chr7	82042416	82042499	dmr	3	0.288495	CACNA2D1:intron
chr7	82445079	82445273	dmr	3	0.312359	PCLO:intron
chr7	82768404	82768405	dmr	1	0.44186	PCLO:intron
chr7	84706674	84706839	dmr	3	0.386427	SEMA3D:intron

chr7	84727671	84727672	dmr	1	0.40119	SEMA3D:intron
chr7	91692437	91692485	dmr	3	0.315679	AKAP9:intron
chr7	91715521	91715657	dmr	4	0.259717	AKAP9:CDS:37
chr7	91782502	91782674	dmr	3	0.237226	LRRD1:intron
chr7	91934317	91934318	dmr	1	0.407407	ANKIB1:intron
chr7	92029228	92029315	dmr	1	0.281355	ANKIB1:3UTR
chr7	92414219	92414419	dmr	3	0.253999	CDK6:intron
chr7	93626928	93627059	dmr	3	0.289386	BET1:intron
chr7	94259661	94259720	dmr	3	0.338252	SGCE:intron
chr7	102554355	102554456	dmr	1	0.36741	LRRC17:intron,FBXL13:intron
chr7	107052974	107052975	dmr	1	0.347557	COG5:CDS:7
chr7	107388794	107388940	dmr	3	0.328512	CBLL1:intron
chr7	110972508	110972509	dmr	1	0.377735	IMMP2L:intron
chr7	111467491	111467492	dmr	1	0.291667	DOCK4:intron
chr7	114309348	114309527	dmr	3	0.277517	FOXP2:intron
chr7	114315464	114315465	dmr	1	0.308204	FOXP2:intron
chr7	116764182	116764272	dmr	2	0.300647	ST7-AS2:intron,ST7:intron
chr7	120436018	120436078	dmr	2	0.484484	TSPAN12:intron
chr7	120973276	120973387	dmr	3	0.304077	WNT16:intron
chr7	124386528	124386690	dmr	3	0.271118	GPR37:CDS:2
chr7	124499996	124500118	dmr	2	0.285636	POT1:intron
chr7	129840070	129840272	dmr	4	0.265788	TMEM209:intron
chr7	132996498	132996499	dmr	1	0.239049	EXOC4:intron
chr7	133151729	133151869	dmr	2	0.297342	EXOC4:intron

chr7	135333145	135333310	dmr	4	0.306527	NUP205:CDS:43
chr7	137240365	137240556	dmr	3	0.304753	DGKI:intron
chr7	143000379	143000417	dmr	2	0.277926	CASP2:intron
chr7	144525481	144525661	dmr	3	0.345705	TPK1:intron
chr7	147130224	147130380	dmr	3	0.27105	CNTNAP2:intron,MIR548I4:intron
chr7	148441664	148441716	dmr	3	0.405538	CUL1:intron
chr7	148454137	148454209	dmr	1	0.148494	CUL1:CDS:3
chr7	149179189	149179221	dmr	1	0.259804	ZNF746:intron
chr7	151868824	151869015	dmr	3	0.262655	MLL3:intron
chr8	4082838	4082918	dmr	2	0.359585	CSMD1:intron
chr8	6311110	6311346	dmr	3	0.278579	MCPH1:intron
chr8	6419856	6419857	dmr	1	0.454545	ANGPT2:intron,MCPH1:intron
chr8	13055874	13055875	dmr	1	0.290323	DLC1:intron
chr8	13325516	13325553	dmr	2	0.280247	DLC1:intron
chr8	15440687	15440898	dmr	3	0.302588	TUSC3:intron
chr8	15480636	15480739	dmr	5	0.311448	TUSC3:CDS:2
chr8	17797828	17797829	dmr	1	0.316176	PCM1:intron
chr8	17803368	17803526	dmr	2	0.275715	PCM1:intron
chr8	28750416	28750556	dmr	2	0.279735	HMBOX1:intron
chr8	28866973	28867033	dmr	3	0.33779	HMBOX1:intron
chr8	30275458	30275488	dmr	1	0.269859	RBPMS:intron
chr8	30888578	30888579	dmr	1	0.3125	PURG:ncexon:1
chr8	30973215	30973282	dmr	3	0.337366	WRN:intron
chr8	31022586	31022587	dmr	1	0.289696	WRN:intron

chr8	39080572	39080612	dmr	1	0.236559	ADAM32:CDS:14
chr8	39180255	39180256	dmr	1	0.444444	ADAM5P:intron
chr8	42915082	42915271	dmr	1	0.338993	FNTA:intron
chr8	48341206	48341355	dmr	3	0.420465	KIAA0146:intron
chr8	48776000	48776001	dmr	1	0.384848	PRKDC:CDS:42
chr8	50961706	50961793	dmr	3	0.363475	SNTG1:intron
chr8	51068834	51068926	dmr	2	0.322967	SNTG1:intron
chr8	51227620	51227621	dmr	1	0.206897	SNTG1:intron
chr8	51535303	51535483	dmr	2	0.338725	SNTG1:intron
chr8	53048360	53048582	dmr	3	0.282803	ST18:intron
chr8	53577689	53577876	dmr	3	0.307635	RB1CC1:intron
chr8	58196080	58196170	dmr	3	0.351222	C8orf71:intron
chr8	62351409	62351410	dmr	1	0.381944	CLVS1:intron
chr8	62495511	62495628	dmr	2	0.238511	ASPH:intron
chr8	62586676	62586743	dmr	3	0.260832	ASPH:intron
chr8	64085558	64085663	dmr	1	0.273413	YTHDF3:intron
chr8	66525389	66525479	dmr	1	0.188378	ARMC1:CDS:3
chr8	66638419	66638510	dmr	2	0.372619	PDE7A:intron
chr8	72272445	72272446	dmr	1	0.387446	EYA1:intron
chr8	74461507	74461648	dmr	3	0.301513	STAU2:intron
chr8	77574696	77574768	dmr	1	0.31348	LOC100192378:intron
chr8	77583101	77583501	dmr	5	0.295665	LOC100192378:intron
chr8	77701967	77702223	dmr	3	0.299047	ZFHX4:intron
chr8	87486359	87486446	dmr	2	0.32368	FAM82B:3UTR

chr8	90990717	90990718	dmr	1	0.28125	NBN:intron
chr8	95524192	95524347	dmr	2	0.376829	KIAA1429:CDS:12
chr8	99475996	99476046	dmr	2	0.319809	STK3:intron
chr8	99528896	99529063	dmr	3	0.257188	STK3:intron
chr8	99634808	99634941	dmr	3	0.282544	STK3:intron
chr8	99744391	99744392	dmr	1	0.311275	STK3:intron
chr8	100190812	100190813	dmr	1	0.28125	VPS13B:intron
chr8	101589288	101589289	dmr	1	0.386667	SNX31:CDS:13
chr8	106683539	106683665	dmr	2	0.433284	ZFPM2:intron
chr8	107734704	107734705	dmr	1	0.522727	OXR1:intron
chr8	107771758	107771863	dmr	3	0.316249	ABRA:3UTR
chr8	110302134	110302135	dmr	1	0.342857	NUDCD1:CDS:5
chr8	121320127	121320259	dmr	3	0.314123	COL14A1:intron
chr8	121430752	121430896	dmr	3	0.36008	MRPL13:intron
chr8	121794883	121794981	dmr	2	0.283943	SNTB1:intron
chr8	124137488	124137631	dmr	4	0.304596	WDR67:intron
chr8	126055899	126056088	dmr	2	0.266426	KIAA0196:CDS:22
chr8	131131146	131131147	dmr	1	0.381944	ASAP1:intron
chr8	141725063	141725064	dmr	1	0.397368	PTK2:intron
chr8	144810244	144810353	dmr	2	0.662338	FAM83H:CDS:4
chr9	2056203	2056232	dmr	2	0.256805	SMARCA2:intron
chr9	4237231	4237293	dmr	2	0.339269	GLIS3:intron
chr9	5675547	5675594	dmr	2	0.31565	KIAA1432:intron
chr9	6481970	6482014	dmr	3	0.278516	UHRF2:CDS:8

chr9	14100920	14100921	dmr	1	0.373377	NFIB:intron
chr9	14119424	14119582	dmr	3	0.324324	NFIB:intron
chr9	14648720	14648721	dmr	1	0.35	ZDHHC21:intron
chr9	16638708	16638823	dmr	2	0.31628	BNC2:intron
chr9	17221318	17221466	dmr	3	0.351987	CNTLN:intron
chr9	17275066	17275169	dmr	2	0.358641	CNTLN:intron
chr9	17296539	17296579	dmr	1	0.334783	CNTLN:intron
chr9	18641686	18641687	dmr	1	0.304615	ADAMTSL1:intron
chr9	36360280	36360281	dmr	1	0.323718	RNF38:intron
chr9	37231444	37231445	dmr	1	0.425	ZCCHC7:intron
chr9	66606218	66606246	dmr	1	0.229148	LOC286297:intron,ANKRD20A3:intron
chr9	72878871	72878877	dmr	2	0.39	SMC5:intron
chr9	79911788	79912153	dmr	2	0.325239	VPS13A:intron
chr9	79964286	79964451	dmr	2	0.315942	VPS13A:intron
chr9	80394672	80394677	dmr	1	0.266294	GNAQ:intron
chr9	80865843	80866118	dmr	2	0.309154	CEP78:intron
chr9	95144528	95144697	dmr	3	0.378015	CENPP:intron
chr9	101177404	101177597	dmr	4	0.289565	GABBR2:intron
chr9	106864345	106864656	dmr	3	0.307261	SMC2:CDS:7
chr9	107519827	107520130	dmr	2	0.283333	NIPSNAP3A:intron
chr9	107544506	107544507	dmr	1	0.297555	ABCA1:3UTR
chr9	108074722	108074723	dmr	1	0.294958	SLC44A1:intron
chr9	108138192	108138193	dmr	1	0.375	SLC44A1:intron
chr9	111792623	111792624	dmr	1	0.290323	C9orf5:intron

chr9	112206918	112206919	dmr	1	0.305668	PTPN3:intron
chr9	113678080	113678134	dmr	3	0.425018	LPAR1:intron
chr9	115963948	115963949	dmr	1	0.285714	FKBP15:intron
chr9	115963985	115964041	dmr	2	0.286927	FKBP15:intron
chr9	117220316	117220345	dmr	2	0.309091	DFNB31:intron
chr9	125802979	125802980	dmr	1	0.213489	RABGAP1:intron
chr9	126577972	126578100	dmr	3	0.257622	DENND1A:intron
chr9	127694128	127694129	dmr	1	0.231419	GOLGA1:intron
chr9	128333180	128333252	dmr	2	0.366752	MAPKAP1:intron
chr9	128363540	128363656	dmr	2	0.217544	MAPKAP1:intron
chr9	128530678	128530762	dmr	3	0.295749	PBX3:intron
chr9	128636444	128636445	dmr	1	0.351744	PBX3:intron
chr9	129592171	129592246	dmr	2	0.428951	ZBTB43:intron
chrX	9880813	9880948	dmr	6	0.652047	SHROOM2:intron
chrX	33152956	33152957	dmr	1	0.583333	DMD:intron
chrX	114879217	114879367	dmr	3	0.364498	PLS3:CDS:10
chrX	132952123	132952166	dmr	3	0.195122	GPC3:intron
chrY	6404063	6404611	dmr	10	0.360326	TTTY1B:intron,TTTY2B:intron,TTTY1:intron,TTTY8:intron,TTTY23:intron,TTTY7B:intron,TTTY8B:intron,TTTY7:intron,TTTY21:intron,TTTY23B:intron,TTTY2:intron,TTTY21B:intron
chrY	6405369	6405754	dmr	4	0.287802	TTTY1B:intron,TTTY2B:intron,TTTY1:intron,TTTY8:intron,TTTY23:intron,TTTY7B:intron,TTTY8B:intron,TTTY7:intron,TTTY21:intron,TTTY23B:intron,TTTY2:intron,TTTY21B:intron
chrY	16865593	16865786	dmr	3	+	NLGN4Y:intron

Supplementary Table 8: The genes with DMRs which located in the gene promoter by Radmeth methods analysis

gene symbol
COL28A1
FBXL21
FOXJ3
FPGT
FPGT-TNNI3K
HNRNPA1L2
IDH1
LRRC70
PDLIM5
RGS13
SLC38A1
SNRPN
SNURF
SREK1
SYT1
USP53
WASF1
WTAP
ZNF268
ZNF280D
